# Mutations in Four Glycosyl Hydrolases Reveal a Highly Coordinated Pathway for Rhodopsin Biosynthesis and N-Glycan Trimming in *Drosophila melanogaster*

**DOI:** 10.1371/journal.pgen.1004349

**Published:** 2014-05-01

**Authors:** Erica E. Rosenbaum, Eva Vasiljevic, Kimberley S. Brehm, Nansi Jo Colley

**Affiliations:** Department of Ophthalmology & Visual Sciences and Department of Genetics, University of Wisconsin-Madison, Madison, Wisconsin, United States of America; New York University, United States of America

## Abstract

As newly synthesized glycoproteins move through the secretory pathway, the asparagine-linked glycan (N-glycan) undergoes extensive modifications involving the sequential removal and addition of sugar residues. These modifications are critical for the proper assembly, quality control and transport of glycoproteins during biosynthesis. The importance of N-glycosylation is illustrated by a growing list of diseases that result from defects in the biosynthesis and processing of N-linked glycans. The major rhodopsin in *Drosophila melanogaster* photoreceptors, Rh1, is highly unique among glycoproteins, as the N-glycan appears to be completely removed during Rh1 biosynthesis and maturation. However, much of the deglycosylation pathway for Rh1 remains unknown. To elucidate the key steps in Rh1 deglycosylation *in vivo*, we characterized mutant alleles of four *Drosophila* glycosyl hydrolases, namely α-mannosidase-II (α-Man-II), α-mannosidase-IIb (α-Man-IIb), a β-*N*-acetylglucosaminidase called fused lobes (Fdl), and hexosaminidase 1 (Hexo1). We have demonstrated that these four enzymes play essential and unique roles in a highly coordinated pathway for oligosaccharide trimming during Rh1 biosynthesis. Our results reveal that α-Man-II and α-Man-IIb are *not* isozymes like their mammalian counterparts, but rather function at distinct stages in Rh1 maturation. Also of significance, our results indicate that Hexo1 has a biosynthetic role in N-glycan processing during Rh1 maturation. This is unexpected given that in humans, the hexosaminidases are typically lysosomal enzymes involved in N-glycan catabolism with no known roles in protein biosynthesis. Here, we present a genetic dissection of glycoprotein processing in *Drosophila* and unveil key steps in N-glycan trimming during Rh1 biosynthesis. Taken together, our results provide fundamental advances towards understanding the complex and highly regulated pathway of N-glycosylation *in vivo* and reveal novel insights into the functions of glycosyl hydrolases in the secretory pathway.

## Author Summary

As newly synthesized glycoproteins move through the secretory pathway, the asparagine-linked glycan (N-glycan) undergoes extensive modifications involving the sequential removal and addition of sugar residues. These modifications are critical for the proper assembly, quality control and transport of glycoproteins during biosynthesis. The importance of N-glycosylation is illustrated by a growing list of diseases that result from defects in the biosynthesis and processing of N-linked glycans. The major rhodopsin in the *Drosophila* (fruit fly) eye, Rh1, is highly unique among glycoproteins, as the N-glycan appears to be completely removed during Rh1 biosynthesis and maturation. However, much of the deglycosylation pathway for Rh1 remains unknown. To elucidate the key steps in Rh1 deglycosylation, we conducted a genetic dissection of glycoprotein processing *in vivo*. We have demonstrated that four glycosyl hydrolases play essential and unique roles in a highly coordinated pathway for N-glycan trimming during Rh1 biosynthesis. Our results reveal novel insights into the functions of glycosyl hydrolases in the secretory pathway and provide fundamental advances towards understanding N-glycosylation *in vivo*.

## Introduction

**Figure 1:**
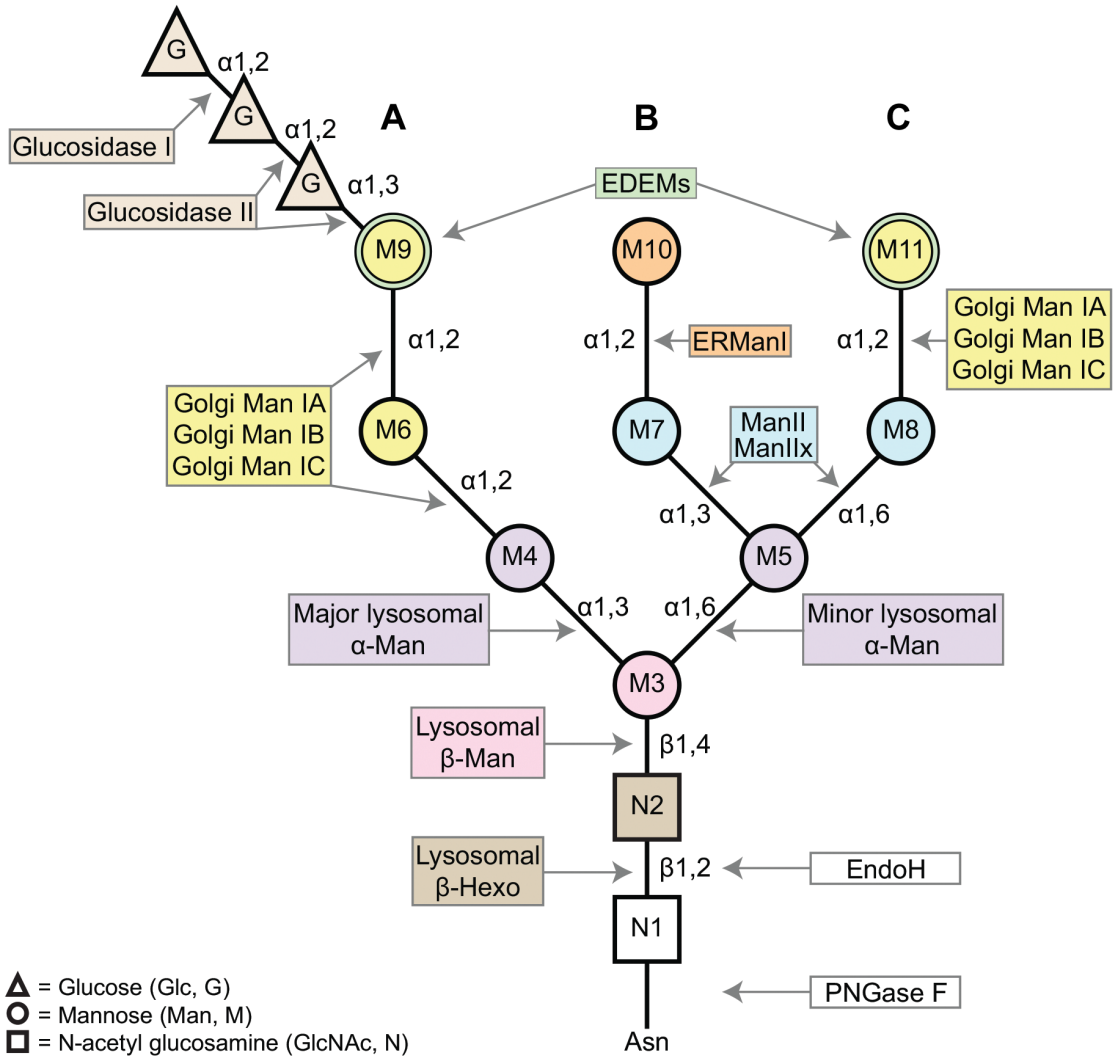
A schematic of the asparagine (Asn, N)-linked oligosaccharide precursor, Glc_3_Man_9_GlcNAc_2_. The N-glycan contains three glucose residues (triangles, G), nine mannose residues (circles, M3–11), and two β-*N*-acetylglucosamine residues (squares, N1–2). A, B, and C refer to the major branches on the N-glycan structure. Colors complement the enzyme list provided in Figure 2, indicating the glycosyl hydrolases (GH) responsible for trimming each residue. The α1,2-linked mannose residues (M6, M9, M10, and M11) are removed by Class I α-mannosidases (GH Family 47). The α1,3- and α1,6-linked mannose residues (M4, M5, M7, and M8) are removed by Class II α-mannosidases (GH Family 38). M3 is attached to the N-glycan by a β1,4-linkage and is removed by a β-mannosidase (GH Family 2). Finally, the terminal GlcNAc residue is attached via a β1,2-linkage and is removed by a β-hexosaminidase (GH Family 20). Man (mannosidase), Hexo (hexosaminidase).

Many proteins that are synthesized in the endoplasmic reticulum (ER) and transported through the secretory pathway are posttranslationally modified with an asparagine (N)-linked carbohydrate moiety, termed an N-glycan (Figure 1). N-glycosylation is key to the proper assembly, quality control, and transport of glycoproteins during biosynthesis [1], [2]. Not only does the N-glycan directly reduce protein aggregation, but it also serves as a signal for interaction with key lectins and glycosyl hydrolases. N-glycosylation begins in the ER, where preformed Glc_3_Man_9_GlcNAc_2_ (Figure 1) is transferred, *en bloc*, to asparagine (Asn) residues on nascent polypeptide chains containing the consensus sequence, NXS/T [3], [4]. As the newly synthesized glycoprotein moves through the secretory pathway, the oligosaccharide precursor undergoes extensive modifications involving sequential removal and addition of sugar residues in the Golgi [5]. All of the enzymes that function in carbohydrate trimming during biosynthesis are exoglycosidases and thus require their substrate residue to be exposed at a terminal position on the N-glycan. Accordingly, carbohydrate trimming proceeds sequentially, with the most terminal residues being removed first. These modifications serve critical functions in glycoprotein recognition, folding, targeting, and degradation [1], [6].

**Figure 2:**
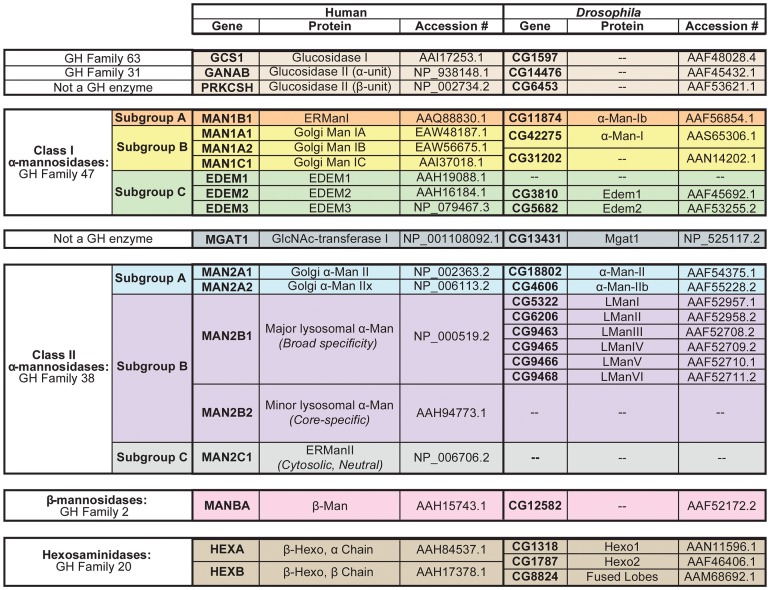
N-glycan processing enzymes in humans and *Drosophila*. Human and *Drosophila* enzymes in numerous glycosyl hydrolase (GH) families are involved in the processing and/or catabolism of N-glycans. These proteins are divided into six major groups and are listed in the order in which they are thought to function in the cascade (See Figure 4). (**1**) Glucosidase I and the α-subunit of glucosidase II are from GH Families 63 and 31, respectively (tan). The β-subunit of glucosidase II is not a GH enzyme (tan). (**2**) The Class I α-mannosidases from GH Family 47 can be classified into three functionally distinct subgroups: Subgroup A includes the ER α1,2-mannosidases (orange), Subgroup B includes the Golgi α1,2-mannosidases (yellow), and Subgroup C includes the EDEMs (green). (**3**) GlcNAc-transferase is not a GH enzyme (dark blue). (**4**) The Class II α-mannosidases from GH Family 38 can also be classified into several functionally distinct subgroups: Subgroup A includes the Golgi α1,3(1,6)-mannosidases (light blue), Subgroup B includes the lysosomal α-mannosidases (purple), and Subgroup C includes an ER/cytosolic α-mannosidase, which is not found in *Drosophila* (grey). (**5**) The β-mannosidases from GH Family 2 (pink) and (**6**) the hexosaminidases from GH Family 20 (brown) are also listed. Accession numbers presented here indicate the protein sequences that were used for all amino acid alignments and sequence analyses performed in this study. Man (mannosidase), Hexo (hexosaminidase), L (lysosomal).

N-glycosylation in *Drosophila melanogaster* proceeds in a similar fashion to the well-characterized glycosylation pathways identified in mammalian systems, beginning with addition of the Glc_3_Man_9_GlcNAc_2_ precursor to newly synthesized proteins in the ER [2], [7]–[9]. Likewise, steps in the subsequent processing and trimming of the N-glycan are highly conserved between humans and *Drosophila* [2]. Here, we show that there is at least one *Drosophila* homolog for nearly all of the human glycosyl hydrolase (GH) enzymes involved in N-glycosylation (Figures 2 and S2–S7).

One notable difference between vertebrate and invertebrate glycan processing is reflected in the relative abundance of the final N-glycan structures produced on glycoproteins [2]. In vertebrates, glycoproteins typically harbor hybrid- or complex-type glycan structures, in which one or two additional N-acetylglucosamine (GlcNAc) residues serve as the building blocks for the elongation and elaboration of the N-glycan. In contrast, glycoproteins in *Drosophila* and other invertebrates typically have high- or pauci-mannosidic glycan structures, in which the GlcNAc residues are removed prior to elongation, and thus elaboration of the N-glycan begins directly from the trimannosyl core. These differences are explained by the presence of a β-*N*-acetylglucosaminidase in *Drosophila*, called fused lobes (Fdl), which functions to remove the terminal GlcNAc residue. Accordingly, mutations in *Drosophila fused lobes* lead to the formation of the hybrid- and complex-type glycans that are more typical of vertebrates [10].

Mutations in other *Drosophila* glycosyl hydrolases that function in the N-glycan processing pathway have also been described *in vivo*. The first α-mannosidase to be cloned and characterized in *Drosophila* was α-mannosidase-I (α-Man-I, G42275) [7]. Null mutations in *α-man-I* (also known as *mas1*) were shown to cause developmental defects in the embryonic peripheral nervous system (PNS), in the wing, and in the adult eye [7]. It was recently shown that α-mannosidase-II (α-Man-II, CG18802) and α-mannosidase-IIb (α-Man-IIb, CG4606) are required for rhodopsin deglycosylation in *Drosophila* [11]. The authors further demonstrated that α-Man-II activity is strictly regulated by a phosphatase, *Drosophila* metallophosphoesterase (dMPPE). In addition to mutant analysis, *Drosophila* α-Man-II has been the subject of extensive structural analysis over the last decade. Its crystal structure now serves as the standard model for all Class II α-mannosidases [12]–[22]. While some of the GH enzymes involved in N-glycosylation have been isolated and well characterized in *Drosophila*, many remain unannotated and/or uncharacterized.

The importance of N-glycosylation is illustrated by a growing list of diseases that result from defects in the biosynthesis and processing of N-linked glycans. Congenital disorders of glycosylation (CDG) are a group of autosomal recessive diseases that result from defects in the synthesis, attachment, and/or processing of glycans [23]. Patients with CDG manifest a variety of symptoms and many of these diseases affect the eye. Patients with CDG Type Ia and Ic may develop progressive vision loss, retinitis pigmentosa (RP), impaired night vision, convergent strabismus, abnormal eye movements, as well as other ocular pathologies [24]–[26]. Several additional diseases have been associated with defects in N-glycan processing, such as α-mannosidosis and β-mannosidosis, which are autosomal recessive lysosomal storage diseases caused by genetic disruption of an α-mannosidase (MAN2B1) or β-mannosidase (MANB1) enzyme, respectively [27], [28] (Figure 2). These disorders are characterized by the accumulation of undegraded N-glycan products, which retain either α- or β-linked mannose residues, respectively. Patients often suffer from mental retardation, among a variety of other clinical manifestations [27], [29]. Other symptoms include late-onset retinal dystrophy, peripheral neuropathy, hearing loss, speech impairment, epileptic encephalopathy, angiokeratome, and skeletal/facial dysmorphism [30], [31]. Finally, two loci encoding the hexosaminidase enzymes, HEXA and HEXB (Figure 2), have been directly linked to Tay-Sachs and Sandhoff disease [32].

There is an overwhelming diversity of oligosaccharide structures that can be generated during glycoprotein processing, thus determining the roles of distinct oligosaccharide structures *in vivo* is a formidable challenge. Limited clues have come from studies of cell lines that are defective in certain processing enzymes [33]. However, these studies present some limitations, as cell culture experiments do not always mimic the complex environments that exist *in vivo*, in multi-cellular organisms. Other insights have come from studies involving the treatment of cells or animals with inhibitors that block various enzymatic steps in N-glycan processing [34]. The caveat with these studies is that the inhibitors often target multiple enzymes, complicating interpretation of the results [35]. One of the most powerful techniques for elucidating the specific roles of individual processing enzymes in animals involves the generation and analysis of the corresponding mutants *in vivo*. Such a systematic genetic dissection of N-glycosylation is entirely feasible in *Drosophila*. Additionally, the eye is not required for viability and therefore null mutations in the eye can be easily investigated to uncover the roles of glycosyl hydrolases *in vivo*.

In this study, we identify four glycosyl hydrolases in *Drosophila* that are critical for N-glycan processing during protein biosynthesis, namely α-mannosidase-II, α-mannosidase-IIb, a β-*N*-acetylglucosaminidase called fused lobes, and hexosaminidase 1. We have used the major visual protein in *Drosophila*, rhodopsin 1 (Rh1), as a substrate for the identification and characterization of mutants that are defective in specific N-glycan processing enzymes in the eye. Rh1 is highly unique among glycoproteins as we, and others, have shown that the N-glycan is extensively trimmed as Rh1 matures through the secretory pathway during biosynthesis [36]–[39]. Here, we demonstrate that Rh1 deglycosylation requires the sequential actions of a defined set of enzymes from numerous glycosyl hydrolase families, unveiling a highly coordinated pathway for Rh1 maturation. Furthermore, we show that in order to trim the final mannose residues (M3, M4, and M5) and the final N-acetylglucosamine residues (N1 and N2) at the base of the N-glycan (Figure 1), Rh1 requires the actions of several lysosomal and plasma membrane enzymes that, to date, have not been characterized within the context of N-glycan processing in the secretory pathway. Therefore, our analysis of Rh1 deglycosylation has uncovered novel roles for these glycosyl hydrolase enzymes during protein biosynthesis. Our findings on the glycosyl hydrolases in *Drosophila* provide insights for future studies aimed at unraveling the complex process of N-glycosylation *in vivo*, as well as for understanding mechanisms that underlie human disease.

## Results

### Identification of mutant alleles affecting N-glycosylation of Rh1

**Figure 3:**
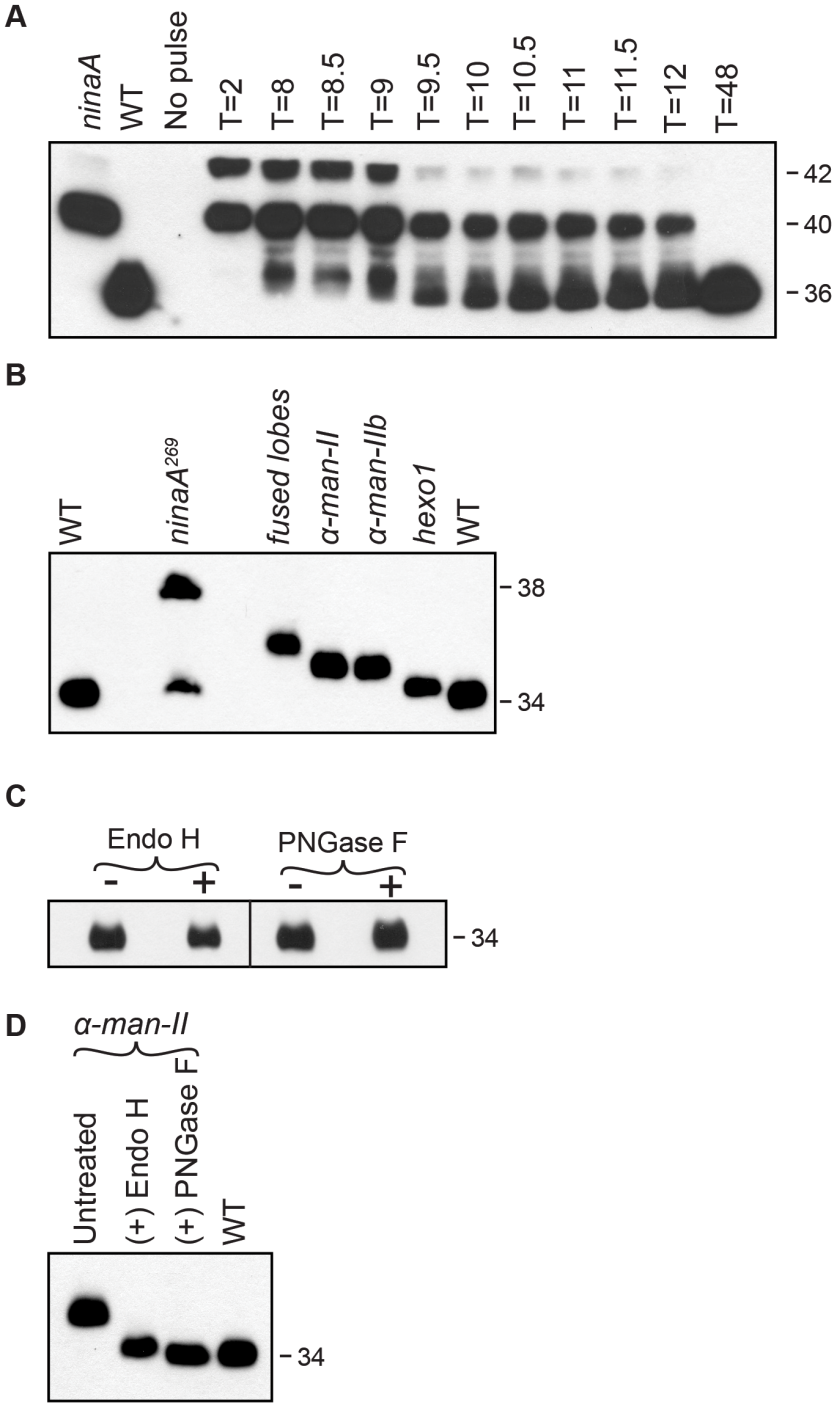
Rh1 is deglycosylated in a step-wise fashion. (**A**) Western blot of Rh1 protein from transgenic flies carrying the *P[hs:Rh1-bov]* construct, probed with an antibody (1D4) directed to the bov epitope tag on Rh1. Following a 1 hour heat pulse at 37°C, flies were shifted to 22°C and assayed at the indicated times (T, in hours). Lanes 1 and 2 show Rh1-bov under the control of the endogenous Rh1 promoter, expressed in either *ninaA^269^* mutant or wild-type (WT) flies. The epitope tag adds ∼2 kD to Rh1, such that the immature forms are detected at 42 and 40 kD and the mature form is detected at 36 kD. Five fly heads were loaded per lane, with the exception of lane 2 (WT), in which 1/6 head was loaded. (**B**) Western blot of Rh1 protein from wild-type (WT), *ninaA^269^*, *fdl^d03111^*, *α-man-II^LL01094^*, *α-man-IIb^f02524^*, and *hexo1^e00001^* mutant flies. One half of a fly head was loaded per lane, with the exception of lane 2 (*ninaA*), in which 15 heads were loaded per lane. (**C**) Western blot of Rh1 protein from Canton S. wild-type tissue, treated (+) with either Endo H or PNGase F enzyme. (**D**) Western blot of Rh1 protein from *α-man-II^LL01094^* mutant tissue (Lane 1), treated (+) with either Endo H (Lane 2) or PNGase F (Lane 3), and compared alongside a 1-head wild-type sample (Lane 4, WT).

Using heat pulse-chase experiments, we have shown that Rh1 is initially synthesized in high-molecular weight (MW) glycosylated forms, that are processed down to the mature form (Figure 3A) [40]. Therefore, unlike all other known glycoproteins, the N-glycan on Rh1 is trimmed but *not* subsequently built upon to form larger carbohydrate structures [36]. Because of this highly unique characteristic, Rh1 offers a powerful model for studying the glycosyl hydrolases without their action being obscured by subsequent carbohydrate addition.

To identify genetic loci critical for Rh1 deglycosylation during biosynthesis, we analyzed flies from the Zuker collection of EMS-generated alleles [41] for defects in carbohydrate trimming by monitoring the molecular weight (MW) of Rh1. In wild-type adult flies, Rh1 is detected in a mature 34 kD form (Figure 3B). Mutations that result in defective trimming of the oligosaccharide chain during Rh1 biosynthesis lead to the accumulation of Rh1 in abnormal high MW forms. For example, NinaA is a chaperone required for the exit of Rh1 from the ER [39], [42]–[44]. Accordingly, in *ninaA^269^* mutants, Rh1 is detected in an immature, 38 kD hyperglycosylated form (Figure 3B) [39], [45].

In our EMS screen, we observed defects in Rh1 deglycosylation in two mutant alleles and performed deficiency mapping, followed by sequence analysis, to identify each mutant locus. We identified a point mutation that leads to a G572E substitution in CG4606, which encodes a Class II α-mannosidase enzyme from GH Family 38, termed α-mannosidase-IIb (α-Man-IIb). We also identified a substitution leading to a pre-mature stop codon (Q592X) in CG1318, which encodes a β-N-acetylglucosaminidase enzyme from GH Family 20, termed hexosaminidase 1 (Hexo1).

Identification of the *α-man-IIb^G572E^* and *hexo1^Q592X^* mutants served as the starting point for a larger analysis of the role of glycosyl hydrolases from these GH families in Rh1 deglycosylation. Specifically, we focused on Class II α-mannosidases from GH Family 38 [46] and hexosaminidases from GH Family 20 [47] with potential roles in N-glycosylation. We identified additional mutant alleles of *α-man-IIb* and *hexo1*, as well as mutant alleles corresponding to a second Class II α-mannosidase from GH Family 38, α-mannosidase-II (α-Man-II, CG18802), and a second hexosaminidase from GH Family 20, termed fused lobes (Fdl, CG8824). Here, we characterize the roles of α-Man-II, α-Man-IIb, fused lobes, and Hexo1 during Rh1 deglycosylation *in vivo* (Figure 3B). Again, the *ninaA^269^* mutant is shown for reference, as Rh1 is retained in the ER in this mutant, and thus N-glycan trimming is blocked at a very early stage during biosynthesis. Accordingly, mutations in *ninaA* lead to the accumulation of a hyperglycosylated, immature form of Rh1 that is significantly larger than the mutant forms observed in the glycosyl hydrolase mutants, which function for N-glycan trimming downstream in the Golgi (Figure 3B).

To determine the extent of Rh1 deglycosylation during its biosynthesis, we performed digestions with endoglycosidase H (Endo H) and peptide N-glycosidase F (PNGase F) and found that the mature form of Rh1 is completely insensitive to treatment with either enzyme (Figure 3C). These results suggest that mature Rh1 protein is completely void of carbohydrate. However, it is possible that mature Rh1 is highly trimmed, but retains a small amount of carbohydrate that is not resolved by SDS-PAGE. To determine whether we are able to detect single oligosaccharide differences via Western blot analysis, we took advantage of Rh1's sensitivity to both Endo H and PNGase F in the *α-man-II* mutants. Endo H cleaves between the first and second N-acetyl glucosamine (GlcNAc) residues, N1 and N2, leaving N1 attached to the protein (Figure 1). In contrast, PNGase F cleaves between N1 and the asparagine residue on Rh1, thus removing the final GlcNAc residue (Figure 1). Therefore, following PNGase F digestion, no carbohydrate remains. We compared the MW of Rh1 protein in *α-man-II* mutant tissue that had been treated with each enzyme. Here, we show that when *α-man-II* mutant fly extracts were treated with Endo H, the Rh1 protein was slightly larger than when treated with PNGase F (Figure 3D). These results demonstrate that we can detect the presence or absence of a single GlcNAc residue. Further, the MW of mature Rh1 protein in wild-type flies is indistinguishable from the Rh1 protein in the PNGase F-treated sample (Figure 3D). These results support the concept that the final GlcNAc residue has been removed from mature Rh1 and are consistent with the hypothesis that Rh1 is fully deglycosylated during biosynthesis.

The results presented here demonstrate that Rh1 deglycosylation is a highly complex process involving defined and sequential trimming steps, carried out by a cascade of glycosyl hydrolase enzymes. In addition to α-Man-II, α-Man-IIb, fused lobes, and Hexo1, we have identified numerous glycosyl hydrolases in *Drosophila* with potential functions in N-glycosylation. The *Drosophila* glycosyl hydrolases that we identified displayed a high degree of amino acid identity with corresponding enzymes in humans, allowing us to place them into well-defined families and subfamilies (Figures 2 and S2, S3, S4, S5, S6, S7). Therefore, in addition to our characterization of α-Man-II, α-Man-IIb, fused lobes, and Hexo1, we provide an informatics-based analysis of the full complement of human and *Drosophila* glycosyl hydrolases involved in N-glycan processing (see Supporting Information and Figure 2).

### Early steps in N-glycan processing

**Figure 4:**
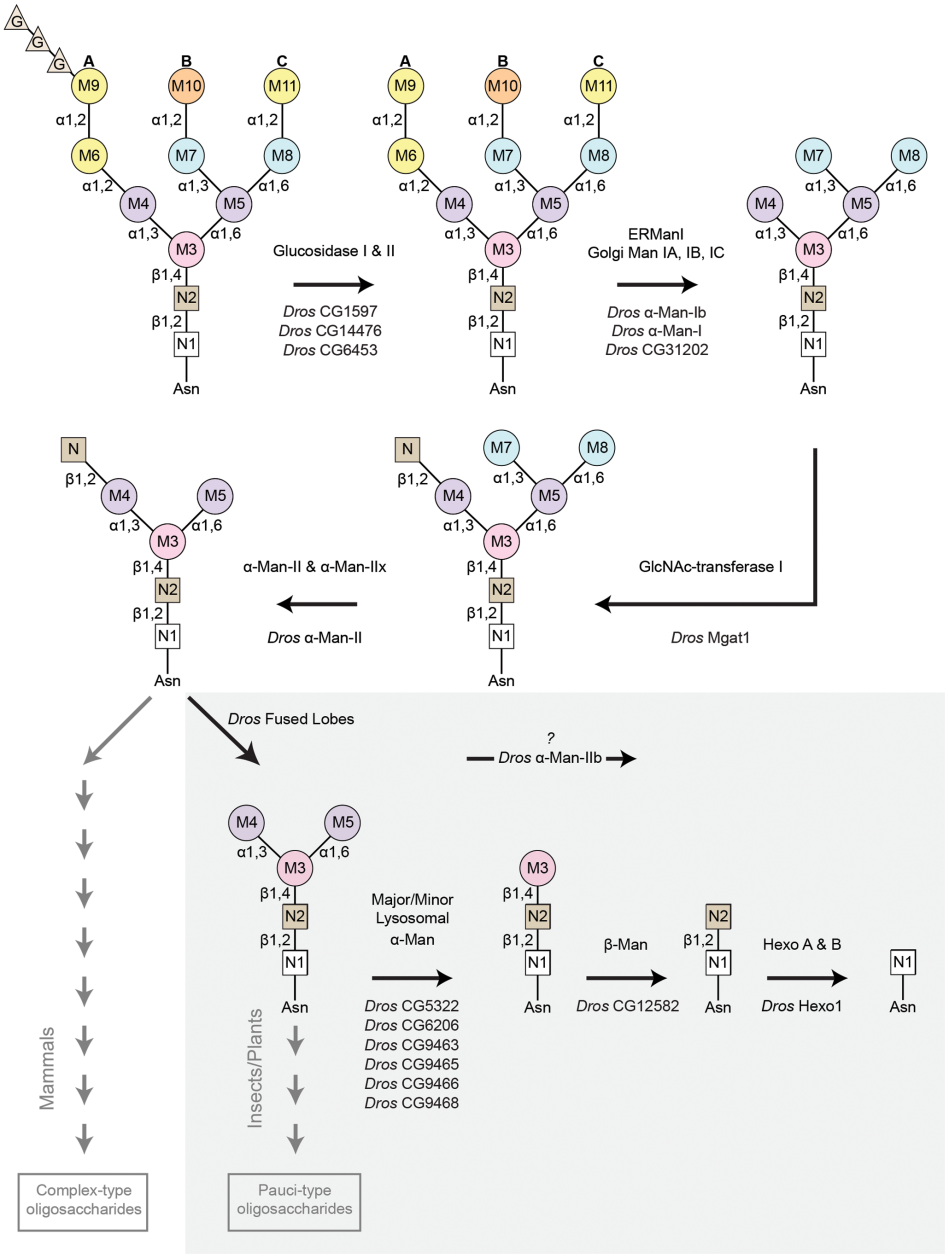
Pathway for Rh1 oligosaccharide trimming. Rh1 deglycosylation is a highly coordinated pathway that requires the sequential actions of a large number of glycosyl hydrolase (GH) enzymes and families. Here, we illustrate the major steps that are thought to occur during Rh1 deglycosylation, beginning with the precursor oligosaccharide, Glc_3_Man_9_GlcNAc_2_. The enzymes responsible for equivalent hydrolysis reactions in mammals are shown above each arrow. The *Drosophila* enzymes predicted to perform these steps during Rh1 maturation are indicated below each arrow. Of particular note, the step mediated by *Drosophila* fused lobes is unique to insects and plants. Removal of this terminal GlcNAc residue has not been shown to occur in mammals. This explains why elongation of human glycoproteins leads to the formation of complex-type oligosaccharides, whereas elongation of *Drosophila* glycoproteins leads to the formation of pauci-mannosidic structures. Importantly, the subsequent steps contained within the grey box represent a novel pathway for Rh1 maturation. That is, the mammalian enzymes responsible for these cleavage events have only been characterized in the context of carbohydrate catabolism in the lysosome, but have no known roles in glycoprotein processing. Here, we propose that the corresponding enzymes in *Drosophila* play a critical role in Rh1 deglycosylation during biosynthesis, leading to the complete deglycosylation of Rh1. G (glucose), M (mannose), N (β-*N*-acetylglucosamine, GlcNAc), Man (mannosidase), Hexo (hexosaminidase).

N-glycan processing begins with the sequential removal of three terminal glucose residues from branch A by α-glucosidase I and α-glucosidase II (Figures 1, 2 and 4) [48]. Following glucose removal, a family of ER and Golgi α1,2-mannosidases collectively work to cleave the four α1,2-linked mannose residues from the N-glycan structure to yield Man_5_GlcNAc_2_ (Figures 1 and 4) [49]. These enzymes belong to GH Family 47 and are also referred to as Class I α1,2-mannosidases (Figure 2) [50]. Following trimming to Man_5_GlcNAc_2_, the classical pathway for oligosaccharide maturation involves the action of GlcNAc-transferase I, which functions to add a single β1,2-linked N-acetylglucosamine (GlcNAc) to the terminal mannose (M4) on branch A (Figures 1, 2 and 4) [51], [52]. The presence of the terminal GlcNAc residue serves as a signal for additional trimming events mediated by a group of Class II α-mannosidases belonging to GH Family 38 (Figure 2) [35], [51], [53]. Class II α-mannosidases have complementary specificity compared to the Class I enzymes from GH Family 47, primarily cleaving α1,3- and α1,6-linked mannose residues from the N-glycan structure [54], as opposed to α1,2-linked mannose residues (Figure 1). We have identified *Drosophila* homologs for each of the components in this pathway (Figures 2 and 4), and provide detailed amino acid (aa) alignments and analyses in Supporting Figures S2–S5.

### *Drosophila* α-Man-II functions upstream from α-Man-IIb for Rh1 maturation

The presence of the terminal GlcNAc residue, added by GlcNAc-transferase I, serves as a signal for additional trimming events mediated by a group of Class II α-mannosidases belonging to GH Family 38 (Figure 4) [35], [51], [53]. The first trimming events mediated by the Class II enzymes involve the sequential removal of two mannose residues, M7 and M8 (Figure 1), from the GlcNAc_1_Man_5_GlcNAc_2_ structure [35]. Removal of these residues represents the “committed” step in N-glycan processing and is responsible for the conversion from high-mannose, Endo H-sensitive structures, to complex-type mannose structures that are insensitive to Endo H [3], [55]. This step occurs in the Golgi and is mediated by a pair of isozymes from GH Family 38 (Subgroup A) termed α-mannosidase II (α-Man II) and α-mannosidase IIx (α-Man IIx) in mammals [56]. These enzymes are encoded by MAN2A1 [57] and MAN2A2 [58] in humans, respectively (Figure 2). We have identified two GH Family 38 members in *Drosophila* from Subgroup A, namely α-mannosidase-II (α-Man-II, CG18802) and α-mannosidase-IIb (α-Man-IIb, CG4606) (Figure 2).

The evolutionary history between the human and *Drosophila* enzymes may yield insight into their potential functions. The mammalian enzymes, α-Man-II and α-Man-IIx, arose from a fairly recent gene duplication event (Figure S5A) and are isozymes, with identical substrate specificities. Therefore, these enzymes have yet to undergo functional specialization. They have, however, developed tissue-specific expression patterns [57], [59]. Based on aa identity, *Drosophila* α-Man-II appears to represent the ortholog of these isozymes (Figure S5A). *Drosophila* α-Man-II displays 40% overall aa identity with both human Man II and Man IIx, and 56–57% aa identity within the GH Family 38 N-terminal catalytic domain (Figure S5B). By comparison, *Drosophila* α-Man-IIb is significantly more divergent (Figure S5A), displaying only 33–34% overall aa identity with the human enzymes and only 32–46% aa identity within the GH Family 38 N-terminal catalytic domain (Figure S5B). It is therefore possible that *Drosophila* α-Man-IIb has evolved a specialized function, unique from the other three mannosidases. To investigate this possibility and to further understand the role of *Drosophila* α-Man-II and α-Man-IIb in Rh1 biosynthesis, we assessed defects in Rh1 maturation in mutant alleles corresponding to these loci.

**Figure 5:**
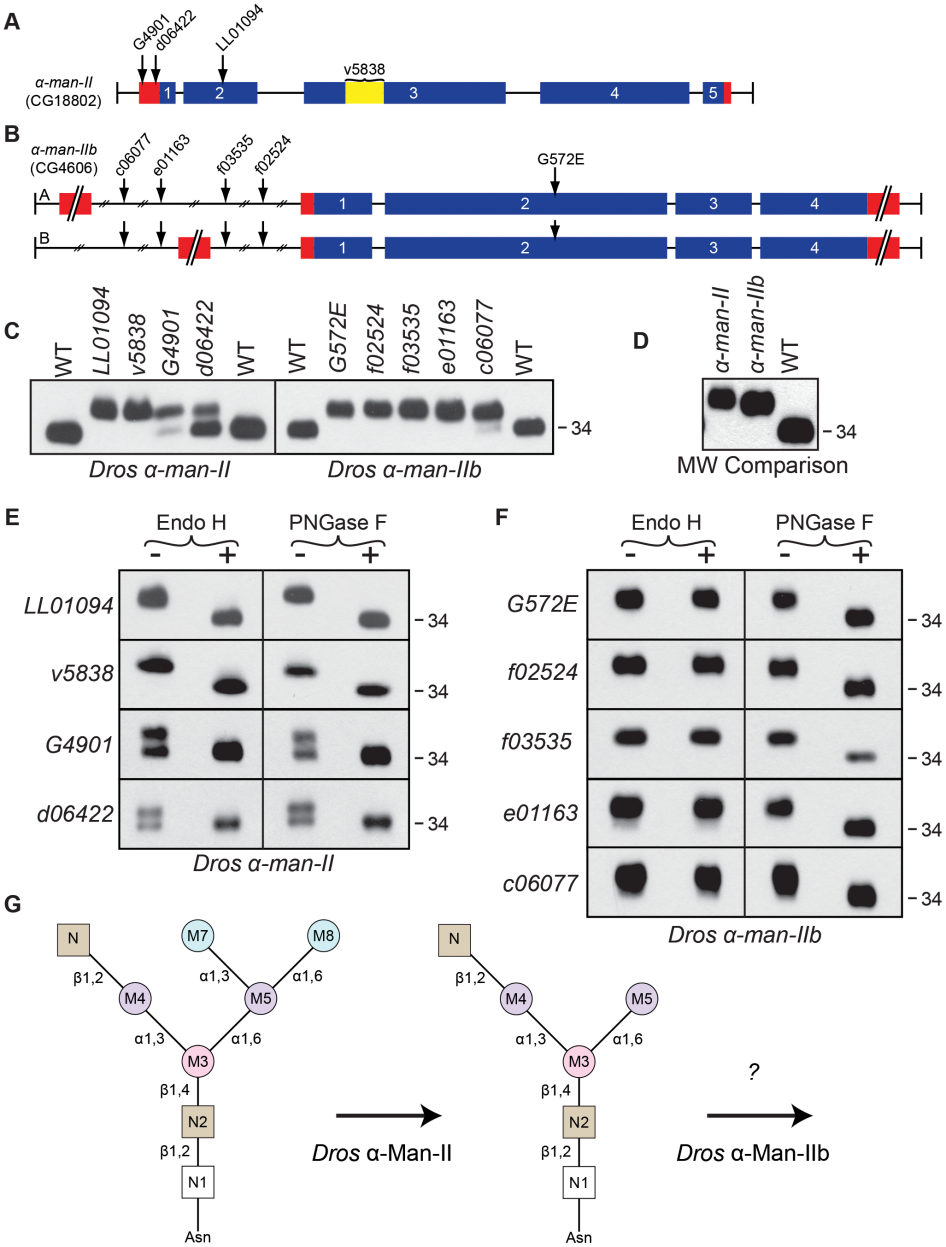
*Drosophila* α-Man-II functions upstream from α-Man-IIb during Rh1 deglycosylation. Intron/Exon structures for (**A**) *α-mannosidase-II* (*α-man-II*, CG18802) and (**B**) *α-mannosidase-IIb* (*α-man-IIb*, CG4606) are shown to indicate the alleles used in this study: *P[EP]α-man-II^G4901^*, *P[XP]α-man-II^d06422^*, *Pbac[SAstopDsRed]α-man-II^LL01094^*, *P[GD2875]^v5838^*, *Pbac[PB]α-man-IIb^c06077^*, *Pbac[RB]α-man-IIb^e01163^*, *Pbac[WH]α-man-IIb^f03535^*, *Pbac[WH]α-man-IIb^f02524^*, and *α-man-IIb^G572E^*. Blue = coding sequence, red = additional mRNA, yellow = RNAi target. (**C**) Western blots of Rh1 protein from *α-man-II* (Left) and *α-man-IIb* (Right) mutant flies. Left Lanes: (1) Wild-type (WT), (2) *α-man-II^LL01094^*, (3) *α-man-II^v5838^*, (4) *α-man-II^G4901^*, (5) *α-man-II^d06422^*, and (6) WT. Right Lanes: (1) WT, (2) *α-man-IIb^G572E^*, (3) *α-man-IIb^f02524^*, (4) *α-man-IIb^f03535^*, (5) *α-man-IIb^e01163^*, (6) *α-man-IIb^c06077^*, and (7) WT. One head was loaded per lane, with the exception of lane 3 on the left side (*α-man-II^v5838^*), in which 4 heads were loaded. (**D**) Western blot, comparing the molecular weight of Rh1 in the *α-man-II^LL01094^* and *α-man-IIb^f02524^* mutants, indicating that Rh1 is slightly larger in *α-man-II* mutants. One half of a head was loaded per lane. (**E and F**) Western blot of Rh1 protein from the *α-man-II* and *α-man-IIb* alleles described in (C), treated (+) with either Endo H or PNGase F enzyme and labeled for Rh1. (**G**) Proposed role for *Drosophila* α-Man-II in trimming the M7 and M8 mannose residues (blue) during Rh1 biosynthesis. Our data indicate that *Drosophila* α-Man-IIb functions downstream at a step that is distinct from α-Man-II during Rh1 deglycosylation.

Mutations in *Drosophila α-man-II* are homozygous lethal. Therefore, we employed a variety of techniques to obtain viable mutant alleles. First, we generated mosaic clones of a homozygous lethal P-element allele, *α-man-lI^LL01094^*, in the eye, using a FLP/FRT recombination method [60]. Second, we utilized tissue-targeted RNA interference (RNAi) to reduce *α-man-lI* transcript levels, specifically in the eye, using the *α-man-lI^v5838^* UAS-RNAi construct and an eye-specific GAL4 driver line [61]. Finally, we obtained two hypomorphic P-element alleles, *α-man-lI^G4901^* and *α-man-lI^d06422^*, whose insertional sites lie in non-coding regions rendering them homozygous viable. All four alleles are depicted in Figure 5A. Mutations in *α-man-II* lead to defects in the maturation of Rh1 protein, as indicated by the accumulation of a high MW form of Rh1 in all four mutant alleles (Figure 5C, left). The mosaic allele (*α-man-lI^LL01094^*) and RNAi allele (*α-man-lI^v5838^*) are strong alleles as Rh1 is present exclusively in a high MW form, with no mature Rh1 detected (Figure 5C, left). In contrast, some Rh1 protein is present in the mature, wild-type form in the hypomorphic P-element mutants (*α-man-lI^G4901^* and *α-man-lI^d06422^*) (Figure 5C, left). These results indicate that α-Man-II is required for proper deglycosylation of Rh1.

For the *α-man-IIb* locus, we obtained a single EMS-generated allele, *α-man-IIb^G572E^*, and four P-element alleles, *α-man-IIb^f02524^*, *α-man-IIb^f03535^*, *α-man-IIb^e01163^*, and *α-man-IIb^c06077^*, all of which are depicted in Figure 5B. As with *α-man-II*, mutations in *α-man-IIb* lead to defects in Rh1 maturation, as indicated by the accumulation of a high MW form of Rh1 in all five mutant alleles (Figure 5C, right). In the strongest alleles, Rh1 is present exclusively in a high MW form, whereas in others a small amount of Rh1 is detected in the wild-type form (Figure 5C, right), indicating that these weaker mutations are hypomorphic. Indeed, certain alleles are temperature-sensitive and these data are shown in Figure S1. These results indicate that α-Man-IIb is also required for proper deglycosylation of Rh1.

The phenotypes observed in the strongest alleles of both *α-man-II* and *α-man-IIb* show that Rh1 is present exclusively in a high MW form, with no mature Rh1 detected (Figure 5C). These results suggest that, unlike the human α-Man II and α-Man IIx isozymes, *Drosophila* α-Man-II and α-Man-IIb do *not* perform redundant functions, but rather are uniquely required for distinct steps in N-glycan processing during Rh1 maturation. The lethality of *α-man-II* mutants also supports this hypothesis. Still additional support for their independent roles during Rh1 biosynthesis comes from a close comparison in the MW of Rh1 detected in *α-man-II* versus *α-man-IIb* mutants. Specifically, the high MW form of Rh1 in *α-man-II* mutants is slightly larger than the high MW form observed in *α-man-IIb* mutants (Figure 5D) [11], indicating that further trimming has occurred in *α-man-IIb* mutants. These data support the hypothesis that *Drosophila* α-Man-II functions upstream from α-Man-IIb for carbohydrate trimming during Rh1 biosynthesis.

The hypothesis that α-Man-II functions upstream from α-Man-IIb is further supported by the results of Endo H and PNGase F digestions on the mutant tissues. In all four alleles of *α-man-II* and all five alleles of *α-man-IIb*, the high MW form of Rh1 was sensitive to digestion with PNGase F, demonstrating that the abnormal, high MW Rh1 protein was indeed hyperglycosylated (Figures 5E and 5F). The *α-man-II* mutants were also sensitive to digestion with Endo H (Figure 5E), indicating that the M7 and M8 mannose residues had not yet been removed from the N-glycan. These findings are consistent with the predicted role for α-Man-II in the removal of M7 and M8. This is also the function of the mammalian isozymes, α-Man II and α-Man IIx [56]. In contrast, the *α-man-IIb* mutants were insensitive to treatment with Endo H (Figure 5F), indicating that the M7 and M8 residues had already been removed and that Rh1 had progressed in the Golgi past this step. Taken together, these data strongly support the notion that α-Man-IIb functions downstream from α-Man-II, and downstream from M7 and M8 removal, for Rh1 deglycosylation during biosynthesis. Again, the finding that Rh1 is detected exclusively in a high MW form in strong alleles of *α-man-II* and *α-man-IIb* indicates that, not only do they function at unique steps during Rh1 deglycosylation, but also that there is no functional redundancy between the two *Drosophila* enzymes. These results are consistent with the highly divergent amino acid sequence of *Drosophila* α-Man-IIb, compared with the other members of GH Family 38, Subgroup A (Figure S5). Although our results clearly indicate a role for α-Man-IIb in Rh1 deglycosylation, downstream from α-Man-II and removal of M7 and M8, the specific role of this enzyme in trimming is not yet known (Figure 5G). These data are consistent with a possible role for α-Man-IIb in trimming M5.

### *Drosophila* fused lobes is essential for Rh1 deglycosylation

Following removal of the M7 and M8 residues, the N-glycan trimannosyl core is built upon to form a variety of N-glycan structures. In mammals, the N-glycan typically retains the terminal GlcNAc residue (attached to M4) and is additionally modified with fucosyl, galatosyl, and/or sialyl residues to form complex-type N-glycan structures in the Golgi (Figure 4) [62], [63]. In contrast, insect cells hydrolyze this terminal GlcNAc by the action of a β-N-acetylhexosaminidase, leading to the generation of paucimannosidic N-glycan structures (Figure 4) [2], [64]. The hexosaminidase responsible for this step in *Drosophila*, termed fused lobes (CG8824), is a member of GH Family 20 [10]. This gene was originally named due to the mushroom body *fused lobes* phenotype in the corresponding mutant [65]. Interestingly, mammalian β-hexosamindases from GH Family 20 are generally present as soluble proteins in the lysosome and have well-characterized roles in the catabolism and recycling of sugar residues. Unique among the β-hexosaminidases, *Drosophila* fused lobes is a membrane-associated protein that has been implicated as a processing enzyme responsible for removing the terminal GlcNAc residue attached to the M4 residue on nascent glycoproteins (Figure 4). This event was proposed to be a routine step during glycoprotein maturation in the secretory pathway [10].

**Figure 6:**
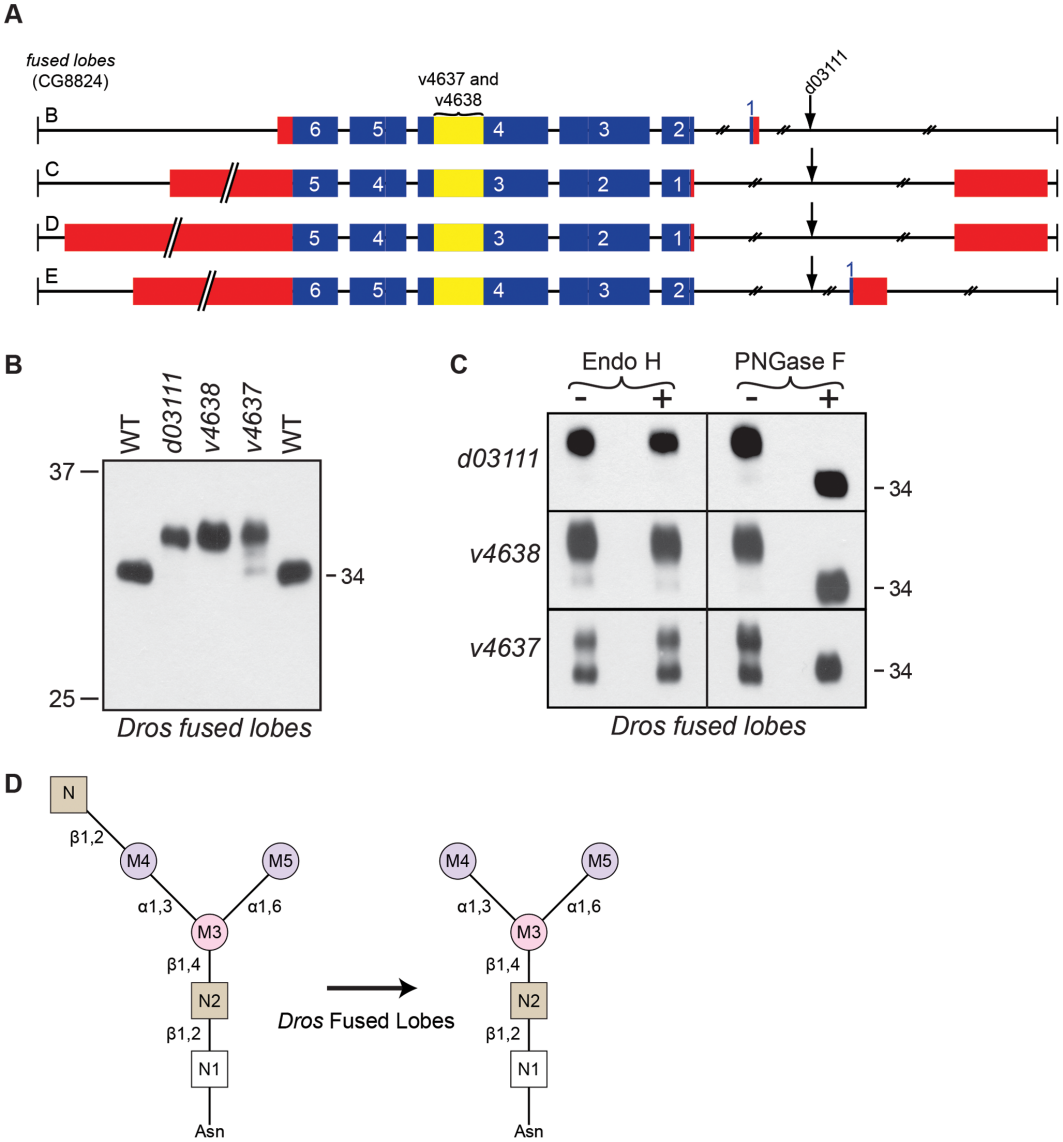
*Drosophila* Fdl is essential for Rh1 deglycosylation. (**A**) Intron/Exon structure for *fused lobes* (*fdl*, CG8824), indicating the alleles used in this study: *P[GD1956]^v4637^*, *P[GD1956]^v4638^*, and *P[XP]fdl^d03111^*. Blue = coding sequence, red = additional mRNA, yellow = RNAi target. (**B**) Western blot of Rh1 protein from *fused lobes* mutants. Lanes: (1) Wild-type (WT), (2) *fdl^d03111^*, (3) *fdl^v4638^*, (4) *fdl^v4637^*, and (5) WT. One half of a head was loaded in Lanes 1, 2, and 5, whereas 4 heads were loaded in Lanes 3 and 4. (**C**) Western blot of Rh1 protein from the *fused lobes* alleles described in (B), treated (+) with either Endo H or PNGase F enzyme. (**D**) Proposed role for *Drosophila* fused lobes in trimming the terminal GlcNAc residues attached to M4 during Rh1 biosynthesis.

To investigate the role of fused lobes in Rh1 deglycosylation, we obtained a single P-element allele, *fdl^d03111^*, as well as two RNAi alleles, *fdl^v4637^* and *fdl^v4638^*, all of which are shown in Figure 6A. In all three *fused lobes* mutants, Rh1 was detected in an abnormal high MW form (Figure 6B). These results indicate that fused lobes is required for the proper maturation of Rh1 during biosynthesis. The high MW form of Rh1 detected in *fused lobes* mutants was sensitive to digestion with PNGase F, indicating that the protein was hyperglycosylated (Figure 6C). In contrast, Rh1 was insensitive to treatment with Endo H in the *fused lobes* mutants, indicating that M7 and M8 had been removed from the N-glycan in the Golgi (Figure 6C). These results are consistent with the predicted role of fused lobes, downstream from α-Man-II (and removal of M7 and M8), in the Golgi during N-glycan trimming.

Surprisingly, the MW of Rh1 in *fused lobes* mutants was larger than the MW observed in *α-man-II* mutants (Figure 3B). Given that fused lobes functions downstream from α-Man-II, these data suggest that the N-glycan not only retains its terminal GlcNAc residue in the absence of fused lobes, but must also undergo some form of elongation. Elongation or elaboration of the N-glycan, triggered by the abnormal retention of GlcNAc, would account for the larger size of Rh1 in *fused lobes* mutants. Our data are consistent with previous findings that mutations in *Drosophila fused lobes* lead to the abnormal generation of hybrid- and complex-type glycans that are more typically seen in vertebrates [10]. Therefore, mutations in *fused lobes* may promote the formation of a complex N-glycan structure on Rh1.

### Trimming the trimannosyl core during Rh1 deglycosylation

Whether the terminal GlcNAc residue is removed (as it is in plants and insects) or retained (as it is in mammals), the subsequent stages in N-glycosylation almost always involve elongation and elaboration of the N-glycan structure by the addition of diverse sugar moieties (Figure 4). In stark contrast, the N-glycan on *Drosophila* Rh1 is further trimmed during biosynthesis (Figure 3A) [36]–[39]. Indeed, our data are consistent with the hypothesis that the mature form of Rh1 is completely void of carbohydrate (Figures 3C and 3D). Step-wise reduction of the remaining trimannosyl core would theoretically involve removal of the residual α-linked mannose residues, M4 and M5, the β-linked mannose, M3, and the two GlcNAc residues at the base of the N-glycan structure, N1 and N2 (Figure 1). Glycosyl hydrolase enzymes capable of performing these steps have been identified, but they are generally present as soluble proteins in the lysosome.

Specifically, there are two lysosomal α-mannosidases from GH Family 38 Subgroup B: the major lysosomal α-mannosidase (MAN2B1) [66]–[68] and the minor lysosomal α-mannosidase (MAN2B2) (Figure 2) [69]–[71]. These enzymes are required for removal of M4 and M5, respectively, during catabolism of the N-glycan (Figure 1). There is also a lysosomal β-mannosidase from GH Family 2 (Figure 2) that is involved in the removal of the β-linked M3 residue from N-glycan structures during catabolism (Figure 1) [31], [72], [73]. Finally, at the very base of the N-glycan structure lie two GlcNAc residues, N1 and N2 (Figure 1). In mammals, the bond between these residues is cleaved by a pair of β-hexosaminidase enzymes from GH Family 20, Hex A and Hex B [32], [74]–[76]. Again, this particular reduction occurs in the context of N-glycan catabolism and the recycling of sugar residues in the lysosome. We have identified *Drosophila* homologs for each of the components in this catabolic pathway (Figures 2 and 4), and provide detailed amino acid (aa) alignments and analyses in Supporting Figures S5–S7.

The finding that none of these lysosomal enzymes had predicted functions in the biosynthetic processing of glycoproteins presented a conundrum for the case of Rh1 deglycosylation, given that the trimannosyl core structure is removed during Rh1 maturation. One possible explanation is that the residual trimannosyl core is *not* removed in a step-wise fashion by exo-acting enzymes, but rather an endo-acting hydrolase removes the remaining structure from Rh1 in bulk. If this were the case, the lysosomal enzymes would not be required for Rh1 deglycosylation. Another possible explanation is that some of the enzymes previously characterized as ‘lysosomal’, have in fact evolved functions in glycoprotein processing and are involved in the step-wise deglycosylation of Rh1. Indeed, non-traditional roles have been assigned to a number of enzymes formerly thought to function solely in the lysosome. For example, a number of ‘lysosomal’ enzymes have been shown to localize to the sperm plasma membrane and facilitate sperm-egg interaction during fertilization [77]. Here, we present evidence that one such enzyme in *Drosophila*, Hexo1, plays a role in Rh1 biosynthesis in the secretory pathway. Our results lend support to the hypothesis that, indeed, Rh1 is deglycosylated, residue by residue, until the protein is completely void of carbohydrate.

### *Drosophila* Hexo1 is uniquely required for Rh1 deglycosylation

In humans, there are two loci encoding β-*N*-acetylhexosaminidase enzymes from GH Family 20, termed HEXA and HEXB (Figure 2). The Hex A enzyme is a heterodimer (αβ) containing one α-subunit encoded by the HEXA locus [75] and one β-subunit encoded by the HEXB locus [76]. In contrast, the Hex B enzyme is a homodimer of β-subunits (ββ), and thus only requires the HEXB gene [32]. These enzymes have been extensively investigated, due to their known roles in Sandhoff and Tay-Sachs disease, and function to remove terminal GlcNAc or GalNAc residues from a variety of carbohydrate and glycolipid structures. We have identified three hexosaminidases belonging to GH Family 20 in *Drosophila*, namely hexosaminidase 1 (Hexo1, CG1318), hexosaminidase 2 (Hexo2, CG1787), and a third hexosaminidase characterized above, fused lobes (Fdl, CG8824) (Figure 2). Although β-hexosamindases from GH Family 20 are generally thought of as lysosomal enzymes, involved in the recycling of sugar residues, numerous non-traditional roles have been identified for these ‘catabolic’ enzymes. Both the human and *Drosophila* hexosaminidases have been identified on the plasma membrane of various cell types [47], [78] and, of particular interest, human Hex B has been shown to localize to the sperm acrosome, which is derived from the Golgi apparatus [79]. Given these uncharacteristic qualities, and the known role of fused lobes in glycoprotein processing, we were interested in the potential role of *Drosophila* Hexo1 and Hexo2 in cleaving the final GlcNAc residues on Rh1 in the secretory pathway.

**Figure 7:**
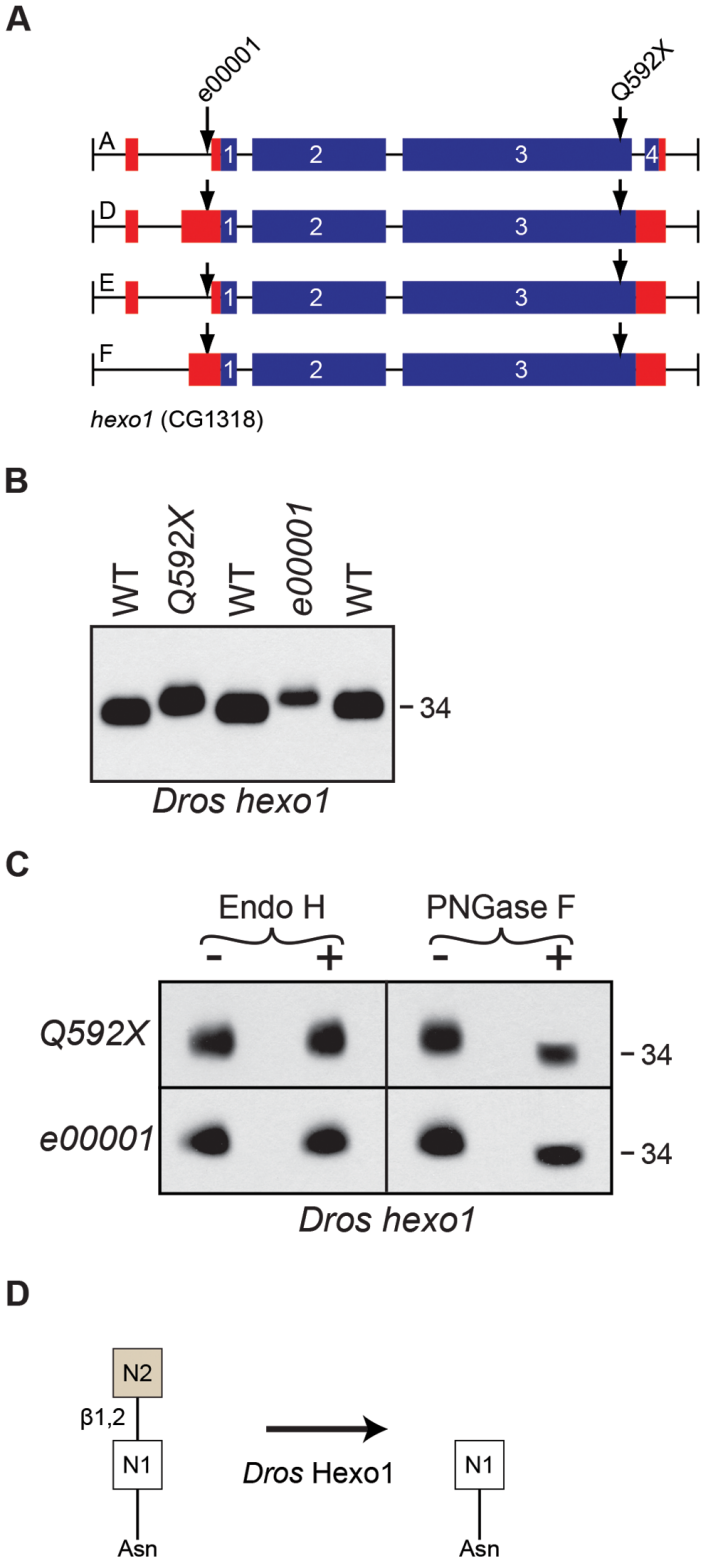
*Drosophila* Hexo1 is essential for Rh1 deglycosylation. (**A**) Intron/Exon structures for *hexosaminidase1* (*hexo1*, CG1318), indicating the alleles used in this study: *Pbac[RB]hexo1^e00001^* and *hexo1^Q592X^*. Blue = coding sequence, red = additional mRNA. (**B**) Western blot of Rh1 protein from *hexo1* mutant flies. Lanes: (1) WT, (2), *hexo1^Q592X^* ^/Df(3L)ED4341^, (3) *hexo1^e00001^*, and (4) WT. Deficiency Df(3L)ED4341 deletes cytological region 63F6-64B9 and thus fails to complement the *hexo1* locus, which lies at 64A12. 2 heads were loaded per lane. (**C**) Western blot of Rh1 protein from the *hexo1* alleles described in (B), treated (+) with either Endo H or PNGase F enzyme. (**D**) Proposed role for *Drosophila* Hexo1 in trimming the N2 GlcNAc residue from the N-glycan during Rh1 biosynthesis.

For the *hexo1* locus, we identified an EMS-generated allele, *hexo1^Q592X^*, as well as a P-element allele, *hexo1^e00001^*, both of which are depicted in Figure 7A. Mutations in *hexo1* lead to defects in Rh1 deglycosylation, as indicated by the accumulation of Rh1 in a slightly higher MW form in both *hexo1* mutants (Figure 7B). The very subtle difference in size between the high MW form of Rh1 observed in *hexo1* mutants and the wild-type form of Rh1 is consistent with a defect in removing a GlcNAc residue at the very end of the deglycosylation cascade. This high MW form of Rh1 is sensitive to digestion with PNGase F, but insensitive to digestion with Endo H (Figure 7C). These results indicate that the hyperglycosylated Rh1 protein detected in *hexo1* mutants has already been processed by the Class II α-mannosidases in the Golgi. Taken together, these results are consistent with a role for Hexo1 in the removal of the N2 GlcNAc residue from the base of the N-glycan during Rh1 biosynthesis (Figure 7D).

Analysis of two *hexo2* P-element alleles, *hexo2^f04129^* and *hexo2^d06829^*, did not reveal defects in the MW of Rh1 protein (data not shown). This does not rule out a potential role for Hexo2 in Rh1 deglycosylation, as there could be other enzymes with redundant functions. However, the finding that Rh1 is present exclusively in a high MW form in both the *fused lobes* (Figure 6B) and *hexo1* (Figure 7B) mutants, and that these two MWs are clearly distinct from one another (Figure 3B), indicates that both fused lobes and Hexo1 are independently required for unique steps in Rh1 deglycosylation.

### Summary: Rh1 is deglycosylated in a step-wise fashion

Here, we demonstrate that α-Man-II, α-Man-IIb, fused lobes, and Hexo1, all play essential and unique roles during Rh1 maturation. Although three of these enzymes have known roles in N-glycoprotein processing, Hexo1 has not been previously shown to be involved in protein biosynthesis. Our results demonstrate that Hexo1 plays a critical role during Rh1 deglycosylation in the secretory pathway and provide direct evidence that the N-linked oligosaccharide on Rh1 is removed in a step-wise fashion, down to the final GlcNAc residue (Figure 4). Our findings, demonstrating step-wise trimming of the final trimannosyl core residues during Rh1 biosynthesis reveal exciting new possibilities for the complex functions of glycosyl hydrolases in the secretory pathway.

## Discussion

It has been known for over 25 years that Rh1 is deglycosylated during biosynthesis [36], however much of the deglycosylation pathway for Rh1 has remained unknown. In this same timeframe, an enormous body of research has led to the identification and characterization of a wide variety of glycosyl hydrolase enzymes involved in N-glycan processing. These studies have focused predominantly on the biochemical characterization of mammalian glycosyl hydrolases in cell culture. *Drosophila* remains a highly effective genetic model for the dissection of biological pathways and has the potential to yield enormous insight into the functions of N-glycans *in vivo*. To date, a comprehensive informatics comparison between human and *Drosophila* glycosyl hydrolases involved in N-glycan processing has not been presented. Here, we present a framework for such a comparison between the human and *Drosophila* glycosyl hydrolase families (Figures 2 and S2–S7) and describe a role for *Drosophila* glycosyl hydrolases in the sequential trimming of the N-glycan during Rh1 biosynthesis. We characterize mutations in four critical glycosyl hydrolase enzymes, α-Man-II, α-Man-IIb, fused lobes, and Hexo1, and unveil a highly coordinated pathway for Rh1 deglycosylation in *Drosophila* photoreceptor cells (Figure 4).

Whereas α-Man-II, α-Man-IIb, and fused lobes have previously been implicated in N-glycoprotein processing, Hexo1 has not, and therefore our findings on the role of Hexo1 in the secretory pathway are particularly intriguing. Although hexosaminidases are typically thought of as lysosomal enzymes involved in N-glycan catabolism, both human and *Drosophila* hexosaminidases have been previously identified outside of the lysosome. For example, Hex A has been identified on the plasma membrane of human fibroblasts [80] and Hex B has been shown to localize to the sperm acrosome, which is derived from the Golgi apparatus [79]. All three *Drosophila* hexosaminidases have been identified on the sperm plasma membrane and are thought to mediate egg-sperm binding by forming complexes with oligosaccharide substrates present on the egg zona pellucida (ZP) [47], [78]. Interestingly, it has been suggested that the family of β-hexosaminidases may be posttranslationally and differentially processed in different tissue types, resulting in either a membrane-bound form of the enzyme (typically on the plasma membrane) or a soluble form that is either localized to the cytosol of the lysosome or secreted from the cell [47]. Given this apparent flexibility in cellular localization, and the non-traditional roles that these ‘lysosomal’ enzymes have already been shown to play, a role for Hexo1 during Rh1 maturation in the secretory pathway is not unprecedented.

The predicted evolutionary relationship between the human and *Drosophila* genes from GH Family 20 provides further insight into the potentially broad functions of hexosaminidases. Based on amino acid analysis, the phylogenetic tree shown in Figure S6A predicts a single hexosaminidase in the last common ancestor between humans and *Drosophila*. Therefore, the human gene pair and the *Drosophila* gene triplet have evolved separately, through independent duplication events. As is always the case, gene duplication provides the evolutionary potential for functional specialization and it is very likely that the *Drosophila* loci have evolved unique and/or additional functions compared to the human loci. This has already been shown for the fused lobes hexosaminidase from this gene family. Finally, as with the HEXA and HEXB loci in humans, the three *Drosophila* loci from GH Family 20 (*fdl*, *hexo1*, and *hexo2*) are thought to encode subunits that can pair in unique combinations to form dimeric hexosaminidase enzymes [78]. For example, two dimeric hexosaminidase enzymes have been identified on the sperm plasma membrane in *Drosophila*, including HEXA (which consists of one fused lobes subunit and one Hexo2 subunit) and HEXB (which consists of one Hexo1 subunit and one Hexo2 subunit) [47]. Factoring in homo-dimeric combinations, the human gene pair can yield only three hexosaminidase enzymes. In contrast, the three *Drosophil*a loci can encode a total of six unique enzymes. Again, this allows significantly greater freedom for functional specialization of the various enzymes and makes a role for Hexo1 and Fused Lobes in glycoprotein biosynthesis entirely feasible.

Our results, demonstrating that Hexo1 plays a critical role for carbohydrate trimming during Rh1 biosynthesis in the secretory pathway, reveal intriguing new possibilities for the complex roles of glycosyl hydrolases traditionally thought to function as catabolic enzymes in the lysosome. For example, it is possible that the two α-linked mannose residues, M4 and M5, are removed by one (or more) of the six *Drosophila* lysosomal α-mannosidases from GH Family 38 Subgroup B (Figures 2 and S4). The pathway for Rh1 deglycosylation would then require the removal of a β-linked mannose, M3, which points to a role for the *Drosophila* lysosomal β-mannosidase from GH Family 20 (Figures 2 and S6). Given the requirement by Rh1 for these trimming events, which should occur upstream of Hexo1, we hypothesize that, like Hexo1, these ‘catabolic’ enzymes may play roles as processing enzymes during glycoprotein biosynthesis.

Interestingly, the six lysosomal α-mannosidases in *Drosophila* display significant amino acid identity with the major lysosomal α-mannosidase in humans, MAN2B1 (Figures S5A and S5C), having relatively no similarity to the minor lysosomal α-mannosidase in humans, MAN2B2. Importantly, the major lysosomal α-mannosidase has high specificity for cleavage of M4, whereas the minor lysosomal α-mannosidase preferentially cleaves M5 (Figure 1). The complementary actions of these two enzymes, along with their ubiquitous expression, suggest that they function in tandem for N-glycan degradation in mammals. These findings indicate that, if they play a role in Rh1 deglycosylation, the *Drosophila* lysosomal α-mannosidases likely function to cleave M4. Given the absence of a direct *Drosophila* homolog for the mammalian minor lysosomal α-mannosidase (Figure 2), it is difficult to predict which glycosyl hydrolase functions to cleave M5.

Just as there is uncertainty regarding the identity of the enzyme responsible for trimming M5 from the trimannosyl core, there is also uncertainty regarding the role of *Drosophila* α-Man-IIb in the pathway for Rh1 deglycosylation. In this study, we demonstrate that α-Man-II and α-Man-IIb are both required at distinct steps during Rh1 maturation and that α-Man-IIb functions downstream from α-Man-II (Figure 5). It has previously been shown that α-Man-II functions for the sequential removal of two mannose residues, M7 and M8, during N-glycan processing (Figure 5) [11]. In contrast, the role of α-Man-IIb is currently unknown. Interestingly, *Drosophila* α-Man-II, α-Man-IIb, and the six lysosomal α-mannosidases are all Class II α-mannosidases from GH Family 38 (Figure 2). Accordingly, these enzymes all have the capacity to remove α1,3- and α1,6-linked mannose residues from the N-glycan structure, including M4, M5, M7, and M8 (Figure 1). Given the predicted role of the lysosomal α-mannosidases in cleaving M4 and the known role of α-Man-II in cleaving M7 and M8, one possibility is that α-Man-IIb functions to remove M5. While this theoretical pairing addresses two unresolved issues in the pathway, there is currently no direct evidence to support a role for α-Man-IIb in trimming M5.

In recent decades, there has been considerable interest in the fundamental nature of insect glycoprotein processing pathways due to the widespread use of the baculovirus-insect cell expression system [81]. This system is used to produce recombinant mammalian glycoproteins for a variety of different biomedical research applications. The insect system is advantageous, as it produces recombinant glycoproteins more readily than mammalian cell expression systems [82]. A major limitation is that insect-cell-produced glycoproteins have significantly different N-glycan structures than those produced by mammalian cells and are rarely of the complex-type [81], [82]. In part, this is due to the presence of the fused lobes β-*N*-acetylglucosaminidase, which removes a critical GlcNAc residue that serves as a building block for complex-type N-glycans (Figure 4). This event was proposed to be a routine step during glycoprotein maturation in the secretory pathway and, accordingly, some fused lobes expression was detected in the Golgi when expressed in yeast [10].

A Golgi hexosaminidase with a role in N-glycoprotein processing has also been identified in the lepidopteran insect, *Spodoptera frugiperda* (Sf21 and Sf9 cells) [64], [83]. This fused lobes ortholog, Sf-Fdl, has a slightly higher pH optimum of 6.0 [64], [83] compared to *Drosophila* fused lobes, which has a pH optimum of 5.5 [10]. However, *Drosophila* fused lobes has a range of activity through pH 6–7, and therefore fused lobes is active in the pH range typical of secretory pathway compartments, such as the Golgi [84]. When expressed in yeast, fused lobes was also detected on the plasma membrane and in multivesicular bodies [10]. Likewise, fused lobes was detected on the sperm plasma membrane [47]. Interestingly, there is currently no report of fused lobes expression in lysosomes.

Due to widespread interest in improving the baculovirus-insect cell expression system, identification of the fused lobes locus in *Drosophila melanogaster* [10] and the lepidopteran insect, *Spodoptera frugiperda* [83] was of great interest to a broad audience. Here, using Rh1 protein as a substrate, we confirm that *Drosophila* fused lobes is indeed required for glycoprotein biosynthesis *in vivo*. Consistent with a range of activity between pH 6–7 [10], our results demonstrate that fused lobes is capable of functioning in the secretory pathway during Rh1 maturation. These, and future, studies on the function of fused lobes will likely assist in the development of baculovirus-insect cell expression systems capable of producing more mammalian-like recombinant glycoproteins. For example, genetic elimination or inactivation of fused lobes in these cell expression systems might promote the formation of the desired complex carbohydrates on mammalian glycoproteins.

Here, we demonstrate that *Drosophila* Rh1 serves as an excellent reporter protein for studying N-glycosylation. In the present study, we have used the molecular weight of Rh1 protein, and its sensitivity to Endo H and PNGase F, in order to gain insights into the roles of the glycosyl hydrolases in the N-glycan trimming cascade. While our data are consistent with the hypothesis that the biochemical activities of these enzymes are the same as they are in vertebrates, precise identification of the glyco-intermediates will require mass spectrometry or nuclear magnetic resonance (NMR) analysis. When paired with the genetic and biochemical data, structural analyses of the oligosaccharides present on the various forms of immature Rh1 presented here will be highly useful for characterizing N-glycan structure-function relationships.

Studies involving mutations in N-glycan processing enzymes in intact model organisms provide a rich source of information on the physiological functions of these enzymes *in vivo*. Analysis of our collection of *Drosophila* mutants has uncovered unexpected roles for glycosyl hydrolases in Rh1 biosynthesis. Furthermore, given the many severe diseases that result from defects in N-glycosylation, including congenital disorders of glycosylation (CDG), α-mannosidosis, β-mannosidosis, Tay-Sachs disease, and Sandhoff disease, animal models will likely yield important insights into disease mechanisms. In the eye, glycosylation of rhodopsin has long been known to be key for photoreceptor health and function. Mutations that prevent the N-glycosylation of *Drosophila* rhodopsin cause photoreceptor defects and retinal degeneration [38], [85]–[87]. In humans, mutations at the two sites for N-linked glycosylation have been identified in patients with the hereditary retinal degeneration disorder, autosomal dominant retinitis pigmentosa (T4K, N15S and T17M) [88]–[90]. More recently, failures in oligosaccharide trimming during rhodopsin biosynthesis have also been shown to cause photoreceptor defects in *Drosophila* [11]. Consistent with these findings, the original mutants identified in this study originated from a deep pseudopupil (DPP) screen, indicating that they display photoreceptor defects and/or undergo retinal degeneration. Therefore, future studies of these glycosyl hydrolases will likely provide insights into mechanisms of hereditary retinal degeneration disorders, as well as other human diseases. While Rh1 deglycosylation appears to represent a highly unique phenomenon, it remains to be seen whether other glycoproteins are similarly processed and how broadly the ‘catabolic’ glycosyl hydrolases influence glycoprotein processing. Our study represents a critical step towards a genetic dissection of N-glycan processing in *Drosophila*, and yields important insights into the process of N-glycan trimming during Rh1 biosynthesis in the eye.

## Materials and Methods

### *Drosophila* strains

*Drosophila melanogaster* stocks were reared on standard media at 22°C, on a 12∶12 light∶dark cycle. The wild-type stocks used in this study were Canton S. and the parental strain from the EMS-mutagenesis, *brown;scarlet* (*bw;st*). To isolate the *α-man-IIb^G572E^* and *hexo1^Q592X^* mutants, we screened approximately 12,000 EMS (ethyl methyl sulfonate) mutagenized lines from the Zuker collection [41] for the presence or absence of the deep pseudopupil (DPP), as previously described [91]–[93]. Nine hundred DPP-defective stocks were identified and then further screened for defects in Rh1 deglycosylation by Western blot analysis. Deficiency stocks used for mapping the EMS-generated mutations were obtained from the Bloomington Drosophila Stock Center (Indiana University), as were the *P[EP]α-Man-II^G4901^* (Stock #30106), *PBac[RB]Hexo1^e00001^* (Stock #17805), and *Pbac[WH]Hexo2^f04129^* (Stock #18732) P-element alleles. The remaining P-element alleles used in this study were obtained from the Exelixis Collection (Harvard Medical School): *P[XP]α-Man-II^d06422^*, *Pbac[WH]α-Man-IIb^f02524^*, *Pbac[WH]α-Man-IIb^f03535^*, *Pbac[RB]α-Man-IIb^e01163^*, *Pbac[PB]α-Man-IIb^c06077^*, *P[XP]fdl^d03111^*, and *P[XP]Hexo2^d06829^*. The following transgenic *Drosophila* strains, each containing an inducible UAS-RNAi construct directed against either the *α-man-II* or *fused lobes* transcripts, were obtained from the Vienna Drosophila RNAi Center (VDRC): *P[GD2875]^v5838^*, *P[GD1956]^v4637^*, and *P[GD1956]^v4638^*. Eye-specific knockdown of the target mRNA transcripts was achieved by performing standard genetic crosses between the UAS-RNAi strains and a Gal4 driver line provided by Claude Desplan: *P[UAS-Dcr2]*;*P[ey-Gal4]*,*P[IGMR-Gal4]*;+. The Gal4 driver line contained both *eyeless-Gal4* and *lGMR-Gal4* drivers recombined on the second chromosome. Together, these two drivers induced RNAi expression in the whole eye from the time the eye is differentiated through adulthood [61]. The RNAi driver line also harbored a third construct on the X chromosome, *UAS-Dicer2*, to enhance the efficiency of generating small interfering RNA. Finally, mosaic mutants for *α-man-II* were generated with the P-element insertion line *pBac[SAstopDsRed]α-man-II^LL01094^*. Specifically, we used the FLP-FRT, P[GMR-hid] method using an eyeless-FLP driver to induce genetic mosaics and generate mitotic clones of a single genotype in the eye [60]. Both the *α-man-II* allele (DGRC Stock #140255) and the ey-FLP driver line (Bloomington Stock #5253) were generously provided by Junhai Han.

### Deficiency mapping and DNA sequencing of EMS alleles

We performed deficiency mapping to narrow the cytogenetic locations of the two EMS-generated mutations to 89A5 (*α-man-IIb^G572E^*) and 64A12 (*hexo1^Q592X^*) on the third chromosome. To identify the mutant loci, we sequenced prioritized candidate genes within the corresponding deficiency regions. Genomic DNA was isolated from the EMS-mutagenized lines (*α-man-IIb^G572E^* and *hexo1^Q592X^*) and the wild-type parental line (*bw;st*) using the DNeasy Blood and Tissue Kit, according to the manufacturer's instructions (QIAGEN Inc., Valencia, CA). Primer pairs were designed for numerous loci in the deficiency regions, based on their GenBank sequence accession numbers. PCR-amplified DNA sequences were determined by the DNA Sequencing Facility at the University of Wisconsin Biotechnology Center and were aligned using the GAP v4.7 application (The X Window Systems, X11 1.1.3 – XFree86 4.4.0). In the first mutant, we identified a G to A substitution at nucleotide position 1715 in the coding region of CG4606. This mutation corresponds to a substitution at amino acid (aa) 572, from glycine (G) to glutamate (E) (G572E). CG4606 encodes a Class II α-mannosidase enzyme from GH Family 38, termed α-mannosidase-IIb (α-Man-IIb). In the second mutant, we identified a C to T substitution at nucleotide position 1774 in the coding region of CG1318. This mutation generates a premature stop codon (Och) at aa 592, in place of a glutamine (Q) residue (Q592X). CG1318 encodes a β-N-acetylglucosaminidase enzyme from GH Family 20, termed hexosaminidase 1 (Hexo1). Identification of the *α-man-IIb^G572E^* and *hexo1^Q592X^* mutants served as the springboard for a larger analysis of the enzymes involved in N-glycosylation.

### Western blotting

Flies were reared at 22°C and then aged for 1 week at 29°C before head tissue was collected for Western blot analysis. We loaded enough tissue in each case to determine the MW of Rh1. Proteins were separated by electrophoresis in SDS polyacrylamide gels and electroblotted onto nitrocellulose membranes as previously described [39]. Rh1 protein was detected with a monoclonal antibody (4C5) obtained from the Developmental Studies Hybridoma Bank (University of Iowa) [36]. Rh1-bov protein (Figure 3A) was detected with a monoclonal antibody (1D4) directed to the bov epitope, obtained from Phyllis Robinson. The immunoreactive proteins were visualized using horseradish peroxidase (HRP)-conjugated goat anti-mouse (Invitrogen Corporation, Carlsbad, CA) followed by ECL detection (Amersham Pharmecia Biotech, Piscataway, NJ).

### Biochemical procedures

All reagents for the Endo H and PNGase F digestions were obtained from New England BioLabs (Ipswich, MA). Between 20–40 fly heads were homogenized into denaturing buffer, sonicated, and processed according to a modification of the manufacturer's instructions. Specifically, we increased the denaturing buffer to a final concentration of 1% SDS and incubated for 4 hours at 22°C. All samples were mixed with appropriate volumes of sample buffer [94] and assessed via Western blot analysis.

### Heat pulse-chase experiments

For heat pulse-chase experiments, we used transgenic flies expressing wild-type *Drosophila* Rh1 (*ninaE*) tagged with a 12 amino acid epitope from the C-terminus of bovine rhodopsin *(Rh1-bov)* [40]. The epitope tag adds ∼2 kD to Rh1, such that the immature forms are detected at 42 and 40 kD, and the mature form is detected at 36 kD. The tag does not affect Rh1 maturation or function [40]. We used transgenic flies expressing *Rh1-bov* under the control of either the *Drosophila hsp70* heat-shock promoter, *P[hs:Rh1-bov]* [40] or under the control of the endogenous Rh1 promoter, *P[ninaE:Rh1-bov]* [40]. Wild-type flies expressing the *P[hs:Rh1-bov]* construct were exposed to a 1 hour heat shock at 37°C and then shifted back to room temperature and assayed at the indicated times, in hours. Wild-type and *ninaA^269^* mutant flies expressing the *P[ninaE:Rh1-bov]* construct were shown to indicate the mature and immature forms of Rh1 under non-heat shock conditions.

## Supporting Information

Figure S1 Age and temperature data. Western blots of Rh1 protein from flies aged for either 1-day or 10-days at room temperature (22°C) (Lanes 2 and 3) and 1-day or 10-days at 29°C (Lanes 4 and 5). In all cases, lane 1 is a wild-type (WT) control sample aged for 1-day at room temperature (22°C). All Rh1 blots (top) were re-probed with a monoclonal antibody directed to β-tubulin (β-tub) as a loading control (bottom), as indicated in A. (**A**) Wild-type (Canton S.), (**B**) *α-man-II* mutant alleles from top to bottom: *α-man-II^LL01094^*, *α-man-II^v5838^*, *α-man-II^G4901^*, and *α-man-II^d06422^*, (**C**) *α-man-IIb* mutant alleles from top to bottom: *α-man-IIb^G572E^*, *α-man-IIb^f02524^*, *α-man-IIb^f03535^*, *α-man-IIb^e01163^*, and *α-man-IIb^c06077^*, (**D**) *fused lobes* mutant alleles from top to bottom: *fdl^d03111^*, *fdl^v4637^*, *fdl^v4638^*, and (**E**) *hexo1* mutant alleles from top to bottom: *hexo1^Q592X^* and *hexo1^e00001^*.
(PDF)

Figure S2 Glucosidase I and II. Full-length amino acid (aa) alignments between the evolutionarily related human (h) and *Drosophila* (d) glucosidase proteins, generated with the UniProt Align program using the GenBank sequence accession numbers listed in Figure 2. Identical amino acids are marked with asterisks (*), strongly similar amino acids are marked with two dots (:), and weakly similar amino acids are marked with one dot (.). Underlined regions represent predicted transmembrane domains (TMHMM Server v.2.0). The glucosidase I enzyme is encoded by a single locus in humans, GCS1. In contrast, human glucosidase II consists of both an α- and β-subunit, encoded by GANAB and PRKCSH, respectively. In *Drosophila*, we have identified CG1597, CG14476, and CG6453 as the homologs for each of the glucosidase components characterized in humans, respectively (Figure 2). These *Drosophila* genes have not been previously characterized or annotated (FlyBase: [S1]) and here, we show that they display clear homology to the known enzymes in humans. (**A**) Human glucosidase I (GCS1) and the *Drosophila* homolog (CG1597) share 41% overall aa identity and 43% aa identity within the GH Family 63 domain (*Drosophila* aa62–849). (**B**) The α-subunit of human glucosidase II (GANAB) and the *Drosophila* homolog (CG14476) share 47% overall aa identity, 53% identity within the GH Family 31 carbohydrate transport domain (*Drosophila* aa108–891), 58% aa identity within the GH Family 31 general domain (*Drosophila* aa362–699), and 62% identity within the galactose mutarotase-like domain (*Drosophila* aa236–304). Yellow shading indicates the putative catalytic sites and purple shading indicates additional putative active sites [S2–4]. (**C**) The β-subunit of human glucosidase II (PRKCSH) and the *Drosophila* homolog (CG6453) share 39% overall aa identity, 49% aa identity within the PRKCSH-like domain (*Drosophila* aa26–159), and 64% aa identity within the Low Density Lipoprotein Receptor Class A domain (*Drosophila* aa85–120). Blue shading indicates putative calcium-binding sites [S5] and purple shading indicates putative surface binding sites [S6]. Both proteins contain a C-terminal HDEL endoplasmic reticulum retention signal (bold) [S7].
(PDF)

Figure S3 Class I α1,2-mannosidases (GH Family 47). There are seven members of GH Family 47 in humans, divided into three major subcategories based on their cellular localization and biochemical properties, including the ER subfamily (Subgroup A, orange), the Golgi subfamily (Subgroup B, yellow), and the Edem subfamily (Subgroup C, green). We have identified five GH Family 47 mannosidases in *Drosophila*. Amino acid sequence analysis revealed significant homology between specific human and *Drosophila* enzymes in GH Family 47, allowing us to assign the *Drosophila* proteins to specific subfamilies, as outlined below and in Figure 2. (**A**) Phylogenetic tree depicting the predicted evolutionary relationships between the Class I α-mannosidases from GH Family 47 in humans (h) and *Drosophila* (d) generated with the UniProt Align program using the GenBank sequence accession numbers listed in Figure 2. Black arrows designate speciation of the last common ancestor between humans and flies, leading to the production of orthologs. White arrows denote presumed gene duplication events, leading to the production of paralogs. According to these data, multiple gene duplication events within the Golgi subfamily (Subgroup B, yellow) likely occurred late in evolution, independently giving rise to both the mammalian gene family [S8] and the *Drosophila* gene pair [S9]. Therefore, in the last common ancestor between *Drosophila* and humans (see dotted line), there was likely only a single Golgi α1,2-mannosidase gene (yellow). This hypothesis is further supported for the human loci by the similar positioning of intron/exon boundaries [S10]. The particularly high overall aa identity (60%) and overlap in enzymatic specificity between human Golgi MAN IA and Golgi MAN IB suggests that they represent one of the most recent gene duplications in the α-mannosidase family [S8]. As with the human loci, it has been proposed that a duplication event, occurring at some point during evolution of the *Drosophila* lineage, gave rise to CG31202 from its parental locus, α-Man-I (CG42275) [S9]. Therefore, although the human and *Drosophila* Golgi loci evolved from a common ancestral locus, unique duplication events in each lineage gave rise to the multiple forms that exist today and detailed biochemical confirmation of the precise functions of the *Drosophila* enzymes is lacking. In contrast to Subgroup B, the phylogeny suggests that three EDEM (ER degradation enhancing α-mannosidase-like) proteins were present in the last common ancestor between *Drosophila* and humans (Subgroup C, green). However, the most extensively studied EDEM in humans, EDEM1, does not appear to have a direct counterpart in *Drosophila*. This would suggest that the ortholog of mammalian EDEM1 was lost throughout the course of evolution in the *Drosophila* lineage. (**B**) Partial amino acid (aa) alignment between the human (h) and *Drosophila* (d) Class I α-mannosidases, generated with the UniProt Align program using the GenBank sequence accession numbers listed in Figure 2. Numbers refer to aa residues. Highlighted in red is a critical arginine (R) residue, conserved only among members of Subgroup A (orange). This R residue is a hallmark of ERManI enzymes and is responsible for the high level of specificity in cleaving M10 on branch B (Figure 1). Among the five GH Family 47 members identified in *Drosophila*, α-Man-Ib is the only mannosidase that harbors this R residue. All other GH Family 47 members contain either a leucine (Subgroup B, yellow) or a glycine (Subgroup C, green) at this position. (**C**) Full-length aa alignment between human (h), *Drosophila* (d), and yeast (y) GH Family 47 members from Subgroup A, generated with the UniProt Align program using the GenBank sequence accession numbers listed in Figure 2. Identical amino acids are marked with asterisks (*), strongly similar amino acids are marked with two dots (:), and weakly similar amino acids are marked with one dot (.). Underlined regions represent predicted transmembrane domains (TMHMM Server v.2.0).The first trimming event mediated by GH Family 47 involves the removal of a terminal mannose residue (M10) on branch B (Figure 1), to generate Man_8_GlcNAc_2_ [49], [50]. The enzyme responsible for this step is an ER resident α1,2-mannosidase, also known as ER α-mannosidase I (ERManI). ERManI is the sole member of Subgroup A within GH Family 47 and has been well characterized in *S. cerevisiae* (MNS1, NP_012665.1) [S11] and in humans (MAN1B1) [S12,S13]. *Drosophila* α-Man-Ib displays 41% overall aa identity with human ERManI and 54% aa identity within the GH Family 47 domain (*Drosophila* aa242–680). For a description of the aa shading, see below. (**D**) Full-length aa alignment between the human (h) and *Drosophila* (d) GH Family 47 members from Subgroup B, generated with the UniProt Align program using the GenBank sequence accession numbers listed in Figure 2. Identical amino acids are marked with asterisks (*), strongly similar amino acids are marked with two dots (:), and weakly similar amino acids are marked with one dot (.). Underlined regions represent predicted transmembrane domains (TMHMM Server v.2.0). These Golgi α1,2-mannosidases have the capacity to cleave all four α1,2-linked mannose residues (M6, M9, M10, and M11) on the N-glycan structure (Figure 1) [49]. The mammalian enzymes, referred to as Golgi Man IA, IB, and IC, are encoded by MAN1A1 [S14,S15], MAN1A2 [S16,S17], and MAN1C1 [S10] in humans, respectively (Figure 2). Although these enzymes preferentially cleave specific α1,2-linked mannose residues from the N-glycan, their functions are largely overlapping [49]. In general, the Golgi α1,2-mannosidases have high specificity for M6, M9, and M11, located on branches A and C of the N-glycan, with significantly lower specificity for M10, located on branch B (Figure 1). In this way, their function is complementary to the role of ERManI, which primarily cleaves the central mannose residue (M10) and has very low specificity for α1,2-linked residues on branches A and C [49,S18]. We have identified two GH Family 47 mannosidases from Subgroup B in *Drosophila*, namely α-mannosidase-I (α-Man-I, CG42275) and an unannotated locus, CG31202 (Figure 2). The α-Man-I enzyme displays between 44–48% overall aa identity with human Golgi Man IA, IB, and IC and between 56–60% identity with these enzymes in the GH Family 47 domain (*Drosophila* aa198–647). In contrast, CG31202 display only 37–39% overall aa identity with the human enzymes and only 41–43% identity within the GH Family 47 domain (*Drosophila* aa78–512). For a description of the aa shading, see below. (**E/F**) Full-length aa alignments between GH Family 47 members from Subgroup C, including (E) human EDEM2 aligned with *Drosophila* Edem1 (CG3810) and (F) human EDEM3 aligned with *Drosophila* Edem2 (CG5682), generated with the UniProt Align program using the GenBank sequence accession numbers listed in Figure 2. Identical amino acids are marked with asterisks (*), strongly similar amino acids are marked with two dots (:), and weakly similar amino acids are marked with one dot (.). Underlined regions represent predicted transmembrane domains (TMHMM Server v.2.0). Class I α-mannosidases from Subgroup C represent the most recent additions to GH Family 47, and comprise a group of ER-resident proteins [S19]. In contrast to the other Class I α-mannosidase enzymes,that function in the normal biosynthetic trimming of newly synthesized glycoproteins, members of Subgroup C function in quality control by targeting misfolded glycoproteins for ER-associated degradation (ERAD) [S20]. Three members of this group, EDEM1 [S21], EDEM2 [S22], and EDEM3 [S23] have been identified in mammals (Figure 2). In contrast, there are only two members of Subgroup C in *Drosophila*, Edem1 (CG3810) and Edem2 (CG5682) (Figure 2). *Drosophila* Edem1 displays 54% overall aa identity with human EDEM2 and 64% aa identity within the GH Family 47 domain (*Drosophila* aa48–488). *Drosophila* Edem2 displays 48% overall aa identity with human EDEM3 and 62% aa identity within the GH Family 47 domain (*Drosophila* aa57–494). Human EDEM1 is considerably divergent from the other members of this family and thus has been omitted from either alignment. For a description of the aa shading, see below. **Description of amino acid shading in C–F:** Class I α1,2-mannosidases have been extensively studied with regard to enzyme kinetics, substrate specificity, structure, and catalytic mechanism [50]. Crystal structures for several members of GH Family 47 have been solved, including yeast MNS1 [S24], human ERManI [S25], murine MAN1A1 [S26], an α1,2-mannosidase from *P. citrinum* [S27], and an α1,2-mannosidase from *T. reesei* [S28]. These structures, in combination with extensive site-directed mutagenesis [S29–33] and computational modeling [S34], reveal critical insights into the aa residues that are essential for the enzymatic activity of GH Family 47 enzymes. Highly conserved amino acids that define this evolutionarily related group of enzymes are highlighted, as described below. **Green shading** indicates two cysteine residues (lacking in Edems) that form a disulfide bond that is essential for stabilizing the tertiary structure of the molecule. **Blue shading** denotes residues that are either directly or indirectly required for Ca^2+^ binding. **Yellow shading** highlights the catalytic acid (proton donor) and catalytic base that are directly involved in hydrolysis of the glycoside bond. **Purple shading** denotes a host of additional amino acids that play critical roles in the energetics of substrate binding. Finally, as explained in Figure S3B above, **red shading** highlights a critical amino acid position that houses either an arginine (R) residue in members from Subgroup A, a leucine (L) residue in members from Subgroup B, or a glycine (G) residue in members from Subgroup C. This distinction significantly alters the specificity of N-glycan processing. Mutating this R to L, in both yeast MNS1 [S33] and human ERManI [S29], dramatically broadens substrate specificity, allowing the enzymes to cleave additional α1,2-linkages and leading to the production of atypical Man_8-6_GlcNAc_2_ structures. Furthermore, crystallization of yeast MNS1 has confirmed the importance of this residue by revealing that it makes direct contact with four unique residues on the N-glycan and serves to stabilize the central arm of the N-glycan during cleavage of M10 [S24]. In contrast, crystallization of a broad specificity α1,2-mannosidase from *P. citrinum* has indicated that when a smaller residue (G in this case) is present at this position, the active site is enlarged, allowing for a greater degree of flexibility in the binding and trimming of additional mannose residues on the A and C branches of the N-glycan (Figure 1) [S27].
(PDF)

Figure S4 GlcNAc-transferase I. Full-length amino acid (aa) alignment between human (h) and *Drosophila* (d) GlcNAc-transferase I, generated with the UniProt Align program using the GenBank sequence accession numbers listed in Figure 2. Identical amino acids are marked with asterisks (*), strongly similar amino acids are marked with two dots (:), and weakly similar amino acids are marked with one dot (.). Underlined regions represent predicted transmembrane domains (TMHMM Server v.2.0). Following trimming to Man_5_GlcNAc_2_, the classical pathway for oligosaccharide maturation involves the action of GlcNAc-transferase I, which functions in the addition of a single β1,2-linked N-acetylglucosamine (GlcNAc) to a terminal mannose (M4) on branch A (Figure 4) [51], [52]. This modification occurs in the Golgi and is a prerequisite for the formation of complex N-glycans. The enzyme responsible for this function (also called Mgat1) is highly conserved between *Drosophila* (CG13431) and humans (MGAT1). *Drosophila* Mgat1 displays 51% overall aa identity with human MGAT1 and 62% aa identity within the GT13 GlcNAc-TI domain (*Drosophila* aa119–449). Purple shading indicates putative substrate binding sites [S35].
(PDF)

Figure S5 Class II α1,2-mannosidases (GH Family 38). There are five GH Family 38 members in humans, divided into three major subcategories based on their cellular localization and biochemical properties, including the Golgi subfamily (Subgroup A, blue), the lysosomal subfamily (Subgroup B, purple) and the ERManII subfamily (Subgroup C, grey). We have identified eight GH Family 38 mannosidases in *Drosophila*. Amino acid sequence analysis reveals significant homology between specific human and *Drosophila* enzymes in GH Family 38, allowing us to assign the *Drosophila* proteins to specific subfamilies, as outlined below and in Figure 2. (**A**) Phylogenetic tree depicting the predicted evolutionary relationships between the Class II α-mannosidases from GH Family 38 in humans (h) and *Drosophila* (d), generated with the UniProt Align program using the GenBank sequence accession numbers listed in Figure 2. Black arrows designate speciation of the last common ancestor between humans and flies, leading to the production of orthologs. White arrows denote presumed gene duplication events, leading to the production of paralogs. The *Drosophila* lysosomal α-mannosidase loci (purple) have undoubtedly resulted from more recent duplication events and, accordingly, display between 65–77% overall aa identity with one another. In a recent study, these lysosomal α-mannosidases were designated LManI (CG5322), LManII (CG6206), LManIII (CG9463), LManIV (CG9465), LManV (CG9466), and LManVI (CG9468) [46]. Further evidence for their recent duplication is the positioning of the corresponding loci within the genome. The loci encoding LManI and II are organized back-to-back at 31E5, whereas the loci encoding LManIII-VI are organized in tandem at 29F1. (**B**) Full-length amino acid (aa) alignment between GH Family 38 members from Subgroup A, including human (h) α-Man II (MAN2A1), human α-Man IIx (MAN2A2), *Drosophila* (d) α-Man-II (CG18802), and *Drosophila* α-Man-IIb (CG4606), generated with the UniProt Align program using the GenBank sequence accession numbers listed in Figure 2. Identical amino acids are marked with asterisks (*), strongly similar amino acids are marked with two dots (:), and weakly similar amino acids are marked with one dot (.). Underlined regions represent predicted transmembrane domains (TMHMM Server v.2.0). *Drosophila* α-Man-II displays 40% overall aa identity with both human α-Man II and α-Man IIx, 56–57% aa identity within the GH Family 38 N-terminal catalytic domain (*Drosophila* aa143–482), and 55–56% aa identity within the GH Family 38 Middle domain (*Drosophila* aa477–553). By comparison, *Drosophila* α-Man-IIb is significantly more divergent, displaying only 33–34% overall aa identity with the human enzymes, 32–46% aa identity within the GH Family 38 N-terminal catalytic domain (*Drosophila* aa167–514), and 51–52% aa identity within the GH Family 38 Middle domain (*Drosophila* aa508–596). For a description of the aa shading, see below. (**C**) Full-length amino acid (aa) alignment between human (h) and *Drosophila* (d) GH Family 38 members from Subgroup B, generated with the UniProt Align program using the GenBank sequence accession numbers listed in Figure 2. Identical amino acids are marked with asterisks (*), strongly similar amino acids are marked with two dots (:), and weakly similar amino acids are marked with one dot (.). In humans, there are two lysosomal α-mannosidases from GH Family 38 Subgroup B, namely the major lysosomal α-mannosidases (Major Lys, MAN2B1) and the minor lysosomal α-mannosidases (MAN2B2) (Figure 2). These enzymes are required for removal of the M4 and M5 mannose residues from the N-glycan (Figure 1), and have been shown to play a vital role in maintaining cellular homeostasis [73]. The major lysosomal α-mannosidase has high specificity for cleavage of M4, whereas the minor lysosomal α-mannosidase preferentially cleaves M5 (Figure 1). The complementary actions of these two enzymes, along with their ubiquitous expression, suggest that they function in tandem for N-glycan degradation in mammals. Consistent with their lysosomal localization, optimum activity for these enzymes occurs between pH 4.0–4.2 [66], [71,S36]. There are six homologous loci in *Drosophila*, CG5322 (LManI), CG6206 (LManII), CG9463 (LManIII), CG9465 (LManIV), CG9466 (LManV), and CG9468 (LManVI) [46]. All six *Drosophila* lysosomal mannosidases display 39–44% overall aa identity with the major lysosomal α-mannosidase in humans (MAN2B1), 45–50% aa identity within the GH Family 38 Middle domain (*Drosophila* CG5322 aa323–398), and 55–60% aa identity within the GH Family 38 N-terminal catalytic domain (*Drosophila* CG5322 aa2–276). In stark contrast, the *Drosophila* lysosomal α-mannosidases have little to no aa identity with the minor lysosomal α-mannosidase in humans. In fact, the minor lysosomal α-mannosidase in humans is so divergent from the other members of this family, that it has been omitted from the alignment. For a description of the aa shading, see below. **Description of amino acid shading in B and C:** Class II α-mannosidases have been extensively studied with regard to enzyme kinetics, substrate specificity, structure, and catalytic mechanism. X-ray crystallography has been used to determine high-resolution structures of both wild-type and mutant *Drosophila* α-Man-II enzymes, as well as numerous covalent reaction intermediates, bound to various inhibitors, synthetic substrates, and/or natural oligosaccharide substrates [12]–[22]. Structural analysis of covalent reaction intermediates has also been achieved for other members of GH Family 38 by liquid chromatography-mass spectrometry (LC-MS), including Jack Bean α-mannosidase [S37] and a lysosomal α-mannosidase from bovine kidney [S38]. These structures, in combination with site-directed mutagenesis, provide critical insight into the aa residues that are essential for the enzymatic activity of GH Family 38 enzymes. Highly conserved amino acids that define this evolutionarily related group of enzymes are highlighted, as described below. **Yellow shading** highlights the nucleophile and acid/base catalyst that are directly involved in hydrolysis of the glycoside bond. **Dark blue shading** indicates residues that are required for Zn^2+^ binding. **Purple shading** denotes a host of additional amino acids that play critical roles in the energetics of substrate binding. Some highlighted residues only pertain to subgroup A: **Pink shading** indicates residues involved in the holding site, which is only found in enzymes that cleave the M7 and M8 mannose residues (Figure 1). **Red shading** denotes residues involved in the anchor site, which is only found in enzymes that require the presence of a terminal GlcNAc on branch A in order to cleave M7 and M8. **Green shading** indicates four cysteine residues that form two different disulfide bonds, one of which is lacking in *Drosophila* α-Man-IIb. In Figure S5B, **light blue shading** shows the single residue mutated in our *Drosophila* α-Man-IIb EMS allele (G572E). In Figure S5C, **light blue shading** denotes missense mutations that cause α-mannosidosis in humans [S39].
(PDF)

Figure S6 Hexosaminidases (GH Family 20). (**A**) Phylogenetic tree depicting the predicted evolutionary relationships between the hexosaminidases from GH Family 20 in humans (h) and *Drosophila* (d), generated with the UniProt Align program using the GenBank sequence accession numbers listed in Figure 2. This list includes the human β-hexosaminidase, α-chain (HEXA), the human β-hexosaminidase, β-chain (HEXB), *Drosophila* hexosaminidase 1 (Hexo1, CG1318), *Drosophila* hexosamindase 2 (Hexo2, CG1787), and *Drosophila* fused lobes (Fdl, CG8824). Black arrows designate speciation of the last common ancestor between humans and flies, leading to the production of orthologs. White arrows denote presumed gene duplication events, leading to the production of paralogs. Based on this analysis, there was likely a single hexosaminidase in the last common ancestor between humans and *Drosophila*, thus the human gene pair and the *Drosophila* gene triplet have evolved separately through independent duplication events. (**B**) Full-length amino acid (aa) alignments between evolutionarily related human (h) and *Drosophila* (d) hexosaminidases from GH Family 20, generated with the UniProt Align program using the GenBank sequence accession numbers listed in Figure 2. This list includes the human β-hexosaminidase, α-chain (HEXA), the human β-hexosaminidase, β-chain (HEXB), *Drosophila* Hexo1 (CG1318), *Drosophila* Hexo2 (CG1787), and *Drosophila* fused lobes (CG8824). Identical amino acids are marked with asterisks (*), strongly similar amino acids are marked with two dots (:), and weakly similar amino acids are marked with one dot (.). Underlined regions represent predicted transmembrane domains (TMHMM Server v.2.0). All pair-wise comparisons between the human and *Drosophila* proteins yield 30–32% overall aa identity and 35–38% aa identity within the GH Family 20 catalytic domain (d Hexo1 aa214–557, d Hexo2 aa235–581, and d fused lobes aa276–613). Crystallization, photoaffinity labeling, expression of chimeric subunits, and site-directed mutagenesis of the two human lysosomal β-hexosaminidases reveal a number of aa residues that are critical for the enzymatic activity of GH Family 20 [S40–47]. Highly conserved amino acids that define this evolutionarily related group of enzymes are highlighted, as described below. **Yellow shading** highlights the general acid/base catalyst. Green shading indicates cysteine residues required for disulfide bond formation. **Purple shading** denotes a host of additional residues that contribute to the enzymatic activity of the molecule by hydrogen bonding, substrate binding or stabilization of reaction intermediates. **Red shading** indicates three regions found only in the α-chain (HEXA), which are required for its specific interaction with and hydrolysis of GM2 (ganglioside). Finally, **light blue shading** highlights a large number of missense mutations identified in either human HEXA or human HEXB, that are associated with Tay-Sachs disease or Sandhoff disease, respectively [32].
(PDF)

Figure S7 β-Mannosidases (GH Family 2). Full-length amino acid (aa) alignment between human (h) β-mannosidase (MANBA) and the *Drosophila* (d) homolog, CG12582, from GH Family 2, generated with the UniProt Align program using the GenBank sequence accession numbers listed in Figure 2. Identical amino acids are marked with asterisks (*), strongly similar amino acids are marked with two dots (:), and weakly similar amino acids are marked with one dot (.). *Drosophila* CG12582 displays 34% overall aa identity with human β-Man and 51% aa identity within the GH Family 2 TIM barrel domain (*Drosophila* aa343–457). Crystallization and site-directed mutagenesis of a β-mannosidase from *Bacteroides thetaiotaomicron* have revealed a number of critical residues that are highly conserved among all members of GH Family 2 [S48]. **Yellow shading** indicates the two catalytic nucleophiles. **Purple shading** denotes other critical residues that either contribute to the enzyme's catalytic activity or are important for substrate recognition and binding. Finally, **light blue shading** highlights four missense mutations that cause β-mannosidosis in humans [30]. Importantly, all of these key residues are conserved in the putative *Drosophila* β-mannosidase (CG12582).
(PDF)

## References

[1] Roth J, Zuber C, Park S, Jang I, Lee Y, et al (2010) Protein N-glycosylation, protein folding, and protein quality control. Mol Cells 30: 497–506. 21340671 10.1007/s10059-010-0159-z

[2] Katoh T, Tiemeyer M (2013) The N's and O's of *Drosophila* glycoprotein glycobiology. Glycoconj J 30: 57–66. 22936173 10.1007/s10719-012-9442-x PMC3548036

[3] Kornfeld R, Kornfeld S (1985) Assembly of asparagine linked oligosaccharides. Annu Rev Biochem 54: 631–664. 3896128 10.1146/annurev.bi.54.070185.003215

[4] Silberstein S, Gilmore R (1996) Biochemistry, molecular biology, and genetics of the oligosaccharyltransferase. FASEB J 10: 849–858. 8666161

[5] Stanley P (2011) Golgi glycosylation. Cold Spring Harb Perspect Biol 3: : pii: a005199. doi: 10.1101/cshperspect.a005199. 10.1101/cshperspect.a005199 PMC3062213 21441588

[6] Caramelo JJ, Parodi AJ (2007) How sugars convey information on protein conformation in the endoplasmic reticulum. Semin Cell Dev Biol 18: 732–742. 17997334 10.1016/j.semcdb.2007.09.006 PMC2196135

[7] Kerscher S, Albert S, Wucherpfennig D, Heisenberg M, Schneuwly S (1995) Molecular and genetic analysis of the *Drosophila* mas-1 (mannosidase-1) gene which encodes a glycoprotein processing alpha 1,2-mannosidase. Dev Biol 168: 613–626. 7729592 10.1006/dbio.1995.1106

[8] Parker GF, Williams PJ, Butters TD, Roberts DB (1991) Detection of the lipid-linked precursor oligosaccharide of N-linked protein glycosylation in *Drosophila melanogaster* . FEBS Lett 290: 58–60. 1915893 10.1016/0014-5793(91)81225-w

[9] Williams PJ, Wormald MR, Dwek RA, Rademacher TW, Parker GF, et al (1991) Characterisation of oligosaccharides from *Drosophila melanogaster* glycoproteins. Biochim Biophys Acta 1075: 146–153. 1932070 10.1016/0304-4165(91)90245-c

[10] Leonard R, Rendic D, Rabouille C, Wilson IB, Preat T, et al (2006) The *Drosophila fused lobes* gene encodes an N-acetylglucosaminidase involved in N-glycan processing. J Biol Chem 281: 4867–4875. 16339150 10.1074/jbc.M511023200

[11] Cao J, Li Y, Xia W, Reddig K, Hu W, et al (2011) A *Drosophila* metallophosphoesterase mediates deglycosylation of rhodopsin. EMBO J 30: 3701–3713. 21804530 10.1038/emboj.2011.254 PMC3173788

[12] Englebienne P, Fiaux H, Kuntz DA, Corbeil CR, Gerber-Lemaire S, et al (2007) Evaluation of docking programs for predicting binding of Golgi alpha-mannosidase II inhibitors: a comparison with crystallography. Proteins 69: 160–176. 17557336 10.1002/prot.21479

[13] Fiaux H, Kuntz DA, Hoffman D, Janzer RC, Gerber-Lemaire S, et al (2008) Functionalized pyrrolidine inhibitors of human type II alpha-mannosidases as anti-cancer agents: optimizing the fit to the active site. Bioorg Med Chem 16: 7337–7346. 18599296 10.1016/j.bmc.2008.06.021

[14] Kawatkar SP, Kuntz DA, Woods RJ, Rose DR, Boons GJ (2006) Structural basis of the inhibition of Golgi alpha-mannosidase II by mannostatin A and the role of the thiomethyl moiety in ligand-protein interactions. J Am Chem Soc 128: 8310–8319. 16787095 10.1021/ja061216p PMC2553320

[15] Kuntz DA, Zhong W, Guo J, Rose DR, Boons GJ (2009) The molecular basis of inhibition of Golgi alpha-mannosidase II by mannostatin A. Chembiochem 10: 268–277. 19101978 10.1002/cbic.200800538 PMC3956299

[16] Kuntz DA, Nakayama S, Shea K, Hori H, Uto Y, et al (2010) Structural investigation of the binding of 5-substituted swainsonine analogues to Golgi alpha-mannosidase II. Chembiochem 11: 673–680. 20209559 10.1002/cbic.200900750

[17] Numao S, Kuntz DA, Withers SG, Rose DR (2003) Insights into the mechanism of *Drosophila melanogaster* Golgi alpha-mannosidase II through the structural analysis of covalent reaction intermediates. J Biol Chem 278: 48074–48083. 12960159 10.1074/jbc.M309249200

[18] Polakova M, Sestak S, Lattova E, Petrus L, Mucha J, et al (2011) alpha-D-mannose derivatives as models designed for selective inhibition of Golgi alpha-mannosidase II. Eur J Med Chem 46: 944–952. 21295890 10.1016/j.ejmech.2011.01.012

[19] Shah N, Kuntz DA, Rose DR (2003) Comparison of kifunensine and 1-deoxymannojirimycin binding to class I and II alpha-mannosidases demonstrates different saccharide distortions in inverting and retaining catalytic mechanisms. Biochemistry 42: 13812–13816. 14636047 10.1021/bi034742r

[20] Shah N, Kuntz DA, Rose DR (2008) Golgi alpha-mannosidase II cleaves two sugars sequentially in the same catalytic site. Proc Natl Acad Sci U S A 105: 9570–9575. 18599462 10.1073/pnas.0802206105 PMC2474516

[21] van den Elsen JM, Kuntz DA, Rose DR (2001) Structure of Golgi alpha-mannosidase II: a target for inhibition of growth and metastasis of cancer cells. EMBO J 20: 3008–3017. 11406577 10.1093/emboj/20.12.3008 PMC150216

[22] Zhong W, Kuntz DA, Ember B, Singh H, Moremen KW, et al (2008) Probing the substrate specificity of Golgi alpha-mannosidase II by use of synthetic oligosaccharides and a catalytic nucleophile mutant. J Am Chem Soc 130: 8975–8983. 18558690 10.1021/ja711248y PMC3982601

[23] Jaeken J, Matthijs G (2007) Congenital disorders of glycosylation: a rapidly expanding disease family. Annu Rev Genomics Hum Genet 8: 261–278. 17506657 10.1146/annurev.genom.8.080706.092327

[24] Grunewald S, Matthijs G (2000) Congenital disorders of glycosylation (CDG): a rapidly expanding group of neurometabolic disorders. Neuropediatrics 31: 57–59. 10832577 10.1055/s-2000-7487

[25] Kahook MY, Mandava N, Bateman JB, Thomas JA (2006) Glycosylation type Ic disorder: idiopathic intracranial hypertension and retinal degeneration. Br J Ophthalmol 90: 115–116. 16361681 10.1136/bjo.2005.080648 PMC1478164

[26] Morava E, Wosik HN, Sykut-Cegielska J, Adamowicz M, Guillard M, et al (2009) Ophthalmological abnormalities in children with congenital disorders of glycosylation type I. Br J Ophthalmol 93: 350–354. 19019927 10.1136/bjo.2008.145359

[27] Bedilu R, Nummy KA, Cooper A, Wevers R, Smeitink J, et al (2002) Variable clinical presentation of lysosomal beta-mannosidosis in patients with null mutations. Mol Genet Metab 77: 282–290. 12468273 10.1016/s1096-7192(02)00172-5

[28] Malm D, Nilssen O (2008) Alpha-mannosidosis. Orphanet J Rare Dis 3: 21. 18651971 10.1186/1750-1172-3-21 PMC2515294

[29] Kleijer WJ, Hu P, Thoomes R, Boer M, Huijmans JG, et al (1990) Beta-mannosidase deficiency: heterogeneous manifestation in the first female patient and her brother. J Inherit Metab Dis 13: 867–872. 2079835 10.1007/BF01800211

[30] Huynh T, Khan JM, Ranganathan S (2011) A comparative structural bioinformatics analysis of inherited mutations in beta-D-Mannosidase across multiple species reveals a genotype-phenotype correlation. BMC Genomics 12 Suppl 3: S22. 22369051 10.1186/1471-2164-12-S3-S22 PMC3333182

[31] Samra ZQ, Athar MA (2008) Cloning, sequence, expression and characterization of human beta-mannosidase. Acta Biochim Pol 55: 479–490. 18800177

[32] Mahuran DJ (1999) Biochemical consequences of mutations causing the GM2 gangliosidoses. Biochim Biophys Acta 1455: 105–138. 10571007 10.1016/s0925-4439(99)00074-5

[33] Patnaik SK, Stanley P (2006) Lectin-resistant CHO glycosylation mutants. Methods Enzymol 416: 159–182. 17113866 10.1016/S0076-6879(06)16011-5

[34] Elbein AD (1991) Glycosidase inhibitors as antiviral and/or antitumor agents. Semin Cell Biol 2: 309–317. 1813022

[35] Moremen KW (2002) Golgi alpha-mannosidase II deficiency in vertebrate systems: implications for asparagine-linked oligosaccharide processing in mammals. Biochim Biophys Acta 1573: 225–235. 12417404 10.1016/s0304-4165(02)00388-4

[36] de Couet HG, Tanimura T (1987) Monoclonal antibodies provide evidence that rhodopsin in the outer rhabdomeres of *Drosophila melanogaster* is not glycosylated. Eur J Cell Biol 44: 50–56.

[37] Huber A, Smith DP, Zuker CS, Paulsen R (1990) Opsin of *Calliphora* peripheral photoreceptors R1-6. Homology with *Drosophila* Rh1 and posttranslational processing. J Biol Chem 265: 17906–17910. 1698782

[38] Webel R, Menon I, O'Tousa J, Colley NJ (2000) Role of asparagine-linked glycosylation sites in Rhodopsin maturation and association with its molecular chaperone, NinaA. J Biol Chem 275: 24752–24759. 10811808 10.1074/jbc.M002668200

[39] Colley NJ, Baker EK, Stamnes MA, Zuker CS (1991) The cyclophilin homolog *ninaA* is required in the secretory pathway. Cell 67: 255–263. 1913822 10.1016/0092-8674(91)90177-z

[40] Colley NJ, Cassill JA, Baker EK, Zuker CS (1995) Defective intracellular transport is the molecular basis of rhodopsin-dependent dominant retinal degeneration. Proc Natl Acad Sci U S A 92: 3070–3074. 7708777 10.1073/pnas.92.7.3070 PMC42361

[41] Koundakjian EJ, Cowan DM, Hardy RW, Becker AH (2004) The Zuker collection: a resource for the analysis of autosomal gene function in *Drosophila melanogaster* . Genetics 167: 203–206. 15166147 10.1534/genetics.167.1.203 PMC1470872

[42] Schneuwly S, Shortridge RD, Larrivee DC, Ono T, Ozaki M, et al (1989) *Drosophila ninaA* gene encodes an eye-specific cyclophilin (cyclosporin A binding protein). Proc Natl Acad Sci U S A 86: 5390–5394. 2664782 10.1073/pnas.86.14.5390 PMC297628

[43] Shieh BH, Stamnes MA, Seavello S, Harris GL, Zuker CS (1989) The *ninaA* gene required for visual transduction in *Drosophila* encodes a homologue of cyclosporin A-binding protein. Nature 338: 67–70. 2493138 10.1038/338067a0

[44] Stamnes MA, Shieh BH, Chuman L, Harris GL, Zuker CS (1991) The cyclophilin homolog NinaA is a tissue-specific integral membrane protein required for the proper synthesis of a subset of *Drosophila* rhodopsins. Cell 65: 219–227. 1707759 10.1016/0092-8674(91)90156-s

[45] Baker EK, Colley NJ, Zuker CS (1994) The cyclophilin homolog NinaA functions as a chaperone, forming a stable complex in vivo with its protein target rhodopsin. EMBO J 13: 4886–4895. 7957056 10.1002/j.1460-2075.1994.tb06816.x PMC395429

[46] Nemcovicova I, Sestak S, Rendic D, Plskova M, Mucha J, et al (2013) Characterisation of class I and II alpha-mannosidases from *Drosophila melanogaster* . Glycoconj J 30: 899–909. 23979800 10.1007/s10719-013-9495-5

[47] Cattaneo F, Pasini ME, Intra J, Matsumoto M, Briani F, et al (2006) Identification and expression analysis of *Drosophila melanogaster* genes encoding beta-hexosaminidases of the sperm plasma membrane. Glycobiology 16: 786–800. 16733265 10.1093/glycob/cwl007

[48] Herscovics A (1999) Importance of glycosidases in mammalian glycoprotein biosynthesis. Biochim Biophys Acta 1473: 96–107. 10580131 10.1016/s0304-4165(99)00171-3

[49] Herscovics A (2001) Structure and function of Class I alpha 1,2-mannosidases involved in glycoprotein synthesis and endoplasmic reticulum quality control. Biochimie 83: 757–762. 11530208 10.1016/s0300-9084(01)01319-0

[50] Mast SW, Moremen KW (2006) Family 47 alpha-mannosidases in N-glycan processing. Methods Enzymol 415: 31–46. 17116466 10.1016/S0076-6879(06)15003-X PMC3964790

[51] Harpaz N, Schachter H (1980) Control of glycoprotein synthesis. Processing of asparagine-linked oligosaccharides by one or more rat liver Golgi alpha-D-mannosidases dependent on the prior action of UDP-N-acetylglucosamine: alpha-D-mannoside beta 2-N-acetylglucosaminyltransferase I. J Biol Chem 255: 4894–4902. 6445359

[52] Schachter H, Chen SH, Zhou S, Tan J, Yip B, et al (1997) Structure and function of the genes encoding N-acetylglucosaminyltransferases which initiate N-glycan antennae. Biochem Soc Trans 25: 875–880. 9388565 10.1042/bst0250875

[53] Schachter H (1991) The ‘yellow brick road’ to branched complex N-glycans. Glycobiology 1: 453–461. 1840403 10.1093/glycob/1.5.453

[54] Howard S, Braun C, McCarter J, Moremen KW, Liao YF, et al (1997) Human lysosomal and jack bean alpha-mannosidases are retaining glycosidases. Biochem Biophys Res Commun 238: 896–898. 9325188 10.1006/bbrc.1997.7148

[55] Maley F, Trimble RB, Tarentino AL, Plummer TH Jr (1989) Characterization of glycoproteins and their associated oligosaccharides through the use of endoglycosidases. Anal Biochem 180: 195–204. 2510544 10.1016/0003-2697(89)90115-2

[56] Akama TO, Nakagawa H, Wong NK, Sutton-Smith M, Dell A, et al (2006) Essential and mutually compensatory roles of alpha-mannosidase II and alpha-mannosidase IIx in N-glycan processing *in vivo* in mice. Proc Natl Acad Sci U S A 103: 8983–8988. 16754854 10.1073/pnas.0603248103 PMC1474017

[57] Moremen KW, Robbins PW (1991) Isolation, characterization, and expression of cDNAs encoding murine alpha-mannosidase II, a Golgi enzyme that controls conversion of high mannose to complex N-glycans. J Cell Biol 115: 1521–1534. 1757461 10.1083/jcb.115.6.1521 PMC2289207

[58] Misago M, Liao YF, Kudo S, Eto S, Mattei MG, et al (1995) Molecular cloning and expression of cDNAs encoding human alpha-mannosidase II and a previously unrecognized alpha-mannosidase IIx isozyme. Proc Natl Acad Sci U S A 92: 11766–11770. 8524845 10.1073/pnas.92.25.11766 PMC40483

[59] Fukuda MN, Masri KA, Dell A, Luzzatto L, Moremen KW (1990) Incomplete synthesis of N-glycans in congenital dyserythropoietic anemia type II caused by a defect in the gene encoding alpha-mannosidase II. Proc Natl Acad Sci U S A 87: 7443–7447. 2217175 10.1073/pnas.87.19.7443 PMC54763

[60] Stowers RS, Schwarz TL (1999) A genetic method for generating *Drosophila* eyes composed exclusively of mitotic clones of a single genotype. Genetics 152: 1631–1639. 10430588 10.1093/genetics/152.4.1631 PMC1460682

[61] Hsiao HY, Jukam D, Johnston R, Desplan C (2013) The neuronal transcription factor erect wing regulates specification and maintenance of *Drosophila* R8 photoreceptor subtypes. Dev Biol 381: 482–490. 23850772 10.1016/j.ydbio.2013.07.001 PMC3757101

[62] Maccioni HJ (2007) Glycosylation of glycolipids in the Golgi complex. J Neurochem 103 Suppl 1: 81–90. 17986143 10.1111/j.1471-4159.2007.04717.x

[63] Varki A (1998) Factors controlling the glycosylation potential of the Golgi apparatus. Trends Cell Biol 8: 34–40. 9695806 10.1016/s0962-8924(97)01198-7

[64] Altmann F, Schwihla H, Staudacher E, Glossl J, Marz L (1995) Insect cells contain an unusual, membrane-bound beta-N-acetylglucosaminidase probably involved in the processing of protein N-glycans. J Biol Chem 270: 17344–17349. 7615537 10.1074/jbc.270.29.17344

[65] Boquet I, Hitier R, Dumas M, Chaminade M, Preat T (2000) Central brain postembryonic development in *Drosophila*: implication of genes expressed at the interhemispheric junction. J Neurobiol 42: 33–48. 10623899

[66] Liao YF, Lal A, Moremen KW (1996) Cloning, expression, purification, and characterization of the human broad specificity lysosomal acid alpha-mannosidase. J Biol Chem 271: 28348–28358. 8910458 10.1074/jbc.271.45.28348

[67] Merkle RK, Zhang Y, Ruest PJ, Lal A, Liao YF, et al (1997) Cloning, expression, purification, and characterization of the murine lysosomal acid alpha-mannosidase. Biochim Biophys Acta 1336: 132–146. 9305783 10.1016/s0304-4165(97)00023-8

[68] Wakamatsu N, Gotoda Y, Saito S, Kawai H (1997) Characterization of the human MANB gene encoding lysosomal alpha-D-mannosidase. Gene 198: 351–357. 9370301 10.1016/s0378-1119(97)00337-5

[69] Daniel PF, Evans JE, De Gasperi R, Winchester B, Warren CD (1992) A human lysosomal alpha(1→6)-mannosidase active on the branched trimannosyl core of complex glycans. Glycobiology 2: 327–336. 1421754 10.1093/glycob/2.4.327

[70] De Gasperi R, Daniel PF, Warren CD (1992) A human lysosomal alpha-mannosidase specific for the core of complex glycans. J Biol Chem 267: 9706–9712. 1577805

[71] Park C, Meng L, Stanton LH, Collins RE, Mast SW, et al (2005) Characterization of a human core-specific lysosomal alpha 1,6-mannosidase involved in N-glycan catabolism. J Biol Chem 280: 37204–37216. 16115860 10.1074/jbc.M508930200 PMC1351102

[72] Alkhayat AH, Kraemer SA, Leipprandt JR, Macek M, Kleijer WJ, et al (1998) Human beta-mannosidase cDNA characterization and first identification of a mutation associated with human beta-mannosidosis. Hum Mol Genet 7: 75–83. 9384606 10.1093/hmg/7.1.75

[73] Winchester B (2005) Lysosomal metabolism of glycoproteins. Glycobiology 15: 1R–15R. 15647514 10.1093/glycob/cwi041

[74] Robinson D, Stirling JL (1968) N-Acetyl-beta-glucosaminidases in human spleen. Biochem J 107: 321–327. 5650361 10.1042/bj1070321 PMC1198666

[75] Myerowitz R, Proia RL (1984) cDNA clone for the alpha-chain of human beta-hexosaminidase: deficiency of alpha-chain mRNA in Ashkenazi Tay-Sachs fibroblasts. Proc Natl Acad Sci U S A 81: 5394–5398. 6236461 10.1073/pnas.81.17.5394 PMC391710

[76] O'Dowd BF, Quan F, Willard HF, Lamhonwah AM, Korneluk RG, et al (1985) Isolation of cDNA clones coding for the beta subunit of human beta-hexosaminidase. Proc Natl Acad Sci U S A 82: 1184–1188. 2579389 10.1073/pnas.82.4.1184 PMC397219

[77] Tulsiani DR, Abou-Haila A (2001) Mammalian sperm molecules that are potentially important in interaction with female genital tract and egg vestments. Zygote 9: 51–69. 11273033 10.1017/s096719940100106x

[78] Cattaneo F, Ogiso M, Hoshi M, Perotti ME, Pasini ME (2002) Purification and characterization of the plasma membrane glycosidases of *Drosophila melanogaster* spermatozoa. Insect Biochem Mol Biol 32: 929–941. 12110300 10.1016/s0965-1748(02)00031-0

[79] Miller DJ, Gong X, Shur BD (1993) Sperm require beta-N-acetylglucosaminidase to penetrate through the egg zona pellucida. Development 118: 1279–1289. 8269854 10.1242/dev.118.4.1279

[80] Mencarelli S, Cavalieri C, Magini A, Tancini B, Basso L, et al (2005) Identification of plasma membrane associated mature beta-hexosaminidase A, active towards GM2 ganglioside, in human fibroblasts. FEBS Lett 579: 5501–5506. 16212960 10.1016/j.febslet.2005.08.081

[81] Harrison RL, Jarvis DL (2006) Protein N-glycosylation in the baculovirus-insect cell expression system and engineering of insect cells to produce “mammalianized” recombinant glycoproteins. Adv Virus Res 68: 159–191. 16997012 10.1016/S0065-3527(06)68005-6

[82] Tomiya N, Betenbaugh MJ, Lee YC (2003) Humanization of lepidopteran insect-cell-produced glycoproteins. Acc Chem Res 36: 613–620. 12924958 10.1021/ar020202v

[83] Geisler C, Aumiller JJ, Jarvis DL (2008) A *fused lobes* gene encodes the processing beta-*N*-acetylglucosaminidase in Sf9 cells. J Biol Chem 283: 11330–11339. 18303021 10.1074/jbc.M710279200 PMC2431071

[84] Kim JH, Lingwood CA, Williams DB, Furuya W, Manolson MF, et al (1996) Dynamic measurement of the pH of the Golgi complex in living cells using retrograde transport of the verotoxin receptor. J Cell Biol 134: 1387–1399. 8830769 10.1083/jcb.134.6.1387 PMC2120998

[85] O'Tousa JE (1992) Requirement of N-linked glycosylation site in *Drosophila* rhodopsin. Visual Neuroscience 8: 385–390. 1534022 10.1017/s0952523800004910

[86] Brown G, Chen DM, Christianson JS, Lee R, Stark WS (1994) Receptor demise from alteration of glycosylation site in *Drosophila* opsin: electrophysiology, microspectrophotometry, and electron microscopy. Visual Neuroscience 11: 619–628. 8038132 10.1017/s0952523800002509

[87] Katanosaka K, Tokunaga F, Kawamura S, Ozaki K (1998) *N*-Linked glycosylation of *Drosophila* rhodopsin occurs exclusively in the amino-terminal domain and functions in rhodopsin maturation. FEBS 424: 149–154. 10.1016/s0014-5793(98)00160-4 9539140

[88] Li ZY, Jacobson SG, Milam AH (1994) Autosomal dominant retinitis pigmentosa caused by the threonine-17-methionine rhodopsin mutation: retinal histopathology and immunocytochemistry. Experimental Eye Research 58: 397–408. 7925677 10.1006/exer.1994.1032

[89] Daiger SP, Sullivan LS, Rodriguez JA (1995) Correlation of phenotype with genotype in inherited retinal degeneration. Behavioral and Brain Sciences 18: 452–467.

[90] Papermaster DS, Windle J (1995) Death at an early age. Apoptosis in inherited retinal degenerations. Investigative Ophthalmology & Visual Science 36: 977–983. 7730031

[91] Rosenbaum EE, Brehm KS, Vasiljevic E, Gajeski A, Colley NJ (2012) *Drosophila* GPI-mannosyltransferase 2 is required for GPI anchor attachment and surface expression of chaoptin. Vis Neurosci 29: 143–156. 22575127 10.1017/S0952523812000181 PMC3638865

[92] Rosenbaum EE, Brehm KS, Vasiljevic E, Liu CH, Hardie RC, et al (2011) XPORT-dependent transport of TRP and rhodopsin. Neuron 72: 602–615. 22099462 10.1016/j.neuron.2011.09.016 PMC3234208

[93] Rosenbaum EE, Hardie RC, Colley NJ (2006) Calnexin is essential for rhodopsin maturation, Ca^2+^ regulation, and photoreceptor cell survival. Neuron 49: 229–241. 16423697 10.1016/j.neuron.2005.12.011 PMC3414428

[94] Laemmli UK (1970) Cleavage of structural proteins during assembly of the head of bacteriophage T4. Nature 227: 680–685. 5432063 10.1038/227680a0

