# Supplementary figures and images for: Mutations in Four Glycosyl Hydrolases Reveal a Highly Coordinated Pathway for Rhodopsin Biosynthesis and N-Glycan Trimming in Drosophila melanogaster
Source: PLoS Genet. 2014 May 1;10(5):e1004349. doi: 10.1371/journal.pgen.1004349 (PMC4006722; doi:10.1371/journal.pgen.1004349)

Figure S1. Age and Temperature Data

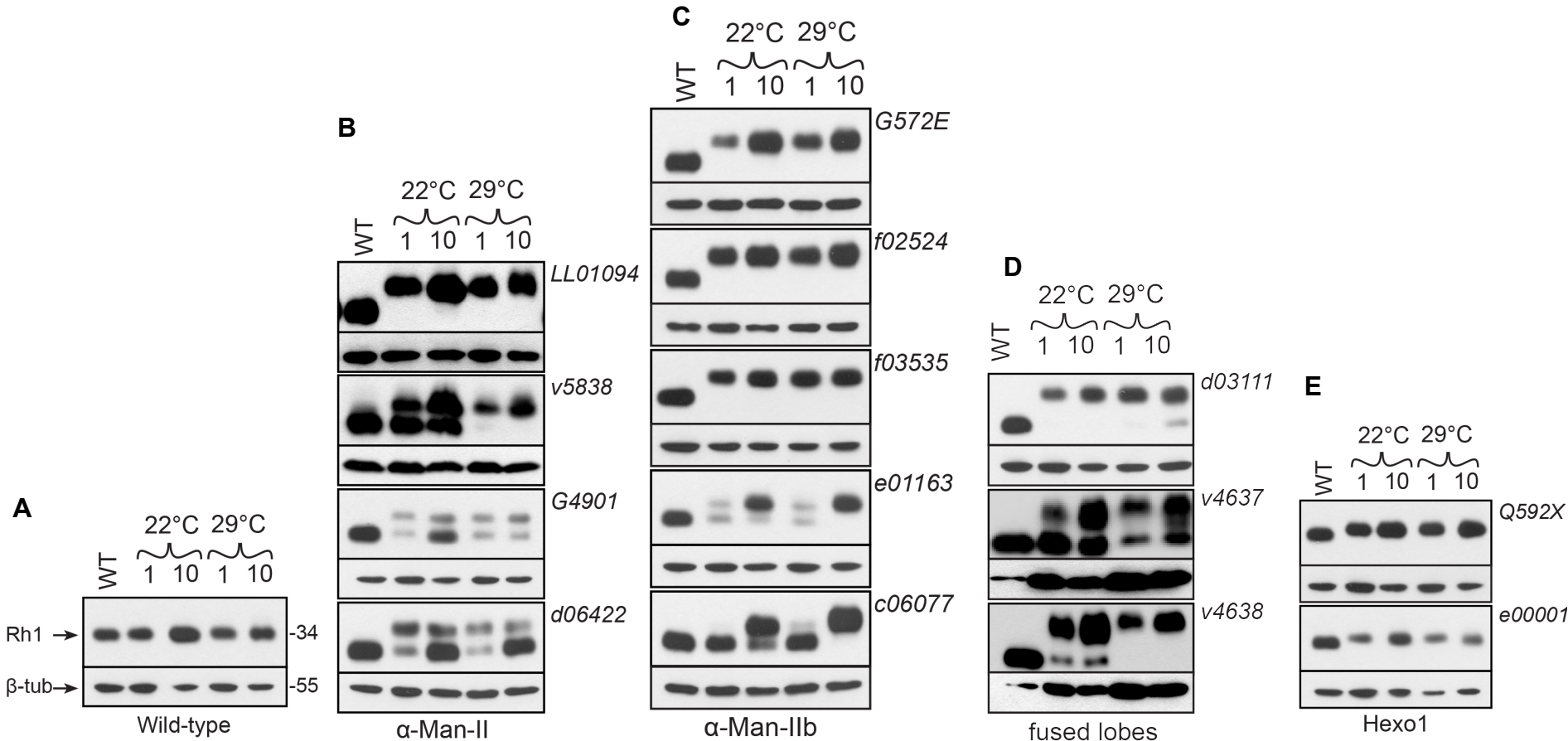

Supplement: Figure S1 — Age and temperature data. Western blots of Rh1 protein from flies aged for either 1-day or 10-days at room temperature (22°C) (Lanes 2 and 3) and 1-day or 10-days at 29°C (Lanes 4 and 5). In all cases, lane 1 is a wild-type (WT) control sample aged for 1-day at room temperature (22°C). All Rh1 blots (top) were re-probed with a monoclonal antibody directed to β-tubulin (β-tub) as a loading control (bottom), as indicated in A. (A) Wild-type (Canton S.), (B) α-man-II mutant alleles from top to bottom: α-man-IILL01094, α-man-IIv5838, α-man-IIG4901, and α-man-IId06422, (C) α-man-IIb mutant alleles from top to bottom: α-man-IIbG572E, α-man-IIbf02524, α-man-IIbf03535, α-man-IIbe01163, and α-man-IIbc06077, (D) fused lobes mutant alleles from top to bottom: fdld03111, fdlv4637, fdlv4638, and (E) hexo1 mutant alleles from top to bottom: hexo1Q592X and hexo1e00001. (PDF) [file pgen.1004349.s001.pdf]
