# Supplementary material for: Mutations in Four Glycosyl Hydrolases Reveal a Highly Coordinated Pathway for Rhodopsin Biosynthesis and N-Glycan Trimming in Drosophila melanogaster
Source: PLoS Genet. 2014 May 1;10(5):e1004349. doi: 10.1371/journal.pgen.1004349 (PMC4006722; doi:10.1371/journal.pgen.1004349)
Supplement: Figure S2 — Glucosidase I and II. Full-length amino acid (aa) alignments between the evolutionarily related human (h) and Drosophila (d) glucosidase proteins, generated with the UniProt Align program using the GenBank sequence accession numbers listed in Figure 2. Identical amino acids are marked with asterisks (*), strongly similar amino acids are marked with two dots (:), and weakly similar amino acids are marked with one dot (.). Underlined regions represent predicted transmembrane domains (TMHMM Server v.2.0). The glucosidase I enzyme is encoded by a single locus in humans, GCS1. In contrast, human glucosidase II consists of both an α- and β-subunit, encoded by GANAB and PRKCSH, respectively. In Drosophila, we have identified CG1597, CG14476, and CG6453 as the homologs for each of the glucosidase components characterized in humans, respectively (Figure 2). These Drosophila genes have not been previously characterized or annotated (FlyBase: [S1]) and here, we show that they display clear homology to the known enzymes in humans. (A) Human glucosidase I (GCS1) and the Drosophila homolog (CG1597) share 41% overall aa identity and 43% aa identity within the GH Family 63 domain (Drosophila aa62–849). (B) The α-subunit of human glucosidase II (GANAB) and the Drosophila homolog (CG14476) share 47% overall aa identity, 53% identity within the GH Family 31 carbohydrate transport domain (Drosophila aa108–891), 58% aa identity within the GH Family 31 general domain (Drosophila aa362–699), and 62% identity within the galactose mutarotase-like domain (Drosophila aa236–304). Yellow shading indicates the putative catalytic sites and purple shading indicates additional putative active sites [S2–4]. (C) The β-subunit of human glucosidase II (PRKCSH) and the Drosophila homolog (CG6453) share 39% overall aa identity, 49% aa identity within the PRKCSH-like domain (Drosophila aa26–159), and 64% aa identity within the Low Density Lipoprotein Receptor Class A domain (Drosophila aa85–120). Blue sha [file pgen.1004349.s002.pdf]

Figure S2. Glucosidase I and II

A

```
1   MARGERRRR-AVPA---EGVRTAERAA----RGGPGRRDGRGGGPRSTAGG-VALA---VVVLSLALGMSGRWV-LAWYRARRAVTLHSAPPVLPADSSSPAVAPDLFWGTYRPHVYFGM 107 h GCS1
1   MARNTGSSSGSTPAASSSGSSAAAAAAAAAAAAAASNAKQKQRSPRFSLSSNLILDKWKTMIGCVCLAIASYFGYLGYLETRVNTPFDHQKM----VVHAGLEQPDRYWGSYRPLTYFGM 116 d CG1597
    ***       :.**   .*  :*  **     .. .. . :  .** : .. : *    .:: .:.*.::. :  *.: .:*  . :.              :    ** :**:*** .****
108 KTRSPKPLLTGLMWAQQ-GTTPGTPKLRHTCEQGDGVGPYGWEFHDGLSFGRQHIQDGALRLTTEFVKRPGG-QHGGDWSWRVTVEPQDSGTSALPLVSLFFYVVTDGKEVLLPEVGAKG 225 h GCS1
117 KTRDPHSLVMGLMWYTPSNLGPGGQGIRHWCEQGDKLDSYGWTHHDGRSFGVQEIQDLPFELKTSFVKYPEGKQYGGDWTARISVRNTTRAW--DKSISLIWYVALDER--------TNG 226 d CG1597
    ***.*: *: ****       **   :** ***** :  ***  .*** *** *.***  :.*.*.*** * * *:****: *::*.    .      :**::**. * :        ::*
226 QLKFISG----------HTSELGDFRFTLLPPTSPGDTAPKYGSYNVFWTSNPGLPLLTEMVKSRLNSWFQHRPPGAPPERYLGLPGSLKWEDRGPSGQGQGQFLIQQVTLKIPISIEFV 335 h GCS1
227 HIKYVSDDKSPEPGVYGEAQGLGEFQVRFHAVK--GKIL----HKSYLSTVAPSLAKLKDTLFSHFRAF-----ADKRGNRFIGLPGEIVSQNGLPSSNPEPNFIAIQITAEVDFTLDIT 335 d CG1597
    ::*::*            .:. **:*:. :   .  *.         . : *  *.*  *.: : *::.::          :*::****.:  ::  **.: : :*:  *:* :: :::::.
336 FESGSAQAGGNQALPRLAGSLLTQALESHAEGFRERFEKTFQLKEKGLSSGEQVLGQAALSGLLGGIGYFYGQGLVLPDIGVEGSEQKVDPALFPPVPLFTAVPSRSFFPRGFLWDEGFH 455 h GCS1
336 YQSTSGFSLGESIPKPPTGRAYQDSLQAKIAQFEQRFEDRFQLKQKGHSPEEVRFARNALSNLLGGIGYFYGSSRVQSV-------YTKNPVPYWKAPLYTAVPSRSFFPRGFLWDEGFH 448 d CG1597
    ::* *. : *:.     :*     ::*:::   *.:***. ****:** *  *  :.: *** **********.. *             . :*. :  .**:*****************
456 QLVVQRWDPSLTREALGHWLGLLNADGWIGREQILGDEARARVPPEFLVQRAVHANPPTLLLPVAHML-----EVGDPDDLAFLRKALPRLHAWFSWLHQSQAGPLPLSYRWRGRDPALP 570 h GCS1
449 GLLISAWDIDIELDIICHWFDLLNVEGWIPREQILGVEALAKVPEEFVTQRNSNANPPTFFLTLKKLLTSHRAELSQNGRLATLERLYPRVQAWFNWFNTTQRGEVLGTYLWRGRNATTT 568 d CG1597
     *::. ** .:  : : **: ***.:*** ****** ** *:** **:.**  .*****:* : ::*     *:.:    ** *.:   **::***.*:. :* * :  :* ****: :
571 TLLNPKTLPSGLDDYPRASHPSVTERHLDLRCWVALGARVLTRLAEHLGEAEVAAELGPLAASLEAAESLDELHWAPELGVFADFGNHTKAVQLKPRPPQGL----------------VR 674 h GCS1
569 RELNPKSLSSGLDDYPRASHPTDKERHLDLRCWIALAAGVMAELSTLLGKDDAKY--YETASYLTDNVRLNRLHLAPYSEQYADWGLHTDQVKLKRPPSMAPQQQQRHHHQHYQLPEMQR 686 d CG1597
      ****:* ************: .*********:**.* *::.*:  **: :.       *: *     *:.** **    :**:* **. *:**  *  .                   *
675 VVGRPQPQLQYV-DALGYVSLFPLLLRLLDPTSSRLGPLLDILADSRHLWSPFGLRRSLAASSSFYGQRNSEHDPPYWRGAVWLNVNYLALGALHHYGHLEGPHQARAAKLHGELRANVVG 793 h GCS1
687 VTG-ELPTYQFVDSAFGYVSLFPLLLEQLDHDSPYLTKLLNDLRDPEQLWTNYGLRSLSKRSPLYMQRNTEHDPPYWRGPIWININYLAAKALHHYGKIDGPNAALARQIYAELRDNLVG 805 d CG1597
    *.*   *   *:* .*:**********. **  *  *   **: * * .:**: :*****:  * :* ***:********* :*:*:****  ******:::**. * * :::.*** *:**
794 NVWRQYQATGFLWEQYSDRDGRGMGCRPFHGWTSLVLLAMAEDY 837 h GCS1
806 NILRQYKRSGYLWEQYDDTSGEGKGCNPFTGWTATVVLLMAEQF 849 d CG1597
    *: ***: :*:*****.* .*.* **.** ***: *:* ***::
```

B

```
1   MAAVAAVAARRRRSWASLVLAFLGVCLGITLAVDRSNFKTCEESSFCKRQRSIRPGLSPYRALLDSLQLGPDSLTVHLIHEVTKVLLVLELQGLQKNMTRFRIDELEPRRPRYRVPDVLV 120 h GANAB
1   ------------MRFALATVAIIWALFVLADGVDPGNFKTCEQSSFCRRSRKIQGSGSKYALIPGTLNTYADSLTADLVNKENHHQFAFKLEALVGSTFRLQIDEKQPLRPRYRVEHALK 108 d CG14476
                :*  .:*:: . : :: .** .******:****:*.*.*: . * *  :  :*:   ****..*:.: .:  :.::*:.*  .  *::*** :* ******  ..*
121 ADPPIARLSVSGRDENSVELTMAEGPYKIILTARPFRLDLLEDRSLLLSVNARGLLEFEHQRAPRVSQGSKDPAEGDGAQPEETPRDGDKPEETQGKAEKDEPGAWEETFKTHSDSKPYG 240 h GANAB
109 GPTQAGRIRVQRETDG--EIVITSEKNKAVIHGDPFRIDFFENDVLVVSVNAKNWLYFEHLRQKAQEPT-SHPAENENQQEQD-----AAVETPKAADTIDDPGAWEENFKSHHDSKPYG 220 d CG14476
    .    .*: *. .    *:.::.    * :: . ***:*::*:   *::****:  * *** *.    .  ..*** * :  * ::         *  :.   *:******.**:* ******
241 PMSVGLDFSLPGMEHVYGIPEHADNLRLKVTEGGEPYRLYNLDVFQYELYNPMALYGSVPVLLAHNPHRDLGIFWLNAAETWVDISSNTAGKTLFGKMMDYLQGSGETPQTDVRWMSETG 360 h GANAB
221 PEAVALDFSFPAAKVLFGIPEHADSFILKSTSGTDPYRLYNLDVFEYVVDSKMALYGSVPVIYGHGAQRTAGVYWQNAAETWVDIQTSE--TNVVSSLVNFVSGSQKTPPPAAHFMSESG 338 d CG14476
    * :*.****:*. : ::*******.: ** *.* :**********:* : . *********: .*  :*  *::* *********.:.    ..:...:::::.** :**    .::***:*
361 IIDVFLLLGPSISDVFRQYASLTGTQALPPLFSLGYHQSRWNYRDEADVLEVDQGFDDHNLPCDVIWLDIEHADGKRYFTWDPSRFPQPRTMLERLASKRRKLVAIVDPHIKVDSGYRVH 480 h GANAB
339 IVDAFIMLGPKPMDTFKQYAALTGTHELPQLFALAYHQSRWNYNDERDVTSVSAKFDEYNIPMDTMWLDIEHTDGKRYFTWDKFKFPHPLAMIKNLTELGRHLVVIVDPHIKRDNNYFFH 458 d CG14476
    *:*.*::***.  *.*:***:****: ** **:*.:****** ** **:*.*******.** ** .*.  **::*:* *.:******:********  :**:* :*::.*:.  *:**.******* *. * .*
481 EELRNLGLYVKTRDGSDYEGWCWPGSAGYPDFTNPTMRAWWANMFSYDNYEGSAPNLFVWNDMNEPSVFNGPEVTMLKDAQHYGGWEHRDVHNIYGLYVHMATADGLRQRSGGMERPFVL 600 h GANAB
459 RDCTDRGYYVKTREGNDYEGWCWPGAASYPDFFNPVVREYYASQYALDKFQTVTADVMLWNDMNEPSVFNGPEITAPKDLIHYGNWEHRDVHNLYGHMHLMGSFAGLQQRD-PNQRPFIL 577 d CG14476
    .:   : * *****:*.*********:*.**** **.:* ::*. :: *::: : ::::************:*  **   *** ********:**    *.:  **:**.   :***:*
601 ARAFFAGSQRFGAVWTGDNTAEWDHLKISIPMCLSLGLVGLSFCGADVGGFFKNPEPELLVRWYQMGAYQPFFRAHAHLDTGRREPWLLPSQHNDIIRDALGQRYSLLPFWYTLLYQAHR 720 h GANAB
578 TRAHFAGSQRYAAIWTGDNFADWSHLQHSVKMCLTEAVAGFSFCGADVGAFGNPDTELLERWYQTGAFLPFFRAHAHIDTKRREPWLFPERTRQVIQNAVIKRYSYLPLWYTAFYELEL 697 d CG14476
    :**.******:.*:***** *:*.**: *: ***: .:.*:******** .** :**: *** ****  **: ********:** ******:*.: .::*::*: :*** **:*** :*: .
698 TGEPVIRPLLAQYPLDKEAFGVDNQLLVQDRLLVRPVMQQGVSKVDVYFPAIDDKKNGDWWYDVDTYQRQERSGYVSVPVDDFKIPVWQRGGSIVPKKERQRRASTLMLHDPYTLIICLD 817 h GANAB
721 EGIPVMRPLWVQYPQDVTTFNIDDQYLLGDALLVHPVSDSGAHGVQVYLPG-----QGEVWYDIQSYQKHHGPQTLYLPVTLSSIPVFQRGGTIVPRWMRVRRSSECMKDDPITLFVALS 835 d CG14476
     * **:*** .*** *  :* :*:* *: * ***:** :.*.  *:**:*.      :*: ***:::**::.     : :**   .***:****:***:  * **:*  * .** **::.*.
836 PQGTAQGELFLDDGHTFNYQTRQEFLLRRFSFSGNTLVSSSADPEGHFETPIWIERVVIIGAGKPAAVVLQTKGSPESRLSFQHDPETSVLVLRKPGINVASDWSIHLR-- 944 h GANAB
818 RQGKASGSLYLDDEKSYAYRQGQRIHV-NYEFAHDQLVNSF-VGKPKYKTLAWIERIVIAGLEKVPSSASITVNGVSQQLEVLH--HDNTVVVRKPGVKMDVDFAIKLNFA 924 d CG14476
      **.*.*.*:*** ::: *:   *.: : .:.*: : **.*    : :::*  ****:***  : .  *  . ..:*.. *  . ..:*:****::  *::*:*.
```

C

```
1   ---------MLLPLLLLL---PMCWAVEVKRPRGVSLTNHHFYDE---SKPFTCLDGSATIPFDQVNDDYCDCKDGSDEPGTAACPNGSFHCTNTGYKPLYIPSNRVNDGVCDCCDGTDE 105 h PRKCSH
1   MQSLQLSQVLLIPLLLALVATNVGSASEVPRPLGVPLAKASLYQPRAGENSWTCLDGSRTIPFSHINDDYCDCADGSDEPGTAACPQGQFHCVNKGHQPVNIPSSQVQDGICDCCDGSDE 120 d CG6453
             :*:**** *    :  * ** ** ** *::  :*:    .: :****** ****.::******* ************:*.***.*.*::*: ***.:*:**:******:**
106 YNSGVICENTCKEKGRKERESLQQMAEVTREGFRLKKILIEDWKKAREEKQKKLIELQAGKKSLEDQVEMLRTVKEEAEKPEREAKEQHQKLWEEQL---------AAAKAQQEQELAAD 216 h PRKCSH
121 SE-TVGCPNTCLELGAAAAVQRRNAAELHKRGAERRQEMITRGKQMRAEREARRLELDQRRKE----QELLRAEKEQLKQTAEALEAEAIEIFKEQQREVDADTAQAEREPQQMRQEATL 235 d CG6453
     :  * * *** * *     . :: **: :.* . :: :*   *: * *:: : :**:  :*.     *:**: **: ::  .  : :  ::::**          *  : ** :: *:
217 AFKELDDDMDGTVSVTELQTHPELDTDGDGALSEAEAQALLSGDTQTDATSFYDRVWAAIRDKYRSEALPTDLPAPSAPDLTEP-----KEEQPPVPSSPTEEEEEEEEEEEEEEAEEEEE 331 h PRKCSH
236 TFVRYDTNKDGFVEVTELMVDMNLDRDRNGVVTVEEAKYFLDERERVDLDAFVTLAWPRIKSLQML---AEGLFQPPQPEEVAQQPEVTTESIQPTPPKLSEEQAELAGDDDIEADEEGE 352 d CG6453
    :* . * : ** *.**** .. :** * :*.::  **: :*.   :.*  :*   .*  *:.          *  *  *: .       .*.  *.* . :**: *   ::: **:** *
332 EEDSEEAPPPLSPPQPASPAEEDKMPPYDEQTQAFIDAAQEARNKFEEAERSLKDMEESIRNLEQEISFDFGPNGEFAYLYSQCYELTTNEYVYRLCPFKLVSQKPKLGGSPTSLGTWGS 451 h PRKCSH
353 EDQYDDEEPDVGVGEASPDAEEATPPNYDPETQRLIQQANEARNALEEVERSLREIQQEVNEIDDQNNKGYGLTEEWAVHDGQCYNFEDREYVYTLCPFDRASQKSRSGGPETTLGRWDK 472 d CG6453
    *:: ::  * :.  : :  *** . * ** :** :*: *:**** :**.****::::.:.:::: .  :* . *:*   .***::  .**** ****. .*** : **  *:** * .
452 WIGPDHDKFSAMKYEQGTGCWQGPNRSTTVRLLCGKETMVTSTTEPSRCEYLMELMTPAACPEPPPEAPTEDDHDEL 528 h PRKCSH
473 WSGEP-KQYSQQKYTNGAACWNGPNRSAIINISCALEPKITAVSEPNRCEYYFEFETPAACDSEAFQSESENLHDEL 548 d CG6453
    * *   .::*  ** :*:.**:*****: :.: *. *  :*:.:**.**** :*: ***** .   :: :*: ****
```

Supporting References

S1. McQuilton P, St Pierre SE, Thurmond J (2012) FlyBase 101--the basics of navigating FlyBase. Nucleic Acids Res 40: D706-714.

S2. Kim YW, Lovering AL, Chen H, Kantner T, McIntosh LP, et al. (2006) Expanding the thioglycoligase strategy to the synthesis of alpha-linked thioglycosides allows structural investigation of the parent enzyme/substrate complex. J Am Chem Soc 128: 2202-2203.

S3. Lovering AL, Lee SS, Kim YW, Withers SG, Strynadka NC (2005) Mechanistic and structural analysis of a family 31 alpha-glycosidase and its glycosyl-enzyme intermediate. J Biol Chem 280: 2105-2115.

S4. Sim L, Quezada-Calvillo R, Sterchi EE, Nichols BL, Rose DR (2008) Human intestinal maltase-glucoamylase: crystal structure of the N-terminal catalytic subunit and basis of inhibition and substrate specificity. J Mol Biol 375: 782-792.

S5. Huang W, Dolmer K, Gettins PG (1999) NMR solution structure of complement-like repeat CR8 from the low density lipoprotein receptor-related protein. J Biol Chem 274: 14130-14136.

S6. Dolmer K, Huang W, Gettins PG (2000) NMR solution structure of complement-like repeat CR3 from the low density lipoprotein receptor-related protein. Evidence for specific binding to the receptor binding domain of human alpha(2)-macroglobulin. J Biol Chem 275: 3264-3269.

S7. Gomord V, Denmat LA, Fitchette-Laine AC, Satiat-Jeunemaitre B, Hawes C, et al. (1997) The C-terminal HDEL sequence is sufficient for retention of secretory proteins in the endoplasmic reticulum (ER) but promotes vacuolar targeting of proteins that escape the ER. Plant J 11: 313-325.
