# Supplementary material for: Mutations in Four Glycosyl Hydrolases Reveal a Highly Coordinated Pathway for Rhodopsin Biosynthesis and N-Glycan Trimming in Drosophila melanogaster
Source: PLoS Genet. 2014 May 1;10(5):e1004349. doi: 10.1371/journal.pgen.1004349 (PMC4006722; doi:10.1371/journal.pgen.1004349)
Supplement: Figure S3 — Class I α1,2-mannosidases (GH Family 47). There are seven members of GH Family 47 in humans, divided into three major subcategories based on their cellular localization and biochemical properties, including the ER subfamily (Subgroup A, orange), the Golgi subfamily (Subgroup B, yellow), and the Edem subfamily (Subgroup C, green). We have identified five GH Family 47 mannosidases in Drosophila. Amino acid sequence analysis revealed significant homology between specific human and Drosophila enzymes in GH Family 47, allowing us to assign the Drosophila proteins to specific subfamilies, as outlined below and in Figure 2. (A) Phylogenetic tree depicting the predicted evolutionary relationships between the Class I α-mannosidases from GH Family 47 in humans (h) and Drosophila (d) generated with the UniProt Align program using the GenBank sequence accession numbers listed in Figure 2. Black arrows designate speciation of the last common ancestor between humans and flies, leading to the production of orthologs. White arrows denote presumed gene duplication events, leading to the production of paralogs. According to these data, multiple gene duplication events within the Golgi subfamily (Subgroup B, yellow) likely occurred late in evolution, independently giving rise to both the mammalian gene family [S8] and the Drosophila gene pair [S9]. Therefore, in the last common ancestor between Drosophila and humans (see dotted line), there was likely only a single Golgi α1,2-mannosidase gene (yellow). This hypothesis is further supported for the human loci by the similar positioning of intron/exon boundaries [S10]. The particularly high overall aa identity (60%) and overlap in enzymatic specificity between human Golgi MAN IA and Golgi MAN IB suggests that they represent one of the most recent gene duplications in the α-mannosidase family [S8]. As with the human loci, it has been proposed that a duplication event, occurring at some point during evolution of the Drosophila lineage, gav [file pgen.1004349.s003.pdf]

Figure S3. Class I α1,2-mannosidases (GH Family 47)

A

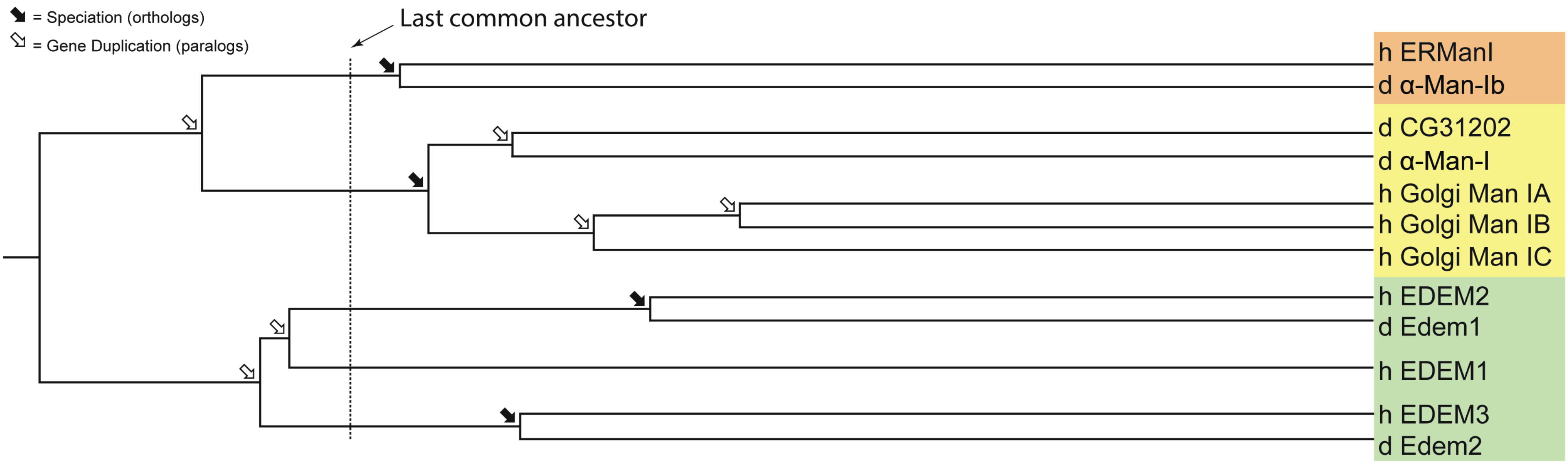

B

h ERManI	459	GARADSYYEYLLKQW	473
d α-Man-Ib	444	GARGDSYYEYLLKQW	458
h Golgi Man IA	409	GGLGDSFYEYLLKAW	423
h Golgi Man IB	395	GGLGDSFYEYLLKAW	409
h Golgi Man IC	386	GGLGDSFYEYLIKSW	400
d α-Man-I	416	GALGDSYYEYLLKAW	430
d CG31202	285	HRLYD----TLLKSW	295
h EDEM1	366	GAGLDSFYEYLLKSY	380
h EDEM2	254	GAGVDSYFEYLVKGA	268
h EDEM3	289	GAGIDSYYEYLLKAY	303
d Edem1	260	GAGVDSLFEYLVKAS	274
d Edem2	285	GAGIDSYYEYLFKSY	299

C

```
1   ------------------------------------------------------------------------------------------------------------------------   0 y MNS1
1   MAACEGRRSGALGSSQSDFLTPPVGGAPWAVATTVVMYPPPPPPPHRDFISVTLSFGESYDNSKSWRRRSCWRKWKQLSRLQRNMILFLLAFLLFCGLLFYINLADHWKALAFRLEEEQK 120 h ERManI
1   -------------------------------------------MKDGGHITLTLPPGSA-ATTDERGRKSLRRAWNQLPRCQRNLIILGI--TGFCVTVLLCLSGDQLLAASLKD---VD  71 d α-Man-Ib

1   --------------------------------------------------------------------------------------------------------------------MKNS   4 y MNS1
121 MRPEIAGL----------------KPANPPVLPAPQKADTD-----PENLPEI---SSQKTQRHIQRGP--------P-----HLQIRPPSQDLKDGT-QEEATKRQ-----EAPVDPRP 197 h ERManI
72  TKAEVAAYQMPLPNPHHHPGVDKDVPKDTAAVPVPADKRAPRVEAVSDKFNELVAYGEANSAENVARLPETVAAEGLPQASAMAVALPPPDSNKEQDIGGIDILNNVEEPVAQDPAEPLS 191 d α-Man-Ib

5   VGISIATIV-------------AIIAAIYYVPWYEHFERK---------SPG-AGEMRDRIESMFLESWRDYSKHGWGYDVYGPIEHTSHNMPRGNQPLGWIIVDSVDTLMLMYNSSTLY 101 y MNS1
198 EGDPQRTVISWRGAVIEPEQGTELPSRRAEVPTK---PPLPPARTQG--TPVHLNYRQKGVIDVFLHAWKGYRKFAWGHDELKPVSRSFSEW----FGLGLTLIDALDTMWIL------- 301 h ERManI
192 EGEQLEQAI-------------KNPLTLGGVSFKEHLKKIKPILAKGQHFHGATNERQSAVVAAFKHSWAGYKKYAWGHDNLKPISQYSHEW----FGLGLTIVDSLDTMYIM------- 287 d α-Man-Ib
     *      :                     *                          :. :   * .:*  * *..**:*   *:.:   :       **  ::*::**: ::
102 KSEFEAEIQRSEHWINDVLDFDIDAEVNVFETTIRMLGGLLSAYHLSDVLEVGNKTVYLNKAIDLGDRLALAFLSTQTGIPYSSINLHSGQAVKNHADGGASSTAEFTTLQMEFKYLAYL 221 y MNS1
302 --GLRKEFEEARKWVSKKLHFEKDVDVNLFESTIRILGGLLSAYHLSG------DSLFLRKAEDFGNRLMPAFRT-PSKIPYSDVNIGTGVAHPPR-WTSDSTVAEVTSIQLEFRELSRL 411 h ERManI
288 --GLDDEFKEGRDWVEQSLRFDTKRDVNLFEVTIRVLGGLLSAYHLSG------DTMFLAKAAELGNRLLPAFQS-PSNIPYSDVNLGDLSAHSPK-WSPDSSTSEVTTIQLEFRDLSRS 397 d α-Man-Ib
       :  *::....*:.. * *: . :**:** ***:***********       .:::* ** ::*:**  ** :  : ****.:*:    *   :     *:.:*.*::*:**: *:
222 TGNRTYWELVERVYEPLYKNNDLLNTYDGLVPIYTFPDTGKFGA-STIRFGSRGDSFYEYLLKQYLLTHET---LYYDLYRKSMEGMKKHLLAQSKPSSLWYIGEREQGLHGQLSPKMDH 337 y MNS1
412 TGDKKFQEAVEKVTQHI---HGLSGKKDGLVPMFINTHSGLFTHLGVFTLGARADSYYEYLLKQWIQGGKQE-TQLLEDYVEAIEGVRTHLLRHSEPSKLTFVGELAHG---RFSAKMDH 524 h ERManI
398 TNISIYEQVAHKVNEKV---HDLE-KNHGLVPIFINANTGTFRNYATISLGARGDSYYEYLLKQWIQTGRKDNDNLILDYMQAVDGVLTQLMRRTPREHWVYIGELINGK--DFKPKMDH 511 d α-Man-Ib
    *    : : ..:* : :   . *  . .****::   .:* *   ..: :*:*.**:*******::   .         * ::::*: .:*: ::  .   ::**  .*    :. ****
338 LVCFMGGLLASGSTEGLSIHEARRRPFFSLSLERKSDWDLAKGITDTCYQMYKQSSSGLAPEIVVFNDGNIKQDGWWRSSVGDFFVKPLDRHNLQRPETVESIMFMYHLSHDHKYREWGA 457 y MNS1
525 LVCFLPGTLALGVYHGLPA----------------SHMELAQELMETCYQMNRQMETGLSPEIVHFNLYPQP-------GRRDVEVKPADRHNLLRPETVESLFYLYRVTGDRKYQDWGW 621 h ERManI
512 LTCYLPGTLILGHQNGMPD----------------SHLILARDLLDTCYQTYMMNPTHLAAEISYFALT-EK-------DDQDIYVKPNDAHNLLRPEFVESLYYFYSITGNRTYQDMGW 607 d α-Man-Ib
    *.*:: * *  *  .*:                  *.  **: : :****      : *: **  *                *. *** * *** *** ***: ::* :: ::.*:: *
458 EIATSFFENTCVDCNDPKLRRFTSLSDCI-TLPTKKSNNMESFWLAETLKYLYILFLDE---FDLTKVVFNTEAHPFPVLDEEILKSQSLTTGWSL 549 y MNS1
622 EILQSFSRFTRVPSG-----GYSSINNVQDPQKPEPRDKMESFFLGETLKYLFLLFSDDPNLLSLDAYVFNTEAHPLPIWTPA------------- 699 h ERManI
608 KIFQAFETHAKV-NA-----GYTSMGNVKNTQSTRLRDLMESFWMSETLKYFYLLFSDDRKEIDLEQWVFNSEGHPLPVYPLKT------------ 685 d α-Man-Ib
    :*  :*   : *         ::*: :       .  : ****::.*****:::** *:   :.*   ***:*.**:*:
```

D

```
1    MPVGGLLPLFSSPAGGVLGGGLGGGGGRKGSGPAALRLTEKFVLLLVFSAFITLCFGAIFFLPDSSKLLSGVLFHSSPALQPAADHKPG---PGARAED-AAEGRARRREEGAP------ 110 h Golgi Man IA
1    MTTPALLPLSGRR-----IPPLNLGPPSFPHHRATLRLSEKFILLLILSAFITLCFGAFFFLPDSSKHKRFDLG-LEDVLIPHVDAGKGAKNPGVFLIH-GPDEH-RHR----------- 101 h Golgi Man IB
1    -------------------MLMRKVPGFVPASPWGLRLPQKFLFLLFLSGLVTLCFGALFLLPHSSRLKRLFLAPRT-------------QQPGLEVVA-EIAGHAPAREQEPP------  81 h Golgi Man IC
1    ----------------MYRISPIG-------RKSNFHSREKCLIGLVLVTLCFLCFGGIFLLPDNFGSDRVLRVYKH-----FRKAG-----PEIFIPAPPLAAHAPHRSEDPHFIGDRQ  87 d α-Man-I
1    ------------------------------------------------------------------------------------------------------------------------   0 d CG31202

111  ----------GDPEAA-----LEDNLAR---IRENHER-----------ALREAKETLQKLPEEIQR-DILLEK-KKV-----AQDQLRDKAPFRGLPPVDFVPPIGVESR--EPADAAI 192 h Golgi Man IA
102  ---------------EE-----EERLRNK---IRADHEK-----------ALEEAKEKLRKSREEIRA-EIQTEK-NKV-----VQEMKI--KENKPLPPVPIPNLVGIRGG--DPEDNDI 177 h Golgi Man IB
82   ----------PNPAPAAPAPGEDDPSSW---A-----------------SPRRRKGGLRRTRPTGPREEATAARGNSIPASRPGDEGVPFRFDFNAFRSRLRHPVLGTRADESQEPQSQV 171 h Golgi Man IC
88   RLEQKIRAELGDMLDEPPAAGGGEPGQFQVLAQQAQAPAPVAALADQPLDQDEGHAAIPVLAAPVQGDNAASQA-SSHPQSS-----AQQHNQQQ---P-QLPLGGGGNDQAPDTLDATL 197 d α-Man-I
1    ------------------------MYFFFFVIRRAQYNW----------YTANTKSRIRIIATGIFTVGVI--V-ITYIQCR-----AMMRESFQ---K-NIPSN------DNSLKDMNP  68 d CG31202
                                                      . :  :                  .                  .                  .  :
193  REKRAKIKEMMKHAWNNYKGYAWGLNELKPISKGGHSSSLFGNI-KGATIVDALDTLFIMEMKHEFEEAKSWVEENLDF-NVNAEISVFEVNIRFVGGLLSAYYLSGEEIFRKKAVELGV 310 h Golgi Man IA
78   REKREKIKEMMKHAWDNYRTYGWGHNELRPIARKGHSPNIFGSSQMGATIVDALDTLYIMGLHDEFLDGQRWIEDNLDF-SVNSEVSVFEVNIRFIGGLLAAYYLSGEEIFKIKAVQLAE 296 h Golgi Man IB
172  RAQREKIKEMMQFAWQSYKRYAMGKNELRPLTKDGYEGNMFGGL-SGATVIDSLDTLYLMELKEEFQEAKAWVGESFHL-NVSGEASLFEVNIRYIGGLLSAFYLTGEEVFRIKAIRLGE 289 h Golgi Man IC
198  EERRQKVKEMMEHAWHNYKLYAWGKNELRPLSQRPHSASIFGSYDLGATIVDGLDTLYIMGLEKEYREGRDWIERKFSLDNISAELSVFETNIRFVGGMLTLYAFTGDPLYKEKAQHVAD 317 d α-Man-I
69   DDMRAKIKEMMMHAWRNYARVVWGTNEFRPISRRVHFGGDFATYKLGATIIESLDTLHLMGLNKELRRSRDWIEKSFHLDRVDEALSVYELTSRLLCPMLTLYSLTGDSLYMDKAIHIAD 188 d CG31202
        * *:**** .** .*      * **::*:::  :    *.    ***:::.****.:* :..*   .: *:  .: :  :.   *::* . * :  :*: : ::*: ::  ** .:.
311  KLLPAFHTPSGIPWALLNMKSGIGRNWPWASGG-SSILAEFGTLHLEFMHLSHLSGNPIFAEKVMNIRTVLNKLEKPQGLYPNYLNPSSGQWGQHHVSVGGLGDSFYEYLLKAWLMSDKT 429 h Golgi Man IA
297  KLLPAFNTPTGIPWAMVNLKSGVGRNWGWASAG-SSILAEFGTLHMEFIHLSYLTGDLTYYKKVMHIRKLLQKMDRPNGLYPNYLNPRTGRWGQYHTSVGGLGDSFYEYLLKAWLMSDKT 415 h Golgi Man IB
290  KLLPAFNTPTGIPKGVVSFKSG---NWGWATAGSSSILAEFGSLHLEFLHLTELSGNQVFAEKVRNIRKVLRKIEKPFGLYPNFLSPVSGNWVQHHVSVGGLGDSFYEYLIKSWLMSGKT 406 h Golgi Man IC
318  KLLPAFQTPTGIPYALVNTKTGVAKNYGWASGG-SSILSEFGTLHLEFAYLSDITGNPLYRERVQTIRQVLKEIEKPKGLYPNFLNPKTGKWGQLHMSLGALGDSYYEYLLKAWLQSGQT 436 d α-Man-I
189  KILPAFDTPTGIPRRLVVPKEGSTLTK--YLSD-ISRTSEFGSLHLEFYYLSEVSGYPVYRERVDAIREILAKTTRPNGLYPNAYCTKFGKWENYNCSMHRLYD----TLLKSWIQSGRT 301 d CG31202
     *:****.**:***  ::  * *   .     .   *  :***:**:** :*: ::*   : ::*  ** :* :  :* *****      *.* : . *:  * *    .*:*:*: * :*
430  DLEAKKMYFDAVQAIETHLIRKSSSGLTYIAEWKGGLLEHKMGHLTCFAGGMFALGADAAPEGMAQHYLELGAEIARTCHESYNRTFMKLGPEAFRFDGGVEAIATRQNEKYYILRPEVM 549 h Golgi Man IA
416  DHEARKMYDDAIEAIEKHLIKKSRGGLTFIGEWKNGHLEKKMGHLACFAGGMFALGADGSRADKAGHYLELGAEIARTCHESYDRTALKLGPESFKFDGAVEAVAVRQAEKYYILRPEVI 535 h Golgi Man IB
407  DMEAKNMYYEALEAIETYLLNVSPGGLTYIAEWRGGILDHKMGHLACFSGGMIALGAEDAKEEKRAHYRELAAQITKTCHESYARSDTKLGPEAFWFNSGREAVATQLSESYYILRPEVV 526 h Golgi Man IC
437  DEEAREMFDEAMLAILDKMVRTSPGGLTYVSDLKFDRLEHKMDHLACFSGGLFALGAATRQNDYTDKYMEVGKGITNTCHESYIRAPTQLGPEAFRFSEAVEARALRSQEKYYILRPETF 556 d α-Man-I
302  DTQNADTFKEAMLAVAQNLVVINPEDVTYVSTFRNGTLFHRMRHSDCFAGGLFVLGAAETQMKHWEKYAHIGIGLTDTCHDSYWSSPTRLGPDTFAFTEESQQEIEPLQRNYYNLRPEVA 421 d CG31202
     * :  . : :*: *:   ::  .   :*::.  :   * ::* *  **:**::.*** :        * .:.  :: ***:**  :  :***::* *     :       ..** ****.
550  ETYMYMWRLTHDPKYRKWAWEAVEALENHCRVNGGYSGLRDVYLLHESYDDVQQSFFLAETLKYLYLIFSDDDLLPLEHWIFNSEAHLLPILPKDKKEVEIREE------- 653 h Golgi Man IA
536  ETYWYLWRFTHDPRYRQWGWEAALAIEKYCRVNGGFSGVKDVYSSTPTHDDVQQSFFLAETLKYLYLLFSGDDLLPLDHWVFNTEAHPLPVLHLANTTLSGNPAVR----- 641 h Golgi Man IB
527  ESYMYLWRQTHNPIYREWGWEVVLALEKYCRTEAGFSGIQDVYSSTPNHDNKQQSFFLAETLKYLYLLFSEDDLLSLEDWVFNTEAHPLPVNHSDSSGRAWGRH------- 630 h Golgi Man IC
557  ESYFVLWRLTHDQKYRDWGWEAVLALEKHCRTAHGYCGLRNVYQQEPQKDDVQQSFFLAETLKYLYLLFSDDSVLPLDEWVFNTEAHPLPIKGANAYYRQAPVTLPVSNAS 667 d α-Man-I
422  ETYLVLWRITHHPQYRLWGLEMVQAIEKYCRMPYGYTGVMDVNNVTSEPDDVQGSFFLGSTLKYLYLLFSDDDVVSLEQWVFNSAGHFLPIKGVNPMYRQHNSSN------ 526 d CG31202
     *:*  :** **.  ** *. * . *:*::**   *: *: :*       *: * ****..*******:** *.:: *:.*:**: .* **:
```

E

```
1   ---MPFRLLIPLGLLCALLPQH--HGAPGP-DGSAPDPAHYRERVKAMFYHAYDSYLENAFPFDELRPLTCDGHDTWGSFSLTLIDALDTLLILGNVSEFQRVVEVLQDSVDFDIDVNAS 114 h EDEM2
1   MQQKPRNNGYPGTLICAVLLVLLSTGVSGQKQYSKARKLELREDVRRMFQHAYDGYLRHASNYDELRPLTCDGHDTWGSYSLTLIDALDTLATMGNFTEFRRAARLLEEKMDFDRDINVS 120 d Edem1
            *  .    *   *:**:*        *. *   : *      . ** *: ** ****.**.*.*     .***********************:************  :**.:**:*..:**::*** *:*.*

115 VFETNIRVVGGLLSAHLLSKKAGVEVEAGWPCSGPLLRMAEEEAARKLLPAFQTPTGMPYGTVNLLHGVNPGETPVTCTAGIGTFIVEFATLSSLTGDPVFEDVARVALMRLWESRSDIGL 234 h EDEM2
121 VFETNIRIVGGLLSAHLLSKRAGVELEPGWPCKGPLLRLAEDVARRLLPAFVTNTGMPYGTVNLRYGVPKGETSITCTAGVGTFLIEFGTLSRLTGKTIYEDVAMKAVHALWAYRSPIGL 240 d Edem1
    *******:************:****:* ****.*****:**:.**:***** * ********** :**  *** :*****:***::**.*** ***. ::****  *:  **  ** ***

235 VGNHIDVLTGKWVAQDAGIGAGVDSYFEYLVKGAILLQDKKLMAMFLEYNKAIRNYTRFDDWYLWVQMYKGTVSMPVFQSLEAYWPGLQSLIGDIDNAMRTFLNYYTVWKQFGGLPEFYN 354 h EDEM2
241 FGNHIDVQSGRWTALDSGIGAGVDSLFEYLVKASVLLNRPELLELFHEARAAIDKYMRKEDWYVWVGMNKGRVTLPVFQSLEAFWPGILSIIGDTEPALRTISRYIGVWKKYGFLPEFYN 360 d Edem1
    .****** :*:*.* *:******** ******.::**:   :*: :* *  . ** :* * :***:** * ** *::********:***: *:*** : *:**: .*  ***::* ******

355 IPQGYTVEKREGYPLRPELIESAMYLYRATGDPTLLELGRDAVESIEKISKVECGFATIKDLRDHKLDNRMESFFLAETVKYLYLLFDPTNFIHNNGSTFDTVITPYGECILGAGGYIFN 474 h EDEM2
361 IAAGEASPNREVYPLRPELIESAMYLYRATGNEYLLEFGEHMLETLEFSAKTKCGYATIRNVVTHEKENRMESFFLAETSKYLYLLFDEENFLHNDGSGGELLSTEDDVCVVQAGAYIFN 480 d Edem1
    *  * :  :** *****************:  ***:*.. :*::*  :*.:**:***:::  *: :*********** ********  **:**:**   : : *     *:: **.****

475 TEAHPIDPAALHCCQRLKEEQWEVEDLMREFYSLKRSRSKFQKNTVSSGPWEPPARPGTL--FSPENHDQARERKPAKQKVPLLSCPSQPFTSKLAL-------LGQVFLDSS------- 578 h EDEM2
481 TEAHPMDMSALHCCHAHNEDIYTSLDLQRFSPRAILERSK-KRQVAVQEQWVPQCHPENNEFFNKEDESVQA-QDSEKEQEREQSGTSTTMAVDIEVFDEFQQPAGDLLVSNFEQIREER 598 d Edem1
    *****:* :*****:  :*: :   ** *        .*** :::.. .  * * .:* .   *. *:..     :.   *::     *   *   :: .: :           *:::::..

579 ------------------------------------------------------------------------------------------------------------------------ 578 h EDEM2
599 ELNESLHRNVVARNQLTVSDLDEFFAQRRESFASASEALNYVKTFMGNYTMDVAFIRGLQLYDTNMSSVLGTGAQKEYESRMRTLWQLYELQQQYVANIRVIQGLGLLTFPADGDRIPSH 718 d Edem1

579 ------------------------------------------------------------------------------------------------------------------------ 578 h EDEM2
719 LTEVLDSLDKSAHLEHMDLQDLETRVSNTSSPQLAHIREVILKARNAYAIAMVNTSAMQEFAIRIYLSGTSEAGVRIHPLLEDGELSEEARKRPLKLAEERALFSYARRIVDFRKRMAET 838 d Edem1

579 ------------------------------------------------------------------------------------------------------------------------ 578 h EDEM2
839 VDRLQTLMQDIPPPKRKEATTDSQAADASTVYTQTKAQMAAPAQQPEPSQEEKQSTQIQKGKLAENGSGLGEEESGCVWSQLVQTILRKTTVQRVKFDEAVLQENTRKALEKYARKELPH 958 d Edem1

579 ------------------------ 578 h EDEM2
959 HLFACHRPEYIEGFAYRDFYPEAL 982 d Edem1
```

F

```
1   MSEAGGRGCGSPVPQRARWRLVAATAAFCLVSATSVW---TAGAEPMSREEKQKLGNQVLEMFDHAYGNYMEHAYPADELMPLTCRGRVRGQEPSRGDVDDALGKFSLTLIDSLDTLVVL 117 h EDEM3
1   MSTTSSE-----SFVRGQRLMLIFLVAMCIVVACVLSTETTMPTQNMSNKERAELREEARDMFYHAYNAYMQNAYPADELMPLSCKGRYRGVTPSRGDMDDILGNFSMTLVDTLDTLVLL 115 d Edem2
    ** :...         *.:  ::    .*:*:* *   :     *   :: **.:*: :* ::. :** ***   **:.**********:*:** **   *****:** **:**:**:*:*****:*

118 NKTKEFEDAVRKVLRDVNLDNDVVVSVFETNIRVLGGLLGGHSLAIMLKEKGEYMQWYNDELLQMAKQLGYKLLPAFNTTSGLPYPRINLKFGIRKPEARTGTETDTCTACAGTLILEFA 237 h EDEM3
116 GDFTEFDHAVKLVIRDVQFDSDIIVSVFETNIRMVGGLLSAHILAEYLQKHADTMHWYKGELLEMSRELGYRLLPAFNTSTGIPHARVNLRLGMKDPMLKK--SRETCTACAGTILLEFA 233 d Edem2
     .  .**:.**: *:***::*.*::**********::****..* **   *:::.:  *:**: ***:*:::***:*******::*:*: *:**::*::.*   :.   . :********::****

238 ALSRFTGATIFEEYARKALDFLWEKRQRSSNLVGVTINIHTGDWVRKDSGVGAGIDSYYEYLLKAYVLLGDDSFLERFNTHYDAIMRYISQPPLLLDVHIHKPMLNARTWMDALLAFFPG 357 h EDEM3
234 ALSRLTGDPIFEVRAHAAMDALWKLRHRGSDLMGTVLNVHSGDWVRRDSGVGAGIDSYYEYLFKSYVLLGDDKYLARFNRHYNAVMKYVSEGPMLLDVLMHRPHAKSKNFMDSLLAFWPG 353 d Edem2
    ****:**   ***   *:  *:*  **:  *:*.*:*:*..:*:*:*****:************:*:*******.:* *** **:*:*:*:*: *:****  :*:*     :::.:**:****:**

358 LQVLKGDIRPAIETHEMLYQVIKKHNFLPEAFTTDFRVHWAQHPLRPEFAESTYFLYKATGDPYYLEVGKTLIENLNKYARVPCGFAAMKDVRTGSHEDRMDSFFLAEMFKYLYLLFADK 477 h EDEM3
354 LQVLSGDLKPAVQTHEMLYQVMQMHTFIPEAFTVDFQIHWGQHPLRPEFIESTYFLYRATGDHHYLQVGKKALKTLQQHAKVSCGYAAVNDVRTGKHEDRMDSFVLSETIKYLFLLFSDP 473 d Edem2
    ****.**::**::********:: *.*:*****.**::**.*********  *******:****  :**:***. ::.*:::*:*  **:**::*****.********.*:*  :***:***:*

478 EDIIFDIEDYIFTTEAHLLPLWLSTTNQSISKKNTTSEYTELDDSNFDWTCPNTQILFPNDPLYAQSIREPLKNVVDKSCPRGIIRVEESFRSGAKPPLRARDFMATNPEHLEILKKMGV 597 h EDEM3
474 QDLIINVDEFVFTTEAHLLPLSIAQLGNATFSFRQT---DEHNVLDFMRTCPSSNRLFP------EKVRKPLRNFITGSCPRTTA----------GKRLSALDFQASNADHLRAVYDMGI 574 d Edem2
    :*:*::::::::**********  ::    ::   .  . *     *  :  :*   ***.:: ***       :.:*:**:*.:   ****                   * * ** *:* :**. : .**:

598 SLIHLK---DGRVQLVQHAIQAASSIDAEDGLRFMQEMIELSSQQQKEQQLPPRAVQIV-SHPFFGRVVLTAGPAQFGLDLSKHKETRGFVASSKPSNGCSE-LTNPEAVMGKIALIQRG 712 h EDEM3
575 TMVSVGDRSQGKVRLFHSFYNAKSHEEGEMGLQFMQEMLELTKMQSINQLAQLQAVAYATDENSQDWIALMAGPSHFSPELTGDQFVEGDVILAKPLRACDESLENAEEAKGKVLVAERG 694 d Edem2
    ::: :     :*:*:*.:     :* *    :.*  **:*****:*. *. :*     :**   .  ..        :.*  ***::*. :*: .: ..* *   :**  ..*.* * * *  . **: : :**

713 QCMFAEKARNIQNAGAIGGIVIDDNEGSSSDTAPLFQMAGDGKDTDDIKIPMLFLFSKEGSIILDAIREYE-EVEVLLSDKAKDRDPEMENEEQPSSENDSQNQSGEQISSSSQEVDLVD 831 h EDEM3
695 DCTFVSKARLAQKVGAAALIVCDNVPGSSGETQPMFAMSGDGKD--DVLIPVVFMYSMEFGKLSAVMQRRKQPLRVRVMQMVEFKRWQLAK-------EQRQNQTASVAQKPEKEL---- 801 d Edem2
    :* *..***   *:.**  . ** *:   ***.:* *:* *:*****   *:  **::*::* *  . :   .::. :   :.* : : .: :  :: :        :: ***:..   .. .:*:

832 QESSEENSLNSHPESLSLADMDNAASISPSEQTSNPTENHETTNLNGECTDLDNQLQEQSETEEDSNPNVSWGKKVQPIDSILADWNEDIEAFEMMEKDEL 932 h EDEM3
802 ---------------------------------------------------------------------------------------------------- 801 d Edem2
```

Supporting References

S8. Gonzalez DS, Jordan IK (2000) The alpha-mannosidases: phylogeny and adaptive diversification. Mol Biol Evol 17: 292-300.

S9. Dai H, Yoshimatsu TF, Long M (2006) Retrogene movement within- and between-chromosomes in the evolution of *Drosophila* genomes. Gene 385: 96-102.

S10. Tremblay LO, Herscovics A (2000) Characterization of a cDNA encoding a novel human Golgi alpha 1, 2-mannosidase (IC) involved in N-glycan biosynthesis. J Biol Chem 275: 31655-31660.

S11. Camirand A, Heysen A, Grondin B, Herscovics A (1991) Glycoprotein biosynthesis in *Saccharomyces cerevisiae*. Isolation and characterization of the gene encoding a specific processing alpha-mannosidase. J Biol Chem 266: 15120-15127.

S12. Gonzalez DS, Karaveg K, Vandersall-Nairn AS, Lal A, Moremen KW (1999) Identification, expression, and characterization of a cDNA encoding human endoplasmic reticulum mannosidase I, the enzyme that catalyzes the first mannose trimming step in mammalian Asn-linked oligosaccharide biosynthesis. J Biol Chem 274: 21375-21386.

S13. Tremblay LO, Herscovics A (1999) Cloning and expression of a specific human alpha 1,2-mannosidase that trims Man9GlcNAc2 to Man8GlcNAc2 isomer B during N-glycan biosynthesis. Glycobiology 9: 1073-1078.

S14. Bause E, Bieberich E, Rolfs A, Volker C, Schmidt B (1993) Molecular cloning and primary structure of Man9-mannosidase from human kidney. Eur J Biochem 217: 535-540.

S15. Lal A, Schutzbach JS, Forsee WT, Neame PJ, Moremen KW (1994) Isolation and expression of murine and rabbit cDNAs encoding an alpha 1,2-mannosidase involved in the processing of asparagine-linked oligosaccharides. J Biol Chem 269: 9872-9881.

S16. Herscovics A, Schneikert J, Athanassiadis A, Moremen KW (1994) Isolation of a mouse Golgi mannosidase cDNA, a member of a gene family conserved from yeast to mammals. J Biol Chem 269: 9864-9871.

S17. Tremblay LO, Campbell Dyke N, Herscovics A (1998) Molecular cloning, chromosomal mapping and tissue-specific expression of a novel human alpha1,2-mannosidase gene involved in N-glycan maturation. Glycobiology 8: 585-595.

S18. Lal A, Pang P, Kalelkar S, Romero PA, Herscovics A, et al. (1998) Substrate specificities of recombinant murine Golgi alpha1, 2-mannosidases IA and IB and comparison with endoplasmic reticulum and Golgi processing alpha1,2-mannosidases. Glycobiology 8: 981-995.

S19. Olivari S, Molinari M (2007) Glycoprotein folding and the role of EDEM1, EDEM2 and EDEM3 in degradation of folding-defective glycoproteins. FEBS Lett 581: 3658-3664.

S20. Trombetta ES (2003) The contribution of N-glycans and their processing in the endoplasmic reticulum to glycoprotein biosynthesis. Glycobiology 13: 77R-91R.

S21. Hosokawa N, Wada I, Hasegawa K, Yorihuzi T, Tremblay LO, et al. (2001) A novel ER alpha-mannosidase-like protein accelerates ER-associated degradation. EMBO Rep 2: 415-422.

S22. Mast SW, Diekman K, Karaveg K, Davis A, Sifers RN, et al. (2005) Human EDEM2, a novel homolog of family 47 glycosidases, is involved in ER-associated degradation of glycoproteins. Glycobiology 15: 421-436.

S23. Hirao K, Natsuka Y, Tamura T, Wada I, Morito D, et al. (2006) EDEM3, a soluble EDEM homolog, enhances glycoprotein endoplasmic reticulum-associated degradation and mannose trimming. J Biol Chem 281: 9650-9658.

S24. Vallee F, Lipari F, Yip P, Sleno B, Herscovics A, et al. (2000) Crystal structure of a class I alpha 1,2-mannosidase involved in N-glycan processing and endoplasmic reticulum quality control. EMBO J 19: 581-588.

S25. Vallee F, Karaveg K, Herscovics A, Moremen KW, Howell PL (2000) Structural basis for catalysis and inhibition of N-glycan processing class I alpha 1,2-mannosidases. J Biol Chem 275: 41287-41298.
S26. Tempel W, Karaveg K, Liu ZJ, Rose J, Wang BC, et al. (2004) Structure of mouse Golgi alpha-mannosidase IA reveals the molecular basis for substrate specificity among class 1 (family 47 glycosylhydrolase) alpha1,2-mannosidases. J Biol Chem 279: 29774-29786.
S27. Lobsanov YD, Vallee F, Imberty A, Yoshida T, Yip P, et al. (2002) Structure of *Penicillium citrinum* alpha 1,2-mannosidase reveals the basis for differences in specificity of the endoplasmic reticulum and Golgi class I enzymes. J Biol Chem 277: 5620-5630.
S28. Van Petegem F, Contreras H, Contreras R, Van Beeumen J (2001) *Trichoderma reesei* alpha-1,2-mannosidase: structural basis for the cleavage of four consecutive mannose residues. J Mol Biol 312: 157-165.
S29. Karaveg K, Moremen KW (2005) Energetics of substrate binding and catalysis by class 1 (glycosylhydrolase family 47) alpha-mannosidases involved in N-glycan processing and endoplasmic reticulum quality control. J Biol Chem 280: 29837-29848.
S30. Karaveg K, Siriwardena A, Tempel W, Liu ZJ, Glushka J, et al. (2005) Mechanism of class 1 (glycosylhydrolase family 47) alpha-mannosidases involved in N-glycan processing and endoplasmic reticulum quality control. J Biol Chem 280: 16197-16207.
S31. Lipari F, Herscovics A (1996) Role of the cysteine residues in the alpha1,2-mannosidase involved in N-glycan biosynthesis in *Saccharomyces cerevisiae*. The conserved Cys340 and Cys385 residues form an essential disulfide bond. J Biol Chem 271: 27615-27622.
S32. Lipari F, Herscovics A (1999) Calcium binding to the class I alpha-1,2-mannosidase from *Saccharomyces cerevisiae* occurs outside the EF hand motif. Biochemistry 38: 1111-1118.
S33. Romero PA, Vallee F, Howell PL, Herscovics A (2000) Mutation of Arg(273) to Leu alters the specificity of the yeast N-glycan processing class I alpha1,2-mannosidase. J Biol Chem 275: 11071-11074.
S34. Mulakala C, Reilly PJ (2002) Understanding protein structure-function relationships in Family 47 alpha-1,2-mannosidases through computational docking of ligands. Proteins 49: 125-134.
