# Supplementary material for: Mutations in Four Glycosyl Hydrolases Reveal a Highly Coordinated Pathway for Rhodopsin Biosynthesis and N-Glycan Trimming in Drosophila melanogaster
Source: PLoS Genet. 2014 May 1;10(5):e1004349. doi: 10.1371/journal.pgen.1004349 (PMC4006722; doi:10.1371/journal.pgen.1004349)
Supplement: Figure S4 — GlcNAc-transferase I. Full-length amino acid (aa) alignment between human (h) and Drosophila (d) GlcNAc-transferase I, generated with the UniProt Align program using the GenBank sequence accession numbers listed in Figure 2. Identical amino acids are marked with asterisks (*), strongly similar amino acids are marked with two dots (:), and weakly similar amino acids are marked with one dot (.). Underlined regions represent predicted transmembrane domains (TMHMM Server v.2.0). Following trimming to Man5GlcNAc2, the classical pathway for oligosaccharide maturation involves the action of GlcNAc-transferase I, which functions in the addition of a single β1,2-linked N-acetylglucosamine (GlcNAc) to a terminal mannose (M4) on branch A (Figure 4) [51], [52]. This modification occurs in the Golgi and is a prerequisite for the formation of complex N-glycans. The enzyme responsible for this function (also called Mgat1) is highly conserved between Drosophila (CG13431) and humans (MGAT1). Drosophila Mgat1 displays 51% overall aa identity with human MGAT1 and 62% aa identity within the GT13 GlcNAc-TI domain (Drosophila aa119–449). Purple shading indicates putative substrate binding sites [S35]. (PDF) [file pgen.1004349.s004.pdf]

Figure S4. GlcNAc-transferase I

```
1   MLKKQSAGLVLWGAILFVAWNALL--LLFFWTRPAPGRPPSVSALDGD---------PASLTREVIRLAQDA-------EVELERQRGL-LQQIGDALSSQRGRVPTAAPPAQPRVPVTP 101 h MGAT1
1   -MR-TRKVLLVIG--FLVTWTYATYYLLLRQTGIHTSRHQSLQAYKLNSQARDANMQSHHLAKNVFEFVKLKYLEKQPPSVASTPQISIIAAEISAELPEQHV-AKSATARIPTKTYLAN 115 d Mgat1
     ::     *:: *  ::*:*.     **:  *    .*  *:.* . :            *:::*:.:.:         .*    * .:   :*.  * .*:  . :*:     :. ::
102 APAVIPILVIACDRSTVRRCLDKLLHYRPSAELFPIIVSQDCGHEETAQAIASYGSAVTHIRQPDLSSIAVPPDHRKFQGYYKIARHYRWALGQVFRQFRFPAAVVVEDDLEVAPDFFEY 221 h MGAT1
116 GEPVFPVVVFACNRVSVKKCIDNLVQYRPSVEQFPIIVSQDCGDEPTKEAILSYGKQVTLIEQPDLSDITVLPKEKKFKGYYKIARHYGWALNTTFA-VGFEFVIIVEDDLNVAPDFFEY 234 d Mgat1
    .  *:*::*:**:* :*::*:*:*::****.* **********.* * :** ***. ** *.*****.*:* *..:**:********* ***   .*   . *   .::*****:********
222 FRATYPLLKADPSLWCVSAWNDNGKEQMVDASRPELLYRTDFFPGLGWLLLAELWAELEPKWPKAFWDDWMRRPEQRQGRACIRPEISRTMTFGRKGVSHGQFFDQHLKFIKLNQQFVHF 341 h MGAT1
235 FLGTHKLLKQDPSLWCVSAWNDNGKAAVVDAAQPELLYRTDFFPGLGWMLTKDLWAELSVKWPKSFWDDWIRHPAQRKDRVCIRPEISRTRTFGKIGVSNGLFFDKYLKHIKLSEDFVQF 354 d Mgat1
    * .*: *** ***************  :***::***************:*  :*****. ****:*****:*:* **: *.********* ***: ***.* ***::**.***.::**:*
342 TQLDLSYLQREAYDRDFLARVYGAPQLQVEKVRTNDRKELGEVRVQYTGRDSFKAFAKALGVMDDLKSGVPRAGYRGIVTFQFRGRRVHLAPPLTWEGYDPSWN 445 h MGAT1
355 TKINMSYLLKDNYDNTFLRRVYTYPIVTYDELRRNLIRIEGPVRIQYTTREQYKRTTKMLGLMDDFKSGVPRTAYHGIVSFYYNKRRVHLAPNANWKGYELSWS 458 d Mgat1
    *::::*** :: **. ** ***  * :  :::* *  :  * **:*** *:.:*  :* **:***:******:.*:***:* :. *******  .*:**: **.
```

Supporting References

S35. Gordon RD, Sivarajah P, Satkunarajah M, Ma D, Tarling CA, et al. (2006) X-ray crystal structures of rabbit N-acetylglucosaminyltransferase I (GnT I) in complex with donor substrate analogues. J Mol Biol 360: 67-79.
