# Supplementary material for: Mutations in Four Glycosyl Hydrolases Reveal a Highly Coordinated Pathway for Rhodopsin Biosynthesis and N-Glycan Trimming in Drosophila melanogaster
Source: PLoS Genet. 2014 May 1;10(5):e1004349. doi: 10.1371/journal.pgen.1004349 (PMC4006722; doi:10.1371/journal.pgen.1004349)
Supplement: Figure S5 — Class II α1,2-mannosidases (GH Family 38). There are five GH Family 38 members in humans, divided into three major subcategories based on their cellular localization and biochemical properties, including the Golgi subfamily (Subgroup A, blue), the lysosomal subfamily (Subgroup B, purple) and the ERManII subfamily (Subgroup C, grey). We have identified eight GH Family 38 mannosidases in Drosophila. Amino acid sequence analysis reveals significant homology between specific human and Drosophila enzymes in GH Family 38, allowing us to assign the Drosophila proteins to specific subfamilies, as outlined below and in Figure 2. (A) Phylogenetic tree depicting the predicted evolutionary relationships between the Class II α-mannosidases from GH Family 38 in humans (h) and Drosophila (d), generated with the UniProt Align program using the GenBank sequence accession numbers listed in Figure 2. Black arrows designate speciation of the last common ancestor between humans and flies, leading to the production of orthologs. White arrows denote presumed gene duplication events, leading to the production of paralogs. The Drosophila lysosomal α-mannosidase loci (purple) have undoubtedly resulted from more recent duplication events and, accordingly, display between 65–77% overall aa identity with one another. In a recent study, these lysosomal α-mannosidases were designated LManI (CG5322), LManII (CG6206), LManIII (CG9463), LManIV (CG9465), LManV (CG9466), and LManVI (CG9468) [46]. Further evidence for their recent duplication is the positioning of the corresponding loci within the genome. The loci encoding LManI and II are organized back-to-back at 31E5, whereas the loci encoding LManIII-VI are organized in tandem at 29F1. (B) Full-length amino acid (aa) alignment between GH Family 38 members from Subgroup A, including human (h) α-Man II (MAN2A1), human α-Man IIx (MAN2A2), Drosophila (d) α-Man-II (CG18802), and Drosophila α-Man-IIb (CG4606), generated with the UniProt Align program usi [file pgen.1004349.s005.pdf]

Figure S5. Class II α1,2-mannosidases (GH Family 38)

A

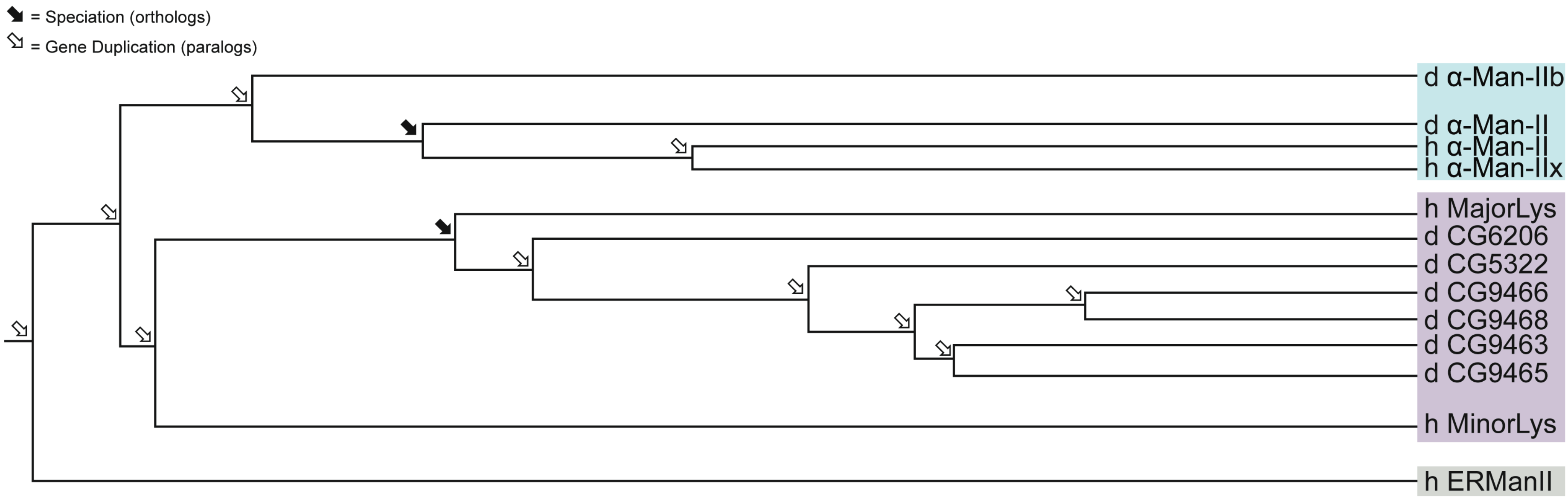

B

```
1     --MKL-SRQFTVFGS--AIFCVVIFSLYLMLDRGHLDYPRNPRREGSFPQGQLSMLQEKIDHLERLLAE--NNEIISNIRDSVINLSESVEDGPKSSQSNFSQGAGSHLLP-----SQLS  108  h α-Man II
1     --MKL-KKQVTVCGA--AIFCVAVFSLYLMLDRVQHDP-TRHQNGGNFPRSQISVLQNRIEQLEQLLEE--NHEIISHIKDSVLELTANAEGPPAMLP--YYTVNGSWVVPPEP--RPSF  108  h α-Man IIx
1     -MLRI-RRRFALVIC--SGCLLVFLSLYIILNFAAPAA---TQIK-----PNYENIENKLHELENGLQE--HGEEMRNLRARLAETSNRD--DPIRPP-----------------LKVA   86  d α-Man-II
1     MSFKLFRRGSARCIGLLSAFVTILLCLYYI-SIGQPNPTPISETG--APSAAL--RGSPLSGLTGFSVDGGSSSIIHQQKQQLVIASNQKKSSETK-------SAQTSVSPEEQSEEQYT  108  d α-Man-IIb
       :::  :  :     :     .:.** : .                   .                     . :   *     :   . : . :  :   :

109   LSVDTADCLFASQSGSHNSDVQMLDVYSLISFDNPDGGVWKQGFDITYESNEWDT--EPLQVFVVPHSHNDPGWLKTFNDYFRDKTQYIFNNMVLKLKEDSRRKFIWSEISYLSKWWDII  226  h α-Man II
109   FSISPQDCQFALGGRGQKPELQMLTVSEELPFDNVDGGVWRQGFDISYDPHDWDA--EDLQVFVVPHSHNDPGWIKTFDKYYTEQTQHILNSMVSKLQEDPRRRFLWAEVSFFAKWWDNI  226  h α-Man IIx
87    RSPRPGQCQDVVQ-DVPNVDVQMLELYDRMSFKDIDGGVWKQGWNIKYDPLKYNA-HHKLKVFVVPHSHNDPGWIQTFEEYYQHDTKHILSNALRHLHDNPEMKFIWAEISYFARFYHDL  204  d α-Man-II
109   NSNWGDKCYELIQSNTNITASEEHSKFDFQPEWMRSKEYWDRGFEERFDAQKKDKQRPPLKIIVVPHSHNDPGWLKTFTNYFQSDSRQILNLLVTKMQEYTDMTFIWSEISFLQLWWDQA  228  d α-Man-IIb
       *     .*                 :       .       .   * :*::  ::  . :     *:::***********::** .*:  .:: *:.  : ::::     *:*:*:*::  ::.

227   DIQKKDAVKSLIENGQLEIVTGGWVMPDEATPHYFALIDQLIEGHQWLENNIGVKPRSGWAIDPFGHSPTMAYLLNRAGLSHMLIQRVHYAVKKHFALHKTLEFFWRQNWDLGSVT----  342  h α-Man II
227   NVQKRAAVRRLVGNGQLEIATGGWVMPDEANSHYFALIDQLIEGHQWLERNLGATPRSGWAVDPFGYSSTMPYLLRRANLTSMLIQRVHYAIKKHFAATHSLEFMWRQTWDSDSST----  342  h α-Man IIx
205   GENKKLQMKSIVKNGQLEFVTGGWVMPDEANSHWRNVLLQLTEGQTWLKQFMNVTPTASWAIDPFGHSPTMPYILQKSGFKNMLIQRTHYSVKKELAQQRQLEFLWRQIWDNKGDT----  320  d α-Man-II
229   HPTKQRALKRLIDAGRIEITTGGWVMTDEANVHIYPMLDQLIEGHQWLKNNLNVTPKVGWSIDPFGHGSTVPYLLSGANFEGTIIQRIHYAWKQWFARQQSGDFIWKPYWRSRKDKDSEA  348  d α-Man-IIb
        *:   :: ::   *::*:.****** ***. *    :: ** **: **:. : ..*   .*::****:. *: *:*   : :    :*** **: *: :*   :   :*:*:   *       .

343   -DILCHMMPFYSYDIPHTCGPDPKICCQFDFKRLPGGRFGCPWGVPPETIHPGNVQSRARMLLDQYRKKSKLFRTKVLLAPLGDDFRYCEYTEWDLQFKNYQQLFDYMNS-QSKFKVKIQ  460  h α-Man II
343   -DIFCHMMPFYSYDVPHTCGPDPKICCQFDFKRLPGGRINCPWKVPPRAITEANVAERAALLLDQYRKKSQLFRSNVLLVPLGDDFRYDKPQEWDAQFFNYQRLFDFFNS-RPNLHVQAQ  460  h α-Man IIx
321   -ALFTHMMPFYSYDIPHTCGPDPKVCCQFDFKRMGSFGLSCPWKVPPRTISDQNVAARSDLLVDQWKKKAELYRTNVLLIPLGDDFRFKQNTEWDVQRVNYERLFEHINS-QAHFNVQAQ  438  d α-Man-II
349   GSLLTHNMPFDIYSIKGSCGPHPFICLNFDFRKIPGEYT--EYSVKAQFITDDNLESKAQLLLEQYARTASLFPHNVALIPVGDDFRYNKDREVDQQYQNYKKLIDHIMANRRLYNADIR  466  d α-Man-IIb
       :: * ***   *.:   :***.* :* :***::: .        : *   . *    *:   :: :*::*: :.:.*:   :* * *:*****: :   * * *   **::*::.: : :    ... :

461   FGTLSDFFDALDKADETQRDKGQSMFPVLSGDFFTYADR----DDHYWSGYFTSRPFYKRMDRIMESHLRAAEILYYFALRQAHKYKINK--FLSSSLYTALTEARRNLGLFQHHDAITG  574  h α-Man II
461   FGTLSDYFDALYKRTGVEPGARPPGFPVLSGDFFSYADR----EDHYWTGYYTSRPFYKSLDRVLEAHLRGAEVLYSLAAAHARRSGLAG--RYPLSDFTLLTEARRTLGLFQHHDAITG  574  h α-Man IIx
439   FGTLQEYFDAVHQAE----RAGQAEFPTLSGDFFTYADR----SDNYWSGYYTSRPYHKRMDRVLMHYVRAAEMLSAWHSWDGM-----------ARIEERLEQARRELSLFQHHDGITG  539  d α-Man-II
467   FGTPSDYFEAIKERM----KDHTPGFPTFKGDFFVYSDIFSEGRPAYWSGYFTTRPFYKLLSSELEHQLRSAEIVFTLAYNTARQAKRENAIKIYEKNYELIINARRNLGLFQHHDAITG  582  d α-Man-IIb
      *** .::*:*: :                 **.:.**** *:*          **:**:*:**::* :.  :    :*.**::                .                   : :*** *.******.***

575   TAKDWVVVDYGTRLFHSLMVLEKIIGNSAFLLILKDKLTYDSYSPDTFLEMDL--------------KQKSQDSLPQ-KNIIRL--S-AEPRYLVVYNPLEQDRISLVSVYVSSPTVQVF  676  h α-Man II
575   TAKEAVVVDYGVRLLRSLVNLKQVIIHAAHYLVLGDKETYHFDPEAPFLQVDD--------------TRLSHDALPE-RTVIQL--D-SSPRFVVLFNPLEQERFSMVSLLVNSPRVRVL  676  h α-Man IIx
540   TAKTHVVVDYEQRMQEALKACQMVMQQSVY-RLLTKPSIYSPDFSFSYFTLDD--------------SRWPGSGVEDSRTTIILGEDILPSKHVVMHNTLPHWREQLVDFYVSSPFVSVT  644  d α-Man-II
583   TSKAAVMRDYAMRLFESTQNMVKMQESCLE-LLLQKGQPRHHGFLLSEFERDNFNKLPRKMPIQMANGDESTPTVPAGEGGAGGSSSSGSVGSFVLYNSLAQKRLEVVTLRVQHPNVKIV  701  d α-Man-IIb
      *:*   *: **   *: .:          :    .      :* .                  :  *                                 :     .          .   .*:.* * : * .:* . *. * * :

677   SASGKPV-EVQVSAVWDTANT-----------------ISETAYEISFRAHIPPLGLKVYKILESASSNSHLADYV-LYKN----------------KVEDSGIFTIKNMINTEEGITLE  761  h α-Man II
677   SEEGQPL-AVQISAHWSSATE-----------------AVPDVYQVSVPVRLPALGLGVLQLQLGLDGHRTLPSSVRIYLH----GRQL--------SVSRHEAFPLRVIDSGTSDFALS  766  h α-Man IIx
645   DLANNPV-EAQVSPVWSWHHDTLTK--------TIHPQGSTTKYRIIFKARVPPMGLATYVLTISDSKPEHTSYASNLLLR----KNP---------TSLPLGQYPEDVKFGDPREISLR  742  d α-Man-II
702   NDKGVELNHIQINPVWNITDTYEQGLGTSVSGTVGRIRTSTRQFEVMFVAELEPLSLSTYRVQVDEVNFKRNI--ATIYCDDCTESQPASVSPPEEIESSPTSVFEA--RGKPAGDIQLE  817  d α-Man-IIb
      .      :     *:.    *.                                          :.: . ..:   :.* .   :                   :                        :                    : *

762   NSFVLLRF-DQTGLMKQMMTKEDGKHH----EVNVQFSWYGTTIKRDKSGAYLFLPDGNAKPYVY-----TTPPFVRVTHGRIYSEVTCFF-DHVTHRVRLYHIQGIEGQSVEVSNIVDI  870  h α-Man II
767   NRYMQVWFSGLTGLLKSIRRVDEEHEQ----QVDMQVLVYGTRTSKDKSGAYLFLPDGEAKPYVP-----KEPPVLRVTEGPFFSEVVAYY-EHIHQAVRLYNLPGVEGLSLDISSLVDI  876  h α-Man IIx
743   VGNGPTLAFSEQGLLKSIQLTQDSPHV----PVHFKFLKYGVRSHGDRSGAYLFLPNGPASPVEL-----GQ-PVVLVTKGKLESSVSVGL-PSVVH------QTIMRGGAPEIRNLVDI  845  d α-Man-II
818   NPHMRLLFDEKSGFLKTITRKNQKKELLKPLQCNIKFA--AYRSAQFHSGAYLFKTDPEQSEAEKEVLEGYTDMRIIITSGPIASDVTVIYGPFLAHTVRIFNTRTHLDAAIYVENDIDF  935  d α-Man-IIb
                              *::* :     ::   .            ..:.      .         :******    :        .                         :  :* *  :  *.*                   :       :       :    .  :*:

871   RK---VYNREIAMKISSDI----------------------KSQNRFYTDLNGYQIQPRMTLSKLPLQANVYPMTTMAYIQDAKHRLTLLSAQSLGVSSLNSGQIEVIMDRRLMQDDNRG  965  h α-Man II
877   RD---YVNKELALHIHTDI----------------------DSQGIFFTDLNGFQVQPRRYLKKLPLQANFYPMPVMAYIQDAQKRLTLHTAQALGVSSLKDGQLEVILDRRLMQDDNRG  971  h α-Man IIx
846   GS---LDNTEIVMRLETHI----------------------DSGDIFYTDLNGLQFIKRRRLDKLPLQANYYPIPSGMFIEDANTRLTLLTGQPLGGSSLASGELEIMQDRRLASDDERG  940  d α-Man-II
936   EPPPKNRETELFMRFVTNIDNIAQAPMQRDPLKEPVDAATMPELPVFYSDQNGFQYHERIKVPAIGIEGNYFPITSGAFIQDSRLRLTLLTTHAQGAASYEPGQLEVMLDRRTLYDDYRG  1055  d α-Man-IIb
                  : *: ::: :.*                               .     *::*  ** *      *     :     :   ::.*  :*:         :*:*:.  ****  :  :      *  :*       *::*::  ***         **  **

966   LEQGIQDNKITANLFRILLEKRSAVNTEEEKKSVSYPSLLSHITSSLMN-HPVIPMANKFSSPTLELQGEFS-----PLQSSLPCDIHLVNLRTIQSKVG-NGHSNEAALILHRKG-FDC  1077  h α-Man II
972   LGQGLKDNKRTCNRFRLLLERRTVGSEVQDSHSTSYPSLLSHLTSMYLN-APALALPVARMQLPGPGLRSFH-----PLASSLPCDFHLLNLRTLQAEED-TLPSAETALILHRKG-FDC  1083  h α-Man IIx
941   LGQGVLDNKPVLHIYRLVLEKVNNCVRPSELHPAGYLTSAAHKASQSLL-DPLDKFIFAENEW-IGAQGQFG-----GDHPSAREDLDVSVMRRLTKS---SAKTQRVGYVLHRTNLMQC  1050  d α-Man-II
1056  MGEGVVDSRLTRHKFWLLVEDLPGGQHVTQPPSYKVLSQQAQQLANALRYPPNLYFVSNLEQPEL--AAALLPLVRLLPKGPLPCDVHLTTLRTLSDPELQLFPSASALLVLHRQG-FDC  1172  d α-Man-IIb
      : :*: *.:  .  .  :  :::*                       :           :   ::    :     :        *        :            .                :                      *..:   :* :                    :    .    :***      ::*

1078  RFSSKGT---GLFCST---TQGKILVQKLL-NKFIVESLTPSSLSLMHSPPGTQNISEINLSPMEISTFRIQLR---  1144  h α-Man II
1084  GLEAKNL---GFNCTT---SQGKVALGSLF-HGLDVVFLQPTSLTLLYPLASPSNSTDVYLEPMEIATFRLRLG---  1150  h α-Man IIx
1051  GTPEEHT---Q-----------KLDVCHLL-PN--VARCERTTLTFLQNLEHLDGMVAPEVCPMETAAYVSSHSS--  1108  d α-Man-II
1173  SVSAGRELSMEAICPLSSKGLGNVQLGRLSLHTIEATSLTGTEQKSSRESYHIRSLAEISLEPMELRTFNLTFRSEV  1249  d α-Man-IIb
                                   :: :   *          .        :   .                         : ***    ::
```

C

```
1    MGAYARASGVCARGCLDSAGPWTMSRALRPPLPPLCF-------FLLLLAAAGARAGGYETCPTVQPNMLNVHLLPHTHDDVGWLKTVDQYFYGIKNDIQHAGVQYILDSVISALLADPT  113 h Major Lys
1    --------------------------------------------------------------------MINVHLVPHSHDDVGWLKTVDQYYYGSQNKIQHAGVQYILDTVVEELLKDSS   52 d CG5322
1    -----------------------MSAKTSVWLVFFCALGCLIHDADLRSIQPKASQCGYQSCHPTKPNMLNVHLVAHTHDDVGWLKTVDQYYYGSETKIQKAGVQYIIDSVVEALLRDPE   97 d CG6206
1    -------------------------------MSSVKNLAILLA-LFASYIVQSEAVC-YESCPETKSNMINIHLVPHSHDDLGWLKTVDQYYYGNKHNIQHAGVQYIFDTVVAELSKDSR   87 d CG9463
1    -------------------------------MTYLGRILFLSI-LLVLFAKQSEAVCGYESCPETKANMVNIHLVPHSHNDVGWLKTVDQSYYGYKNKIHHAGVQYILDSVVSQLLQDPK   88 d CG9465
1    --------------------------------MKFLGIVLCVA-FLALQAKSAQAVCGYESCHETKSNMINIHLVPHSHDDVGWLKTVDQYYYGHRNNIQHAGVQYIIDTVISELIKNPD   87 d CG9466
1    -------------------------------MKYLCGIVLFVA-LLAISVRPSEEACGYEACPKTKTNMINIHMVPHSHDDVGWLKTVDQYFYGHRSNIQHAGVQYIIDTVIAELIKDPA   88 d CG9468
                                                                         *:*:*:: *:*:*:******** :** . .*::*****:*:*:  *  :

114  RRFIYVEIAFFSRWWHQQTNATQEVVRDLVRQGRLEFANGGWVMNDEAATHYGAIVDQMTLGLRFLEDTFGNDGRPRVAWHIDPFGHSREQASLFAQMGFDGFFFGRLDYQDKWVRMQKL  233 h Major Lys
53   RRFIQVETFFFAKWYSEQTETVQRAVKKLVAQGRLEFAGGAWSMNDEATVHYQSVVDQFNLGLRYLKDTFGDCGRPTVGWQIDPFGHSREMASIFAQMAFNGEFFARMDYVDKKQRMLDL  172 d CG5322
98   KRFIYVESAFFFKWWKEQKPKVQEAVKMLVEQGRLEFIGGAWSMNDEATTHYQSVIDQFSWGLRLLNDTFGECGRPRVGWQIDPFGHSREMASMFAQMGFDGMFFGRLDYQDKDERLMTK  217 d CG6206
88   RRFIQVETGFFAKWWEDQSETKKKLVKTLVNEGRLEFTGGAWSMNDEAAVNYQSVIDQFAVGLKFLNDNFGVCGRPRVGWQIDAFGHSREQASMFAQMAFDGQFFARMDQNDKNKRVDNL  207 d CG9463
89   RRFIQVETAFFAKWWNVQSESMKKVVTKLVNEGRFEFTGGAWSMNDEANVNYQGVIDQFTVGLKFLNETFGSCGRPRVGWQIDTFGHSREQAAIFAHMGYDGQFFSRMDHNDKDTRMNDL  208 d CG9465
88   RRFIQVETSFFSKWWNEQSETMRAVVKMLVNEGRLQFINGAWSMNDEAAVNYQSVIDQFTVGLKFLDDTFGVCGRPRVGWQIDPFGHSREQASIYAQMGFDGEFFSRMDHNDKGRRMNDL  207 d CG9466
89   RRFIQVETSFFAKWWAEQSETAKQVVRKLVNEGRLEFTGGAWSMNDEAAVNYQSVIDQFTLGLKFLDDTFGSCARPRIGWQIDPFGHSREQASIFAQMGYDGEFFARMDHRDKNDRIDNL  208 d CG9468
     :***  **   ** :*:  *.    :  *   ** :**::*   *.* *****  ..*  .::**:   **: *.:.**   .** :.*:** ****** *:::*:*.::* **.*:*   **   *:

234  EMEQVWRASTSLKPPTADLFTGVLPNGYNPPRNLCWDVLCVDQPLVEDPRSPEYNAKELVDYFLNVATAQGRYYRTNHTVMTMGSDFQYENANMWFKNLDKLIRLVNAQQAKGSSVHVLY  353 h Major Lys
173  EMEMIWQSSESLK--NSNIFTGMLYNHYSAPPGFCFDINCEDAPIIDGE-SYDNNVDARVSEFIDYVKNMSKSYRSTHIMVPMGDDFQYEDAAVNFKNMDKLIKYVNDRQSTGSQVNVFY  289 d CG5322
218  NAEMIWHGSANLGE-EADLFSGALYNNYQAPDGFCFDILCNDAPIIDGKHSPDNNVKERIDTFLDFAKTQSQYYRTNNIIVTMGGDFTYQAAQVYYKNLDKLIRYGNERQANGSNINLLY  336 d CG6206
208  GLEMVWDASETLE--EMDFFTGMLYNHYSAPPGFCFDSLCQDDPIIDGK-SYDNNVKSRVDDFLNYASNLAGYYRSNHIMVPMGDDFQYENAYMNYKNMDKLIKYVNERQASGSKYNIFY  324 d CG9463
209  TMEMIWDASESMS--GVSLFSGMLYNFYSDTPGFCFDILCTDQPIIDGD-SEENNVKQRVDDFISYAKNVSEVYITNHIMVPMGGDFQYEDAKVNYKNMDKLIKYINERQASGSKYNIFY  325 d CG9465
208  ALEMIWDASESLS--QVKLFTGLLYTFYWDTPGFCFDVHCSDDPIIDSD-SYDNNVKSRVDDFIAYAAQVAEKFRTNHIMIPMGGDFQYEDAEVNYKNMDKLIKYVNERQSSGSKYNIIY  324 d CG9466
209  GMEMIWDASDSLS--NDEIFTGLLYRHYSAPPGYCFDVHCGDDPIIDTK-SYDNNVKSRVDDFLSYVTSVAQHYRSNHIMIPMGDDFQYEDAQVNFKNMDKLIKYVNERQAEGSTFNLFY  325 d CG9468
       * :* .* .:     .:*:* *    *       *:*   * * *:::    * : *..   :. *:   .    .   : :.. :: ** ** *: * : :**:****:   * :*: **   .::*

354  STPACYLWELNKANLTWSVKHDDFFPYADGPHQFWTGYFSSRPALKRYERLSYNFLQVCNQLEALVGLAANVGPYGSGDSAPLNEAMAVLQHHDAVSGTSRQHVANDYARQLAAGWGPCE  473 h Major Lys
290  STPSCYLYELHQLKQTWPNKTEDFFPYSSDSHSYWTGYFTSRPTQKRFHRDGNHFFQTVKQLSVLANLSGT---QYSEDLDNLSQAMGIMQHHDAVTGTEKQAVASDYDRLLFKAIVGAE  406 d CG5322
337  STPSCYLKSLHDAGITWPTKSDDFFPYASDPHAYWTGYFTSRPTLKRFERDGNHFLQVCKQLSALAPKKPE---EFDPHLTFMRETLGIMQHHDAITGTEKEKVALDYAKRMSVAFRACG  453 d CG6206
325  STAGCYLNSLHKSLQSFPNKTQDFLPHSHEAKSFWTGFFTSRPTQKRFERDGNHILQVAKQLSVLADLSGE---EQHKDLDYLRQIMAVMQHHDAITGTEKQEVSNDYDRLLYDAILGGA  441 d CG9463
326  STPSCYLNSLHQSLESWPNKTQDFFPYAHEKNSFWTGFFTSRPTQKRFERDGNHMLQVAKQLSVLANLTSE---EHKIDLDYLRQVMGVMQHHDAITGTEKQAVSDDYDRMMYEAIVGAS  442 d CG9465
325  STPTCYLNSVHKSVQSYPNKTLDFFPYGSDTNSFWTGYYTSRPTQKRFERDGNHILQVAKQLSAFAELSST---QQKEDLEYLREIMGVMQHHDAITGTEKQHVSDDYDRILYDAIVGGV  441 d CG9466
326  STPACYLNSLHEGLQTWPNKTQDFFPYGSDDNSYWTGYFTSRPTQKRFERDGNHILQVAKQLSVFAELNTK---QQKEDLEYLREIMGVMQHHDAITGTEKQHVSDDYDRILYDAILGGV  442 d CG9468
     **  *** .:..    ::  *  **:*:.    . :***:::***: **:.* . .::*. :**..:.            .    : : :.::*****::**.:: *: ** : :  .

474  VLLSNALARLRGF------------KDHFTFCQQLNISICPLSQ-TAARFQVIVYNPLGRKVNWMVRLPVSEGVFVVKDPNGRTVPSDVVIFPSSD------SQAHPPELLFSAS-LPAL  573 h Major Lys
407  NSARDALRSLTNLT-----------SGEFESCLELNISVCAFTQDTANNVIVTLVNPLAHSSTQYVRVPAKNENYIVTDEKGREVFSEVVPVPWQVLALEHRPNDTQHELVFEASVDK--  513 d CG5322
454  ATTRNALNQLTVQSKDNVKDTSAKYVFEFKTCALLNITSCPVSE-ANDRFALTLYNPLAHTVNEYVRIPVPYSNYRIIDNKGVTLESQAVPIPQVLIDIKHRNSTAKYEIVFLATNIPAL  572 d CG6206
442  NTARDALRVLTNQP-----------NGEFQSCLKLNISECAFTKENADDFMVTLYNPLAHTSTQYVRVPVKEDSYQVTDEKGGLVASELVPVPWEVLSLEFRNNDTQHELVFKASVNK--  548 d CG9463
443  NNARDALRLLTNMP-----------YGEFESCLRLNISECAFTKDGADNVLVTLYNPLAHSSTQYVRIPVKEENYQVTDEKGRTVPSEVVPVPWQVLALEYRSNDTQHELVFKATVNK--  549 d CG9465
442  KTAGDALRKLTNLP-----------NGEFESCLQLNISECAFTKDGADNVVVTLYNALAHTTKQYVRVPVKNENYQVTDEKGRVVASEIVPVPAEVLALEFRDSDTQHELVFKASVDK--  548 d CG9466
443  NTARDGLRKLTNLP-----------NGEFESCLQLNISECAFTKDDADNVVVTLYNPLAHTSTQYVRVPVKNENYEVTDEKGRVVASEVVPVAWQVLALEFRNNDTQHELVFKASVDK--  549 d CG9468
         :.*  *                     .*  *  ***: * .::     . : : * *.:. .  **:*.    : : * :*  : *: * .          .    *::* *:

574  GFSTYSVAQVPRWKPQARAPQP--------------IPR---------------RSWSPALTIENEHIRATFDPDTGLLMEIMNMNQQLLLPVRQTFFWYNASIGDNE--SDQASGAYIF  662 h Major Lys
514  -IANFLIRVLPSPKNIAEDQV--------------Q---------------FPVERSQDELTVETSLVKLTFDTTTGGLKTVKMN--GFTENIQQTFGIYKGYRGNNGESKNRSSGAYVF  601 d CG5322
573  GYRTYYVEKLDSTEGNTRSKA---------------LPK----------------RTSSVTVIGNSHIQLGFDT-NGFLSEVTAD--GLTRLVSQEFLFYEGAVGNNAEFLNRSSGAYIF  658 d CG6206
549  -IATYFIKKIDKTTEVDITVNTQTEQSVNEEESNVKVPKRFKKVHSLKTDLETL--DDQETIVQNSLIKLVIDNNTGRLKTVEMN--GVSEDIDQTFGMYKTAR----------SCHYIF  653 d CG9463
550  -VSSYSIKKVESSEVKHSSIK--------------------------KINPRED--NNSETVVENSVVKLVMDNHTGRLKSVEMN--GVSEKIVQNFGVYKSHT----------SCAYTF  628 d CG9465
549  -IANYYIKKVDSKESSNGVRVI----------------------SQPKANAETY--DDEETIVQTSLIKLVLDNKTGLLKRVEMN--GVSENIEQSYGLYRTYD----------SGAYVF  631 d CG9466
550  -IANYYIKKVASQETKNVAAHTKSKRSIKAEEANLEVPKRFKKVHSLKNATETFDDDEGETVVQTSQVKLVIDNKTGLLKTVEMN--GVSENIDQSYGVYRTYD----------SGAYVF  656 d CG9468
        .: :  :                                          .    : .. ::  :*  .* *  :       .   : * :  *.                  *  * *

663  RPNQQKPLPV-SRWAQIHLVKTPLVQEVHQNFSAWCSQVVRLYPGQRHLELEWSVGPIPVGDTWGKEVISRFDTPLETKGRFYTDSNGREILERRRDYRPTWKLNQTE-PVAGNYYPVNT  780 h Major Lys
602  RPY--GDIEIVNNKVELSFYNGTKVKEVHQHVNEWISQVIRIYEDVNRVEFEWLVGPIPTDDDVGKEIITRFSSNISSKGKFYTDSNGREILERERNQREHFTPDMSE-AISGNYYPVTG  718 d CG5322
659  RPNE-NKIHFATDQVEIEVYKGDLVHEVHQKFNDWISQVVRVYNKDSYAEFEWLVGPIPIDDGIGKEVITRFNSDIASDGIFRTDSNGREMIKRKINHRDTWSVKINE-AVAGNYYPITT  776 d CG6206
654  RQD--ADLELVEDAFDFTVYDGESVKEVHQHVNEWISQVIRIYEGVNRVEFEWLVGPVPIDDELGKEIVTIFKSGISSGGVFYTDSNGREMMRREKDKREDFSPDLSEQPVSGNYYPVTS  771 d CG9463
629  RQD--GDIELFENDFEFTVYEGSLVKEVHQHVNDWISQVIRIYEGVSRVELEWMVGPIPIDDKLGSEFVTNFKSEISSNGVFYTDSNGRELMKRVKDKREDFVSDLSRQPVSGNFYPVTS  746 d CG9465
632  RQYNQGDFVIQEDGVEFTVYDGALVKEVHQRFSEYISQVIRISEDKPYVEFEWLVGPIPVEEEFGTEVVTIFSSEIASNGVFYTDSNGRELIRREKDKREDFTPELAVQPTSGNYYPITS  751 d CG9466
657  RQYHQADFIVQYEGVEFTVYDGALVKEVHQQFSEYISQVIRIYEGKNLVEIEWQVGPIEREEEFGREVVIIFNSTIASDGVSYTDSNGREMIKRVKDQRETFTPGLDRQPTAANYYPVTS  776 d CG9468
     *      : .     :: . .   *:****... : ***:*:       *:** ***:   :  * *.:   *.: : : *   *******::.*  : *  :           :.*:**:.
```

```
781  RIYITDGNMQLTVLTDRSQGGSSLRDGSLELMVHRRLLKDDGRGVSEPLMEN--GSGAWVRGRHLVLLDTAQAAAA----GHRLLAEQEVLAPQVVLAP-GGG-AAYNLGAPPRTQFSGL   892   h Major Lys
719  QISLQDDEKRITLLNDRAQGGTSLKDGELELMLHRRLLNDDAFGVGEALNETQYGTGLIARGKIYLILDAVDG--K----PNQRLLQHQLDQHFWKFFSKSNGVASVNRNM-----I-PD   826   d CG5322
777  KIDVEDDTARMAILTDRAQGGSSLKDGSLELMVHRRLLKDDAFGVGEALNETEYGDGLIARGKHHLFFGKSTDREGVSLKGIERLTQLEKLLPTWKFFSNMEDYSADEWQTAFTNIFSGI   896   d CG6206
772  RMALQDSSKRMVLLNDRSQGGASLEDGRLEMMLHRRHIFADGSGAAEAINEQQFGKGLIARGKLFLYLNAIEDGAT----ASERVAEKEIHLPFWKFFSKSNNIQSDVTKT-----L-SD   881   d CG9463
747  RIALQDDTKRLVLLNDRSQGGASLEDGALEMLIHRRHLFNDGGGVGEALNETQYGKGLIARGKLYLILDSATDGDT----VTERKTEKELFLPFWKFFSKTGGVEITPSKS-----L-PD   856   d CG9465
752  RIALQDSNKRLAILNDRAQGGTSMKDGQIELMLHRRLVRDDGYGVGEALNEEKYGQPLIARGKVFLILNAA-DEST----SAEREAEKEFHLPLWKFFSKNTGSTTAAAKS-----V-PS   860   d CG9466
777  RIALQDSKKRIAVLNDRAQGGASMLNGQLELMLHRRLVRDDGYGVGEALNEEKYGQPMIARGKVYLILSPS-DEST----AAEREAEKEIHLPFWKFFSKNTGSTTAAAKS-----V-PS   885   d CG9468
     :: : *   ::.:*.**:***:*: :* :*:::*** :  *. *..* : *   *    .**:  : :              .   : :       :                   .
893  RRDLPPSVHLLTLASWGPEMVLLRLEHQFAVGEDSGRNLSAPVTLNLRDLFSTFTITRLQETTLVANQLREAASRLKWTTNTGP-TPHQTPYQL--------------------------   985   h Major Lys
827  FFGIPESVELLSLEPYSKDQILIRLENFN--TE------GNVVSFNIYPLFESLDGYQIWETTLDGNMLLEDVKRFKFAQDGTGSIPSSVEYYH---------APHNPLTA---------   920   d CG5322
897  SLVLPKPVHLLTLEPWHENQLLVRFEHIMENGEDA--SYSQPVQFNVKNVLSAFDVEGIRETTLDGNAWLDESRRLQFAPDPEEAAFNTYATFSQPAESVHLLSAEKPMLGVKYADEALP  1014   d CG6206
882  FNDLPQSVHLLTLEPYSKDEILLRLENFLDQTE------GNVVSFSIRQIFDDLGGLEIRETTLDGNLPLSDMKRLKFHHDGSGPSHSVPEYFT---------SLHKPLAA---------   977   d CG9463
857  FNDLPQSVHLLTLEFFSEQEILIRFEHFLDKSE------GRVISFNIRDIFDSLGGLAIRETTLDGNMPLSDMKRFKFHAQESGTKPSSVEYST---------AQHKPLEA---------   952   d CG9465
861  FDDFPKSVHLLTLEPFNDDEILLRVENFKDHIE------GKVVSFNIRPIFDYLNGVEIRETTLDGNLPLSDMKQFKFHAEGSGIRGSEPEYYT---------SSHKPLSA---------   956   d CG9466
886  FNDFPKSVHLLTLEPFNDDEVLLRVENFLDHTE------GQVVSFNIRPIFDYLNGVEIRETTLDGNLPLSDMKRFKFHHDSSGQKPDAVEYFT---------SAHKPLAA---------   981   d CG9468
        :*  *.**:*  :  : :*:*.*.     *      .  : :.:  ::. :    : **** .*   .   ::::  :
986  --------------------------------------------DPANITLEPMEIRTFLASVQWKEVDG  1011   h Major Lys
921  --------------------------------------NSTMNASGFVVTLVPMQIRTFIIQQKYIPS--   950   d CG5322
1015 AGQLGAESNRIRRETETRQEKKDEGRSSKSTEGPYNSFKSDSSNQEYIIELSPMEIRTFIVYLTPA----  1080   d CG6206
978  --------------------------------------DKTQDASEFSVTLKPMQIRTFIIKKE------  1003   d CG9463
953  --------------------------------------VKSDEASLFAVTLYPMQIRTFIIKTE------   978   d CG9465
957  --------------------------------------NQTQDAAEFAVTLYPMQIRTFIIKHE------   982   d CG9466
982  --------------------------------------EQSQEASEFSVTLHPMQIRTFIIKTE------  1007   d CG9468
                                                     : * **:****:
```

Supporting References

S36. Venkatesan M, Kuntz DA, Rose DR (2009) Human lysosomal alpha-mannosidases exhibit different inhibition and metal binding properties. Protein Sci 18: 2242-2251.

S37. Howard S, He S, Withers SG (1998) Identification of the active site nucleophile in jack bean alpha-mannosidase using 5-fluoro-beta-L-gulosyl fluoride. J Biol Chem 273: 2067-2072.

S38. Numao S, He S, Evjen G, Howard S, Tollersrud OK, et al. (2000) Identification of Asp197 as the catalytic nucleophile in the family 38 alpha-mannosidase from bovine kidney lysosomes. FEBS Lett 484: 175-178.

S39. Stensland E, Lindal S, Jonsrud C, Torbergsen T, Bindoff LA, et al. (2011) Prevalence, mutation spectrum and phenotypic variability in Norwegian patients with Limb Girdle Muscular Dystrophy 2I. Neuromuscul Disord 21: 41-46.
