# Supplementary material for: Mutations in Four Glycosyl Hydrolases Reveal a Highly Coordinated Pathway for Rhodopsin Biosynthesis and N-Glycan Trimming in Drosophila melanogaster
Source: PLoS Genet. 2014 May 1;10(5):e1004349. doi: 10.1371/journal.pgen.1004349 (PMC4006722; doi:10.1371/journal.pgen.1004349)
Supplement: Figure S6 — Hexosaminidases (GH Family 20). (A) Phylogenetic tree depicting the predicted evolutionary relationships between the hexosaminidases from GH Family 20 in humans (h) and Drosophila (d), generated with the UniProt Align program using the GenBank sequence accession numbers listed in Figure 2. This list includes the human β-hexosaminidase, α-chain (HEXA), the human β-hexosaminidase, β-chain (HEXB), Drosophila hexosaminidase 1 (Hexo1, CG1318), Drosophila hexosamindase 2 (Hexo2, CG1787), and Drosophila fused lobes (Fdl, CG8824). Black arrows designate speciation of the last common ancestor between humans and flies, leading to the production of orthologs. White arrows denote presumed gene duplication events, leading to the production of paralogs. Based on this analysis, there was likely a single hexosaminidase in the last common ancestor between humans and Drosophila, thus the human gene pair and the Drosophila gene triplet have evolved separately through independent duplication events. (B) Full-length amino acid (aa) alignments between evolutionarily related human (h) and Drosophila (d) hexosaminidases from GH Family 20, generated with the UniProt Align program using the GenBank sequence accession numbers listed in Figure 2. This list includes the human β-hexosaminidase, α-chain (HEXA), the human β-hexosaminidase, β-chain (HEXB), Drosophila Hexo1 (CG1318), Drosophila Hexo2 (CG1787), and Drosophila fused lobes (CG8824). Identical amino acids are marked with asterisks (*), strongly similar amino acids are marked with two dots (:), and weakly similar amino acids are marked with one dot (.). Underlined regions represent predicted transmembrane domains (TMHMM Server v.2.0). All pair-wise comparisons between the human and Drosophila proteins yield 30–32% overall aa identity and 35–38% aa identity within the GH Family 20 catalytic domain (d Hexo1 aa214–557, d Hexo2 aa235–581, and d fused lobes aa276–613). Crystallization, photoaffinity labeling, expression of chimeric subunits, [file pgen.1004349.s006.pdf]

Figure S6. Hexosaminidases (GH Family 20)

A

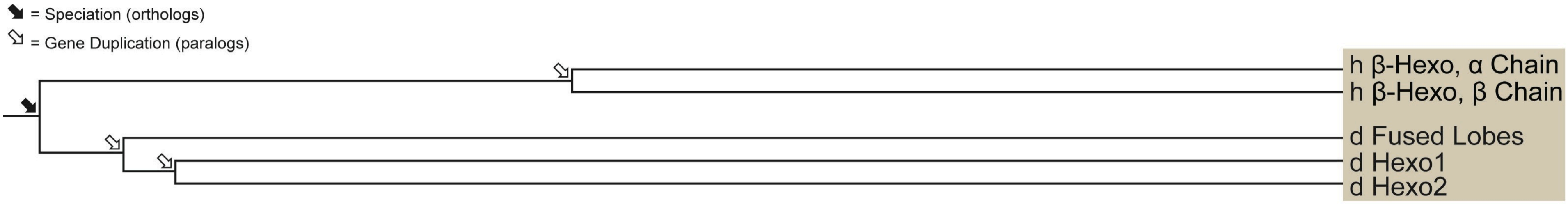

B

```
1   --------------------------------------------------------------------------------------MTSSRLWFSL----LLAAAFAG-RATALWPWPQN 29  h β-Hexo, α chain
1   MELCGLGL-------PRPPMLLALLL-------------------------------------------------ATLLAAMLAL-LTQVALVVQVAEAARAPSVSAK-PGPALWPLPLS 62  h β-Hexo, β chain
1   --------MKSTRTALGVALLLALVSQLA-----------AHS------------SDDLVYGYECRSGYCQKVELSEE--------NYVK------AISLPVCRLFCGSSIGTLWPKPTG 75  d Hexo1
1   MRFSGYNRYQCFCSAVGSLLLLSLLSFAVGAA--------LTRAD--------DVGSDADAGSASRKWLCSRTDICTAEGEMVAGLQYAPEI----FESQRDCRLSCGK-YGAIWPMPTG 99  d Hexo2
1   MSLAVSLRRALLVLLTGAIFILTVLYWNQGVTKAQAYNEALERPHSHHDASGFPIPVEKSWTYKCENDRCMRVGH-----------HGKSAK----RVSFISCSMTCGD--VNIWPHPTQ 103 d Fused Lobes
                                                                                                                 .   :**  *

30  FQTSDQRY---VLYPNNFQFQYDVSSAAQPGCSVLDEAFQRYRDLLFGSGS-------------------WPRPYLTG-----------KRHTLEKNVLVVSVVTPGCNQLPTLESVENY 116 h β-Hexo, α chain
63  VKMTPNLL---HLAPENFYISHSPNSTAGPSCTLLEEAFRRYHGYIFGFYK-------------------WHHEPAEF-----------QAKTQVQQLLVSITLQSECDAFPNISSDESY 149 h β-Hexo, β chain
76  TVRLDTLMRQVDISFIDFNFN--GIARQQ---KLWRAVEDRFMN-MLE------------AQIPD--------------RKVL------ARGGYRMSVNI-NTPDEPTPARLTLDTDESY 156 d Hexo1
100 KECTISH-RRVRFDPWKVRFH--VVAPGEAATQFLRETNRLFVSNLLK------------ECIRN--------------CTLE------T--SKQI--LV-RSTVANESLVLDWPTDESY 179 d Hexo2
104 KFLLSSQTHSFS--VEDVQLH--VDTAHREVRKQLQLAFDWFLKDLRLIQRLDYVGSSSEPTVSESSSKSRHHADLEPAATLFGATFGVKKAGDLTSVQVKISVLKSGDLNFSLDNDETY 219 d Fused Lobes
                  .. :    :          .   :   :                                                :                      . *.*

117 TLTINDDQ-----CLLLSETVWGALRGLETFSQLVWKSAE--GTFFINKTEIEDFPRFPHRGLLLDTSRHYLPLSSILDTLDVMAYNKLNVFHWHLVDDPSFPYESFTFPELMRKGSYNP 229 h β-Hexo, α chain
150 TLLVKEPV-----AVLKANRVWGALRGLETFSQLVYQDSY--GTFTINESTIIDSPRFSHRGILIDTSRHYLPVKIILKTLDAMAFNKFNVLHWHIVDDQSFPYQSITFPELSNKGSYS- 261 h β-Hexo, β chain
157 TLDIDTDASGHVLANITASNFFGARHGLETLAQLIVYDDIRREVQVTANATINDAPVYKWRGLLLDTSRNYYSVKSIKRTLEGMALVKLNTFHWHITDSHSFPLEVKKRPELHKLGAYS- 275 d Hexo1
180 ALVVRTTET-ATFVDIQATTVYGARHAFETLSNLVT-GSLSNGLLMVTTANITDRPAFSHRGVLLDTARNFVPLKFIRSTLDAMAASKLNVLHWHVVDTHSFPLEITRVPEMQRYGAYS- 296 d Hexo2
220 QLSTQTEGH-RLQVEIIANSYFGARHGLSTLQQLIWFDDEDHLLHTYANSKVKDAPKFRYRGLMLDTSRHFFSVESIKRTIVGMGLAKMNRFHWHLTDAQSFPYISRYYPELAVHGAYS- 337 d Fused Lobes
     *             : :    :** :.:.*: :*:              : : * * :   **:::**:*.:   :. *   *:   *.   *:* :***:.*   ***          **:    *:*.

230 VTHIYTAQDVKEVIEYARLRGIRVLAEFDTPGHT---LSWGPGIP-GLLTPCYSGSEP-----SGTFGPVNPSLNNTYEFMSTFFLEVSSVF-PDFYLHLGGDEVDFTCWKSNPEIQDFM 339 h β-Hexo, α chain
262 LSHVYTPNDVRMVIEYARLRGIRVLPEFDTPGHT---LSWGKGQK-DLLTPCYSRQN-----KLDSFGPINPTLNTTYSFLTTFFKEISEVF-PDQFIHLGGDEVEFKCWESNPKIQDFM 371 h β-Hexo, β chain
276 QRQVYTRRDVAEVVEYGRVRGIRVMPEFDAPAHVGEGWQH------KNMTACFNAQPWKSFCVEPPCGQLDPTVNEMYDVLEDIYGTMFDQFNPD-IFHMGGDEVSTSCWNSSQPIQQWM 388 d Hexo1
297 SSQTYSRQDALNLVKYARLRGIRILIEIDGPSHAGNGWQWGPAAGLGNMSVCLNQSPWRRFCVQPPCGQLNPLNDHMYAVLKEIFEDVAEVGAPEETLHMGGDEVFLPCWNNTDEIRDGM 416 d Hexo2
338 ESETYSEQDVREVAEFAKIYGVQVIPEIDAPAHAGNGWDWGPKRGMGELAMCINQQPWSFYCGEPPCGQLNPKNNYTYLILQRIYEELLQHTGPTDFFHLGGDEVNLDCWAQY------- 450 d Fused Lobes
       . *: .*.  : ::.:: *:::: *:* *.*.      .            :: *  . .                *  ::*   :   *  .:   ::   :  .        :*:*****    ** .

340 RKKGFGE---DFKQLESFYIQTLLDIVSSYGKG----------YVVWQEVFDNKV-----KIQPDTIIQVWREDIPVNYMKELELVTKAGFRALLS--APWYLNRISYG----------- 428 h β-Hexo, α chain
372 RQKGFGT---DFKKLESFYIQKVLDIIATINKG----------SIVWQEVFDDKA-----KLAPGTIVEVWKDS---AYPEELSRVTASGFPVILS--APWYLDLISYG----------- 457 h β-Hexo, β chain
389 KKQGWGLETADFMRLWGHFQTEALGRVDKVANG------THTPIILWTSGLTEEPFIDEYLNPERYIIQIWTTGVDPKVK----KILERGYKIIVSNYDALYLDCGGAGWVTDGNNWCSP 498 d Hexo1
417 RARGYDLSEQSFLRLWSQFHQRNLNAWDEINERMYPGIKEPKSVIIWSSHLTNPRYIETYLPKERFIIQTWVESQDALNR----ELLQRGYRLIVSTKNAWYLDHGFWG--------STS 524 d Hexo2
451 ------FNDTDLRGLWCDFMLQAMARLKLANNGV-----APKHVAVWSSALTNTK----CLPNSQFTVQVWGGSTWQENY----DLLDNGYNVIFSHVDAWYLDCGFGSWRATGDAACAP 551 d Fused Lobes
               .:  *     :     :       :          :* . : :            :: *              :   *:  :.*     **:     .

429 -PDWKDFYVVEPLA-FEGTPEQKALVIGGEACMWGEYVDNTNLVPRLWPRAGAVAERLWSNKLTSDLT-----FAYERLSHFRCELLRRGVQAQPLNVGFCEQEFEQT--------- 529 h β-Hexo, α chain
458 -QDWRKYYKVEPLD-FGGTQKQKQLFIGGEACLWGEYVDATNLTPRLWPRASAVGERLWSSKDVRDMD-----DAYDRLTRHRCRMVERGIAAQPLYAGYCNHENM----------- 556 h β-Hexo, β chain
499 YIGWQKVYDNSLK---SIAGDYEHHVLGAEGAIWSEQIDEHTLDNRFWPRASALAERLWSNPAEGWRQ------AESRLLLHRQRLVDNGLGAEAMQPQWCLQNEHECPIDAYDAQV 606 d Hexo1
525 YYNWRTVYSSGMP-----VGRSKDQVLGGEVCMWSEYVDQNSLESRIWPRAGAAAERMWSNPKSSALL------AQRRFYRYRERLLARGIHADAVIPHWCVLHEGQCL-------- 622 d Hexo2
552 YRTWQNVYKHRPWERMRLDKKRKKQVLGGEVCMWTEQVDENQLDNRLWPRTAALAERLWTDPSDDHDMDIVPPDVFRRISLFRNRLVELGIRAEALFPKYCAQNPGECI-------- 660 d Fused Lobes
       *:   *               :   .:*.*  .:* * :*    *  *:***:.*  .**:*:.              .  *:   .*  .::   *: *:  :    :*   .
```

Supporting References

S40. Brown CA, Mahuran DJ (1991) Active arginine residues in beta-hexosaminidase. Identification through studies of the B1 variant of Tay-Sachs disease. J Biol Chem 266: 15855-15862.

S41. Lemieux MJ, Mark BL, Cherney MM, Withers SG, Mahuran DJ, et al. (2006) Crystallographic structure of human beta-hexosaminidase A: interpretation of Tay-Sachs mutations and loss of GM2 ganglioside hydrolysis. J Mol Biol 359: 913-929.

S42. Liessem B, Glombitza GJ, Knoll F, Lehmann J, Kellermann J, et al. (1995) Photoaffinity labeling of human lysosomal beta-hexosaminidase B. Identification of Glu-355 at the substrate binding site. J Biol Chem 270: 23693-23699.

S43. Maier T, Strater N, Schuette CG, Klingenstein R, Sandhoff K, et al. (2003) The X-ray crystal structure of human beta-hexosaminidase B provides new insights into Sandhoff disease. J Mol Biol 328: 669-681.

S44. Mark BL, Mahuran DJ, Cherney MM, Zhao D, Knapp S, et al. (2003) Crystal structure of human beta-hexosaminidase B: understanding the molecular basis of Sandhoff and Tay-Sachs disease. J Mol Biol 327: 1093-1109.

S45. Pennybacker M, Liessem B, Moczall H, Tifft CJ, Sandhoff K, et al. (1996) Identification of domains in human beta-hexosaminidase that determine substrate specificity. J Biol Chem 271: 17377-17382.

S46. Pennybacker M, Schuette CG, Liessem B, Hepbildikler ST, Kopetka JA, et al. (1997) Evidence for the involvement of Glu-355 in the catalytic action of human beta-hexosaminidase B. J Biol Chem 272: 8002-8006.

S47. Sharma R, Bukovac S, Callahan J, Mahuran D (2003) A single site in human beta-hexosaminidase A binds both 6-sulfate-groups on hexosamines and the sialic acid moiety of GM2 ganglioside. Biochim Biophys Acta 1637: 113-118.
