# Supplementary material for: Mutations in Four Glycosyl Hydrolases Reveal a Highly Coordinated Pathway for Rhodopsin Biosynthesis and N-Glycan Trimming in Drosophila melanogaster
Source: PLoS Genet. 2014 May 1;10(5):e1004349. doi: 10.1371/journal.pgen.1004349 (PMC4006722; doi:10.1371/journal.pgen.1004349)
Supplement: Figure S7 — β-Mannosidases (GH Family 2). Full-length amino acid (aa) alignment between human (h) β-mannosidase (MANBA) and the Drosophila (d) homolog, CG12582, from GH Family 2, generated with the UniProt Align program using the GenBank sequence accession numbers listed in Figure 2. Identical amino acids are marked with asterisks (*), strongly similar amino acids are marked with two dots (:), and weakly similar amino acids are marked with one dot (.). Drosophila CG12582 displays 34% overall aa identity with human β-Man and 51% aa identity within the GH Family 2 TIM barrel domain (Drosophila aa343–457). Crystallization and site-directed mutagenesis of a β-mannosidase from Bacteroides thetaiotaomicron have revealed a number of critical residues that are highly conserved among all members of GH Family 2 [S48]. Yellow shading indicates the two catalytic nucleophiles. Purple shading denotes other critical residues that either contribute to the enzyme's catalytic activity or are important for substrate recognition and binding. Finally, light blue shading highlights four missense mutations that cause β-mannosidosis in humans [30]. Importantly, all of these key residues are conserved in the putative Drosophila β-mannosidase (CG12582). (PDF) [file pgen.1004349.s007.pdf]

Figure S7. β-mannosidases (GH Family 2)

```
1     ---------------MRLHLLLLLALCGAGTTAAELSYSLRGNWSICNGNGSLELPGAVPGCVHSALFQQGLIQDSYYRFNDLNYRWVSLDNWTYSKEFKIPFEISKWQKVNLILEGVDT  105  h β-Man
1     MKYGCGLLRNGTTGAVMVGLVCGLYVCSGQ--CDKVEVIELSNWTITNQNGSLTIANQTLPLGIYS----AFAPQVLDSYNDVGLRYLAYDNFTFSNFFQFDVHHFNRGHINLTFHGIDT  114  d CG12582
                     : : *:  * :*..   . ::.    .**:* * **** :   .       :    .:  :    :**:  *::: **:*:*: *:: ..  :  ::** :.*:**
106   VSKILFNEVTIGETDNMFNRYSFDITNVVRDVNSIELRFQSAVLYAAQQS--KAHTRYQVPPDCPPLVQKGECHVNFVRKEQCSFSWDWGPSFPTQGIWKDVRIEAYNICHLNYFTFSPI  223  h β-Man
115   VSEIRLNHQLLGRTDNMFVRYSYEVSSLLQAENFLEVEIQSPVVAALARANALKEAKKSVPPECPNERYHGECHMNMLRKMQASFSWDWGPAAPSAGIWKNVELEIYEVAVIREVDVD-V  233  d CG12582
      **:* :*.  :*.***** ***::::.:::  * :*:.:** *: *   ::    .:: .***:**    :****:*::** *.********: *: ****:*.:* *::. :. . .. :
224   YDKSAQEWNLEIESTFDVVSSKPVGGQVIVAIPKLQT----QQTYSIELQPGKRIVELFVNISKNITVETWWPHGHGNQTGYNMTVLFEL---------DGGLNIEKSAKVYFRTVELIE  330  h β-Man
234   SRVNGSYWNMHIRCFLDAGGWQNFNGRLVLYAVELLDRPVVVDRYAIKNISHLAPVIEFDQAVPNGKVVTWWPNGYGEQKLYPLHFTLKTWLGPDGPEVRSKTKSHKSLRVGFRTLDLVE  353  d CG12582
         ... **:.*.. :*. . : . *::::   :*        : *:*:        *  * :   * .* ****.*:*:*. * : . ::             .  : .** :* ***::*:*
331   EPIKGSPGLSFYFKINGFPIFLKGSNWIPADSFQDRVTSELLRLLLQSVVDANMNTLRVWGGGIYEQDEFYELCDELGIMVWQDFMFACALYPTDQGFLDSVTAEVAYQIKRLKSHPSII  450  h β-Man
354   DPAPDGVGNTFLFRVNGVEMFMKGSNYIPSHLLPEMQTNEQISHLLRSAKEAHMNMLRVWGGGVYESDYFYQLADSLGLLIWQDMMFACAMYPVNDEFLSSVREEVRQNAMRLSHHPSVA  473  d CG12582
      :*    . * :* *::**. :*:****:**:. : :   *.* :  **:*. :*.** *******:**.* **:*.*.**:::***:*****:**.:: **.**  **   :   **. ***:
451   IWSGNNENEEALMMNWYHISFTDRPIYIKDYVTLYVKNI-RELVLAGDKSRP-FITSSPTNGAETVAEAWVSQNPNSNYFGDVHFYDYISDCWNWKVFPKARFASEYGYQSWPSFSTLEK  568  h β-Man
474   IFVTNNENEAALVQNWYGTLFERD-RFESEYRELYLANVIHELKLVSHSSRPQPLVSSPSNGKASEPDNYISSNPQDNQNGDVHFYDYTKDGWDPGIFPRPRFVSEFGFQSFPGAYAWQR  592  d CG12582
      *:  ***** **: ***    *      : .:*  **: *: :** *....***  :.***:**  :  : ::*.**:.*  ******** .* *:  :**: **.**:*:**:*.  : ::
569   VSSTEDWSFNSKFSLHRQHHEGGNKQMLYQAGLHFKLPQSTDPLRTFKDTIYLTQVMQAQCVKTETEFYRRSRSEIVDQQGHTMGALYWQLNDIWQAPSWASLEYGGKWKMLHYFAQNFF  688  h β-Man
593   SKGEDDDL--LGLITHRQHHPLGNVPVIALVERHLPLPFPENE-NYATALIYFSQIAQAMATKVETELYRSLR----DTPHRTMGALYWQLNDVWVAPSWSSIDFYGNWKLLHYWARDFL  705  d CG12582
       .. :*      :  *****  **  ::  .  *: **   :  .  .  **::*: ** ..*.***:**  *    *   :***********:* ****:*::: *:**:***:*::*:
689   APLLPVGFENENM-----FYIYGVSDLHSDYSMTLSVRVHTWSSLEPVCSRVTERFVMKGGEAVCLYEEPVSELLRRCGNCTRESCVVSFYLSADHELLSPTNYHFLSSPKEAVGLCKAQ  803  h β-Man
706   APISIVALYEKSTDSLNISLICDQLEV-DPRELNVVANVHLWSQLLPRQSTSWDVTLRPNGV---QYDKVIAISDLLQGEFTRTNAFLELQLRRGQDVISRTHF-FPSSIAGAVGIEDPG  820  d CG12582
      **:  *.: ::.         *    :: .  .:.: ..** **.* *  *   :  :   *     *:: ::      *: ** ...:.: *   ::::* *.: * **   ***: .
804   ITAIISQQG---------DIFVFDLETSAVAPFVWLDVG-SIPGRFSDNGFLMTEKTRTILFYPWEPTSKNELEQ-SFHVTSLTDIY-  879  h β-Man
821   LEFEIASRTCLNTTNVVRNSVSISIQVKRPAVFVYLELLKPYRYTLSENGFMQTEPMHVVYLTFDAPLCARLLRKSDLRVLTVNDFMR  908  d CG12582
      :   *:.:          : . :.::..  * **:*::       :*:***: **  :.: :    * . . *.: .::* ::.*:
```

Supporting References

S48. Tailford LE, Money VA, Smith NL, Dumon C, Davies GJ, et al. (2007) Mannose foraging by *Bacteroides thetaiotaomicron*: structure and specificity of the beta-mannosidase, BtMan2A. J Biol Chem 282: 11291-11299.
